# Chemical topology of lipo-amino fatty acid mRNA carriers triggers distinct lipid bulk phases and endosomal escape mechanisms^☆^

**DOI:** 10.1016/j.mattod.2026.103353

**Published:** 2026-08

**Authors:** Melina Grau, Ekaterina Kostyurina, Johanna Seidl, Nora Martini, Benjamin Winkeljann, Olivia M. Merkel, Joachim O. Rädler, Ernst Wagner

**Affiliations:** a Pharmaceutical Biotechnology, Department of Pharmacy, Ludwig-Maximilians-Universität München, Butenandtstrasse 5-13, Munich 81377, Germany; b Faculty of Physics, Ludwig-Maximilians University, Geschwister-Scholl-Platz 1, Munich, Germany; c Pharmaceutical Technology and Biopharmaceutics, Department of Pharmacy, Ludwig-Maximilians-Universität München, Butenandtstrasse 5-13, Munich 81377, Germany; d Center for NanoScience (CeNS), Ludwig-Maximilians-Universität München, Munich 80799, Germany; e Comprehensive Pneumology Center (CPC-M), Ludwig-Maximilians-Universität München, Member of the German Center for Lung Research (DZL), Munich 81377, Germany; f Pharmaceutical Engineering and Technology Research Scientists (PETRS), Germany

**Keywords:** Molecular dynamics, Nanocarriers, Polyplexes, SAXS, Ultrastructure

## Abstract

LAF-Stp xenopeptides (XPs) comprising lipoamino fatty acid (LAF) units connected with the tetraethylene pentamine derived polyamino acid Stp were identified as a novel class of amphiphilic mRNA carriers. A ‘chameleon-like’ polarity switch is observed upon transition from neutral physiological to endosomal acidic pH. Protonation of the lipidic tertiary amine of LAF results in an ~2 log unit change in the octanol/water partition coefficient (logD), consistent with all-atom molecular dynamics simulations. mRNA delivery efficacy is tunable by the chemical topology (either a flexible ‘U-shape’ or a constrained ‘Bundle’ structure) and specific modifications in the LAF-Stp linker domains. Specifically, replacing lysine with shorter ornithine linkers further increases the structural rigidity of Bundles. For all LAF-XPs, protonation-induced solubilization of carriers triggers endosomal destabilization (evidenced by galectin-8 recruitment) and successful cytosolic mRNA transfection. Carriers with U-shape topology strongly depend on endosomal acidification, as demonstrated by an up to ~20-fold drop in efficacy upon treatment with v-ATPase inhibitor bafilomycin, similar as found for the ionizable polymer polyethylenimine. In contrast, Bundles are far less affected (<4-fold reduction) by bafilomycin treatment. Small-angle X-ray scattering (SAXS) reveals that lipid bulk phase structures are largely pH-independent for both topologies. Interestingly, U-shapes form lamellar phases, whereas Bundle topology induces non-lamellar bicontinuous phases with negative membrane curvature. These findings suggest distinct endosomal escape mechanisms for Bundle and U-shapes. Both carriers benefit from endosomal protonation and solubilization, but only Bundles can adopt pH-independent membrane-destabilizing fusogenic phases, reflected by faster kinetics and characteristic cellular morphology in a galectin-8 assay.

## Introduction

Messenger RNA (mRNA) therapeutics critically depend on efficient delivery to unleash their therapeutic effect. Extracellularly, mRNA requires shielding against rapid degradation and successful cellular uptake. Once internalized, several intracellular barriers must be overcome, with endosomal escape displaying a major hurdle, to achieve cytosolic cargo release. Over the past decades, extensive research has yielded a variety of different RNA delivery platforms to address these challenges [1–5]. Lipoplexes and lipid nanoparticles (LNPs) [6–15] have proven particularly effective, with LNPs successfully translated into therapeutic drugs [16–18] and vaccines [19,20]. Polyplexes, in which cationic polymers compact negatively charged nucleic acids, represent another promising delivery strategy [21–24]. Among polycationic agents, pH-responsive polymers like polyethylenimine (PEI) have shown encouraging activity *in vitro* and *in vivo* [25–28]. However, PEI derivatives are structurally complex and often associated with significant toxicity [29]. Furthermore, medium sized amphiphilic hybrid structures combining positive aspects of LNPs and polymers have also been recently studied [30–33].

To combat the limitations of large cationic polymers, small artificial oligoamino acids such as succinoyl tetraethylene pentamine (Stp), containing the PEI-derived amino ethylene motif, were developed and incorporated into medium-sized sequence-defined xenopeptides (XPs) by solid-phase assisted peptide synthesis [34]. Subsequent modifications with lipophilic moieties, such as conventional fatty acids, yielded lipo-XPs that stabilize nanoparticles via hydrophobic interactions [35]. The chain length, degree of saturation or modifications of the fatty acids strongly impact physicochemical and biological properties of the resulting polyplexes [36–38]. Lipo amino fatty acids (LAFs), containing an ionizable tertiary nitrogen positioned within a branched hydrophobic domain, proved as especially effective and resulted in a novel class of double pH-responsive LAF-Stp XP carriers [32,39]. A chemical evolution of these carriers, combining ionizable lipophilic LAF and polar Stp units in different ratios and with altering branching linkers into different topologies, empirically identified Bundle (B2, Stp:LAF = 1:4) and U-shape (U1, Stp:LAF = 1:2) topologies as remarkably potent in nucleic acid delivery. LAF-XPs maintained high transfection activity even at low dose and in full serum. When administered intravenously, polyplexes mediated efficient mRNA expression in multiple organs or genome editing in case of Cas9 mRNA/sgRNA [32,40]. Bundle XPs also mediate high *in vivo* efficacy as mRNA or siRNA LNPs [41,42]. Both Bundle and U-shape topologies mediate fast and high endosomal rupture, evidenced in transfected cells containing a fluorescent Galectin-8 sensor [32]. The superior endosomal destabilization was also demonstrated with LAF-XP siRNA LNPs in head-to-head comparison with MC3 LNPs [42]. Mitigating the endosomal escape bottleneck in delivery appeared as the main reason for the high potency of LAF-XP carriers even at low concentration, but the underlying mechanism was not verified. The aim of our current work was to provide a better understanding of the endosomal escape by connecting chemical carrier topologies with ultrastructure and bioactivity.

Various mechanisms of endosomal escape have been proposed for different carrier classes [43–46]. PEI-based polyplexes and derivatives rely on endosomal protonation which increases polymer solubility and cation density, ultimately promoting endosomal membrane interaction and destabilization [47–53]. Lipoplexes trigger transmembrane pore formation, for example through mixing of cationic lipids with anionic endosomal lipids [54]. Related processes are hypothesized for LNPs; at physiological pH ionizable lipids are insoluble in water but become cationic and water-soluble upon protonation within endosomes. These protonated lipids mix with endosomal anionic lipids and form cone-shaped ion pairs that trigger lipid phase transition from lamellar bilayer to an inverted hexagonal phase (H_II_),destabilizing endosomes [55–57]. Small-angle X-ray scattering (SAXS) has emerged as a powerful tool to probe structural arrangements and molecular organization in lipid- and polymer-based systems [13,58–65]. SAXS studies of ionizable lipids (e.g. MC3, ALC3) revealed pH-dependent phase transitions from inverse micellar to inverse hexagonal structures, linking ultrastructural features to endosomal escape and subsequent mRNA translation [66,67]. LAF-XPs are far larger macromolecular lipo-peptides in comparison to standard ionizable lipids, and mechanistic similarities and differences of membrane interaction and disruption were only hypothesized.

In the current work, starting from two potent established LAF-XPs, we designed four U-shape and four Bundle XP structures to evaluate the impact of pH on distinct topologies, altered linkers and different LAF-types. To obtain molecular-level mechanistic insight, we combined all-atom molecular dynamics (MD) simulations with high-resolution synchrotron SAXS, complemented by physicochemical, TEM and biological characterization. Previous MD work majorly focused on membrane interactions of smaller conventional ionizable lipids or self-assembly of lipid nanoparticles, and SAXS has been widely used to study the internal organization of LNPs and the role of ionizable lipids in endosomal escape [68,69]. To our knowledge, an integrative strategy of MD and SAXS has so far not been applied to larger, amphiphilic (xeno) peptides. Our findings provide novel insights into this new class of endosomolytic LAF-XP agents. MD simulations elucidate protonation-induced reorganization of these molecules at hydrophilic-lipophilic interfaces and their amphiphilicity driven self-assembly. SAXS measurements identified distinct, largely non-predictable ultrastructures and mesophase behavior linked to differering endosomal escape mechanisms. The observed pH-responsiveness correlates with protonation and altered aqueous solubility of protonated LAF-XP in the endosome (similar as has been noted for PEI and ionizable lipids). Interestingly, lipid phases of LAF-XPs do not change with pH (contrasting previously findings for standard LNPs). Instead, a pH-independent strong difference between sterically more flexible U-shapes (lamellar phase formation) and more rigid Bundles (fixation into non-lamellar bicontinuous phases with negative membrane curvature) was found. This ultrastructural difference is also reflected in transfection data. For productive transfection, U-shapes depend on endosomal protonation (like PEI-type polymers), whereas Bundles due to their pH-independent non-lamellar negative curvature lipidic properties are far less dependent on endosomal acidification. In sum, the LAF-XPs represent chemically linked dimers or tetramers of cone-shaped ionizable (LAF) lipids plus a polar head group. Our findings demonstrate that macromolecular topology, covalently caging these ionizable lipid units into defined spaces, plays a decisive role in dictating the overall ultrastructure and resulting properties, even when identical headgroups and similar ionizable lipid tail regions are employed.

## Results and discussion

### LAF-Stp XPs for mRNA polyplex formation

**Scheme 1:**
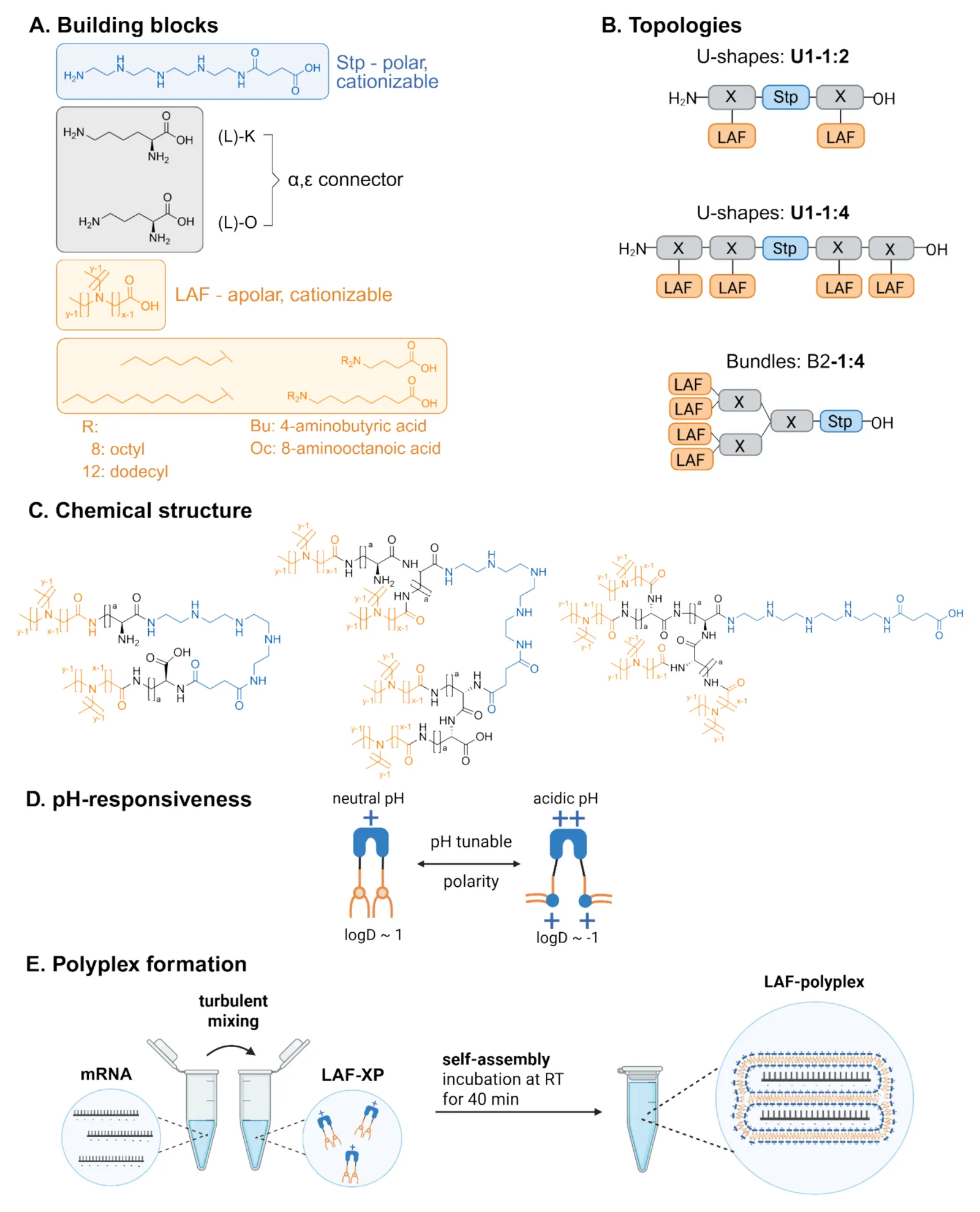
Design of the LAF-Stp XPs as carriers for mRNA delivery. **A** Building blocks for solid phase synthesis. Applied fmoc/tBoc protective groups are not displayed. Polar, cationizable Stp (blue) is connected with apolar, cationizable LAF units (orange) via *L*-lysine or *L*-ornithine branching linkers (grey) into **B** different topologies. **C** Chemical structures of U-shape (U1-1:2 and U1-1:4) and Bundle (B2-1:4) topologies. **D** pH-dependent polarity switch of LAF-Stp XP through protonation of apolar cationizable LAF domain at acidic pH. **E** Schematic illustration of LAF-Stp XP polyplex preparation. Stp, Succinoyl tetraethylene pentamine; L-(K), L-lysine; L-(O), L-ornithine; LAF, lipo amino fatty acid. Nomenclature of LAFs: The number (8, 12) relates to the number of C-atoms per terminal lipid tail, and the letters correlate with the ω-amino fatty acid (“Bu”, 4-aminobutyric acid; “Oc”, 8-aminooctanoic acid).

All lipo-xenopeptides (lipo-XPs) were synthesized by Fmoc solid-phase assisted peptide synthesis [32]. The polar, cationic headgroup consists of succinoyl tetraethylene pentamine (Stp) units, providing electrostatic nucleic acid complexation and endosomal buffering capacity. The lipophilic tail region contains at least two lipo amino fatty acid (LAF) domains (Scheme 1A) where an ionizable tertiary nitrogen is positioned within a branched hydrophobic alkyl chain. At neutral pH the nitrogen remains deprotonated, whereas endosomal acidification trig-gers protonation and a sharp polarity switch (~100-fold increase of water/octanol partition coefficient from pH 7.4 to pH 5.5, Scheme 1D) [32]. This double pH-responsive behavior allows LAF-Stp XPs to dynamically modulate solubility and membrane interactions, facilitating endosomal destabilization and subsequent cytosolic delivery. In general, LAFs were obtained by reductive bis-alkylation of ω-amino fatty acids with the corresponding fatty aldehydes. In the nomenclature, the number indicates the total number of carbon atoms within each lipid tail while the letters refer to the ω-amino fatty acid used. Hydrophilic and hydrophobic moieties were covalently linked through branched amino acids, initially L-lysine, to generate distinct XP topologies (Scheme 1B, C). Introduction of asymmetrical branching points lead to U-shaped XPs with LAFs attached to both the C-and N-termini. This configuration, combined with a lower LAF content (Stp:LAF = 1:2) allows them to adopt a flexible shape. In contrast, Bundles feature a dendritic and bulkier molecular architecture due to symmetrical branching and higher lipid content (Stp:LAF = 1:4). To bridge these structural differences, a third topology, U1-1:4, was introduced, maintaining the U-shape geometry while matching the lipid content of Bundles. Furthermore, the connector domain was modified by replacing lysine with ornithine, which preserved the overall topologies yet shortened the distance between headgroup and tail region by one methylene unit. This subtle modification may increase molecular rigidity and influence particle assembly and transfection efficacy. In total, eight LAF-XP were evaluated in this study. Each carrier was designated an individual 4-digit ID number and a unique code, denoting LAF and connector type, topology and Stp-to-LAF ratio (Table 1 and Table S1).

Polyplexes were formed by mixing equal volumes of LAF-Stp XP and mRNA dilutions, followed by 40 min of incubation at RT to allow self-assembly (Scheme 1F). Physicochemical nanoparticle characterization was conducted by dynamic (DLS) and electrophoretic (ELS) light scattering to determine size distribution, polydispersity and zeta potential at different molar nitrogen-to-phosphate (N/P) ratios (Table S2). For N/P calculations, all secondary, tertiary LAF-derived and primary N-terminal amines of the carriers were considered, regardless of their protonation state at neutral pH. U-shape carriers readily formed stable, positively charged polyplexes at the lower N/P ratio of 12, whereas Bundle XPs required higher N/P ratios to compact mRNA efficiently. Ornithine-containing Bundle carriers yielded larger or aggregated particles at low N/P ratios, albeit these differences diminished at higher ratios. Replacement of lysine with ornithine led to a slight increase in particle size and surface charge for U1-1:2 carriers. In contrast, within the U1-1:4 topology, the ornithine analogue yielded smaller particles with a lower yet sufficiently positive (13 to 18 mV) zeta potential. The encapsulation efficiency of mRNA polyplexes was further evaluated using a RiboGreen assay (Fig. S1) at N/P ratios of 18 for U-shape and 24 for Bundle LAF-Stp XPs. The results were consistent with DLS and ELS data, revealing marginally increased encapsulation efficiency for U-shapes compared to Bundle carriers. To assess the physicochemical stability and reproducibility of polyplexes, two representative U-shape (12Oc-K-U1-1:2, 1611) and Bundle XPs (8Oc-K-B2-1:4, 1621) along with their corresponding ornithine-analogues were formed into two independent polyplex batches and analyzed by DLS at day 0 and after 7 days of storage at 4 °C (Table S3). Sizes were stable over time, with U-shapes measuring ~87 to 113 nm and Bundle carriers ~95 to 108 nm. A slight increase in polydispersity (PDI) was observed after storage, particularly for Bundle XPs. Yet PDI values remained below 0.245, implying that the particles maintained relatively uniform. Intensity-size distribution plots supported these observations, showing consistent size distributions across all carriers and polyplex batches (Fig. S2).

### LAF-Stp XPs for mRNA transfection

The mRNA delivery efficacy of LAF-Stp XP containing polyplexes was evaluated in murine neuroblastoma (N2a), human cervix carcinoma (HeLa) andHeLa-Gal8-mRuby3 sensor cells. These cell lines were chosen as complementary and reproducible *in vitro* models to enable structure–activity relationship analyses across distinct cellular backgrounds. Relative light units (RLU) obtained for 12Oc-K-U1-1:2 (1611) were set to 1 for normalization and all other formulations were expressed relative to this benchmark (Table 2).

Transfection efficacy was further compared to succinylated polyethylenimine (succPEI) polyplexes and D-Lin-MC3-DMA (MC3) based lipid nanoparticles (LNPs). Across both cell lines, all LAF-Stp XP polyplexes outperformed succPEI. MC3-based LNPs achieved up to 3.8-fold higher luciferase expression than U-shape 1611 (Table 2, Fig. S3A,B). Among U-shape XPs, the ornithine containing 12Oc-O-U1-1:2 (1827) exhibited moderately enhanced efficacy relative to its lysine analogue 1611. Both U1-1:4 carriers were generally less efficient, with the ornithine-containing XP (1960) displaying the lowest luciferase expression. Bundle XPs consistently outperform U-shape LAF-XP polyplexes, with the most potent carriers 8Oc-O-B2-1:4 (1813) and 12Bu-K-B2-1:4 (1752) achieving polyplex transfection efficacies comparable to or exceeding those of MC3-based LNPs (Table 2, Fig. S3A,B). The relative metabolic activity of all polyplexes remained considerably high across all formulations (Fig. S3C,D) at the applied mRNA dose of 15 ng. This dose was selected based on prior dose–response analyses demonstrating that equal efficacy could be achieved while maintaining high metabolic activity. Hence, carrier performance was compared without confounding dose-induced cytotoxic effects. Furthermore, luciferase expression was evaluated for freshly prepared and stored U-shape and Bundle XP polyplexes (Fig. S4). After 7 days of storage at 4 °C, transfection efficacy did not decrease significantly for most carriers and no difference between distinct polyplex batches was detected (Table S4), indicating preserved activity and high reproducibility over the tested interval. Kinetic studies on cellular association, uptake and transfection efficacy were conducted in HeLa cells to further characterize topology-dependent effects (Fig. S5). Two U-shape (U1-1:2) and Bundle XPs (B2-1:4) were selected and analyzed via flow cytometry at distinct time points over 1–48 h. Polyplexes containing Cy5-labeled mRNA showed rapid cellular association, with up to 60% Cy5-positive cells after 1 h and 80% after 2 h for B2-1:4 carriers. In contrast, U-shape XPs exhibited slower uptake kinetics, reaching ~30% and ~60% at the respective time points (Fig. S5A). These findings strongly corroborated the expression of GFP. While Bundle carriers achieved up to 80% GFP-positive cells after 2 h and 100% after 4 h, U1-1:2 reached only ~20% and at least 80% after 4 h (Fig. S5B). Given the pronounced topology-dependent differences, we next sought to elucidate whether these effects were linked to distinct endosomal escape mechanisms. Endosomal acidification represents a key factor in facilitating endosomal escape and correspondingly efficient nucleic acid delivery. To examine pH-dependency, cells were transfected in the presence of bafilomycin A1, an inhibitor of the endosomal vacuolar ATPase. While both topologies demonstrated reduced luciferase expression (Table 2, Fig. S6), Bundle XPs were far less affected (1.6- to 4.5-fold) than U-shape carriers (8- to 23-fold), implying distinct endosomal escape mechanisms for both topologies. To exclude reduced cellular uptake as the primary cause of decreased efficacy in the presence of bafilomycin, uptake of Cy5-labeled polyplexes was analyzed in N2a cells after 2 h of incubation (Fig. S7). While U1-1:2 XPs generally exhibited lower uptake than U1-1:4 and B2-1:4 carriers, bafilomycin A1 did not significantly alter the number of Cy5-positive cells, indicating that impaired endosomal acidification rather than cellular uptake accounts for the observed drop in luciferase expression. Further mechanistic studies on endosomal destabilization were assessed via Galectin8-mRuby3 imaging by confocal laser scanning microscopy (CLSM; Fig. S8) in HeLa-Gal8-mRuby3 cells. Upon endosomal membrane rupture, Gal8 interacts with exposed glycans on the inner endosomal leaflet, inducing green, punctuate fluorescence. Distinct fluorescence patterns were found for both topologies. For the U1-1:2 topology, small, diffuse puncta were observed, whereas Bundle XPs generated large, bright spots, indicative of pronounced endosomolytic activity (Fig. S8A,B). Consistent with qualitative CLSM observations and kinetic studies, quantification of Gal8 recruitment by counting puncta per cell revealed higher values for Bundle XPs (Fig. S8C). Spearman correlation analysis (Fig. S8D,E) revealed a positive association between Gal8 puncta and the fraction of GFP-positive cells after 4 h (*ρ* = 0.8, *p* = 0.333), as well as a negative association with relative metabolic activity (*ρ* = −0.8, *p* = 0.333). Although statistical significance was not reached due to the limited number of conditions analyzed, these trends support a topology-dependent relationship between endosomal membrane destabilization, transfection efficacy and metabolic activity.

### Molecular dynamics (MD) simulations of LAF-Stp XPs at hydrophilic-lipophilic interfaces

**Fig. 1:**
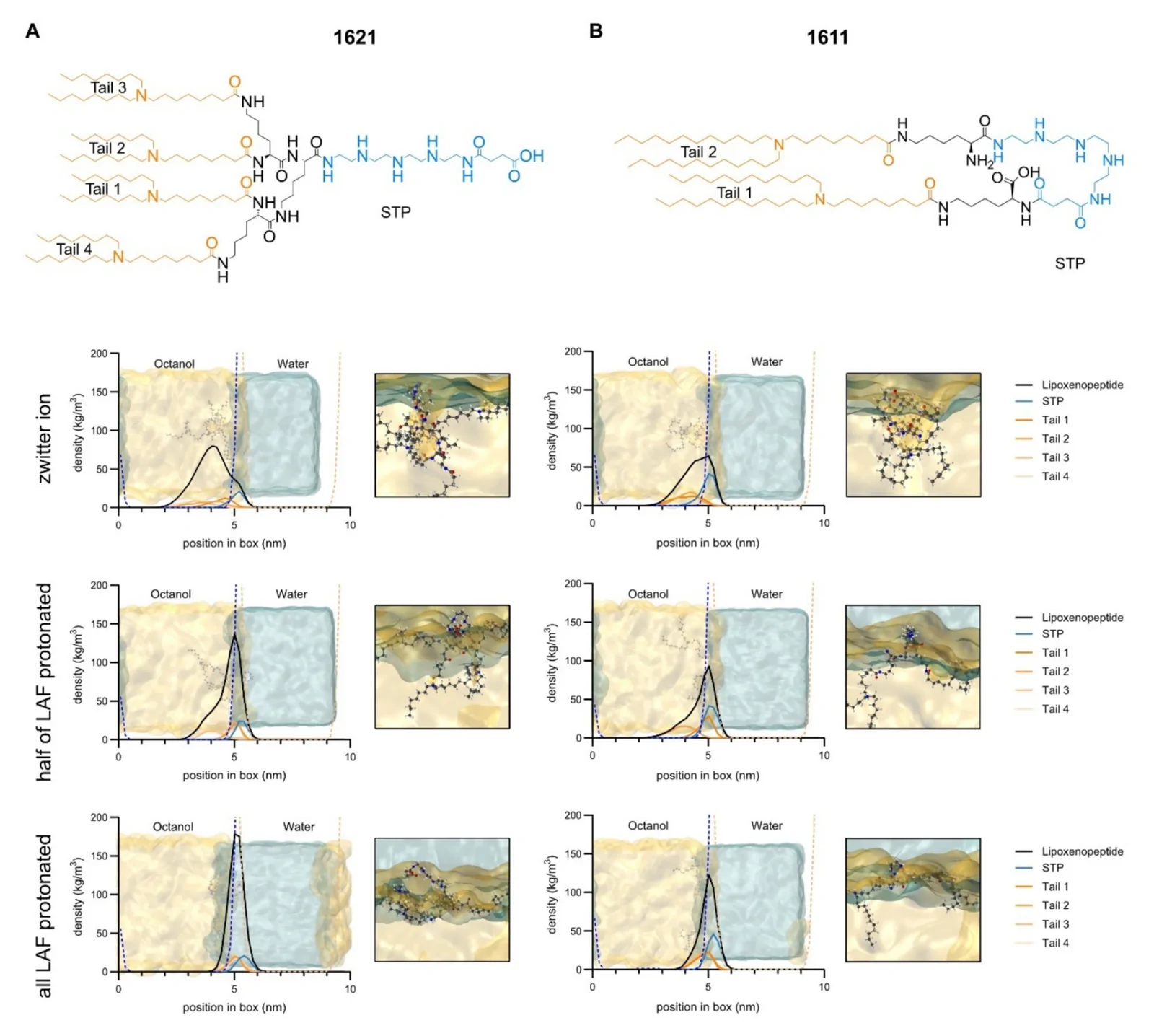
MD simulation of differently charged carriers at the water–octanol (W/O) interface. **A** Molecular structures of the Bundle XP 8Oc-K-B2-1:4 (1621) and U-shape carrier 12Oc-K-U1-1:2(1611) carriers; hydrophobic LAF tails are shown in orange and the polar Stp segments in blue. **B** Density distributions of the carriers along the water–octanol interface, with simulation snapshots from the final frame shown in the background (yellow: octanol; blue: water). Insets provide zoomed-in views of interfacial organization. (n = 3, mean).

Given the experimentally observed amphiphilic behavior of LAF-Stp XPs in previous work and the current study (Table S5), MD simulations were conducted to visualize their interfacial organization and self-assembly behavior at the molecular scale. Hence, two representative U-shape (12Oc-K-U1-1:2) and Bundle XPs (8Oc-K-B2-1:4) were simulated at the water-octanol (W/O) interface (Fig. 1) at different protonation states. This simulation setup was selected to validate parametrization of the carriers and have a direct comparison between *in silico* work and experimental values (Table S3). Its primary purpose was to confirm that the distinct differences in log D values between Bundle and U-shape topology in log D values are captured with the chosen parametrization of the lipo-XPs. Lipid tails were protonated sequentially, beginning with tail 1 and continuing stepwise. For clarity, only the half- and fully charged states are presented. The simulations were consistent with empirical logD (octanol/water partition coefficient) measurements, where U-shapes exhibited higher polarity at neutral pH than Bundle XPs. Accordingly, the zwitterionic 12Oc-U1-1:2 carrier (1611) demonstrated pronounced localization at the water-octanol interface compared to Bundle XP 8Oc-B2-1:4 (1621). Density distributions revealed increased accumulation of both topologies at the interface upon further protonation. Notably, uncharged LAFs persisted in the octanol phase of the simulation box, visible as a shoulder in the black graph or as orange peaks not yet shifted toward the aqueous region. In alignment with logD results, U-shapes, due to their intrinsically higher polarity, displayed a less pronounced migration toward the interface than Bundle carriers. Moreover, the lipid tails of 12Oc-K-U1-1:2 (1611) demonstrated less relocation into the interface compared to 8Oc-K-B2-1:4, indicated by shoulders in the tail region and whole carrier distribution (black).

To evaluate whether trends observed at the W/O interface translate to endosomal membrane behavior, both carriers were simulated in a DOPC/cholesterol (70/30 lipids per leaflet) bilayer for 100 ns. Carrier behavior was analyzed using density distribution of headgroups and LAF tails (Fig. S9) and radial distribution functions (RDFs; Fig. S10) and compared with W/O interface data. Overall, the trends closely matched those observed at the W/O interface. With increasing protonation, both carriers shifted toward the aqueous phase and out of the membrane. Bundle XPs, which are larger and exhibited stronger visually observed membrane-disruptive behavior (Fig. S9B), showed a more pronounced displacement than U-shaped XPs (Fig. S9A,C), consistent with W/O interface results (Fig. 1). In general, the RDF describes a molecule’s tendency to interact with the surrounding solvent at a specific distance (r (nm) = distance between water and molecular groups; g(r) = radial distribution relative probability of find atoms at distance r, compared to a completely random distribution). Upon protonation, LAF tails increasingly relocated toward the aqueous phase (Fig. S9, S10). RDF analysis revealed only a minor deviation for U-shape Stp headgroups, with a slight shift of the first peak to larger distances, while no major differences were observed, particularly for Bundle topologies, between membrane and W/O simulations. The strong qualitative agreement between membrane and W/O interface simulations mirrors experimental findings, where Bundle XPs display higher membrane-disruptive activity than U-shaped XPs upon protonation. Together, these results support the W/O interface as a rapid and effective *in silico* model for capturing key mechanistic trends and enabling efficient screening.

### MD simulations of LAF-Stp XP self-assembly

**Fig. 2:**
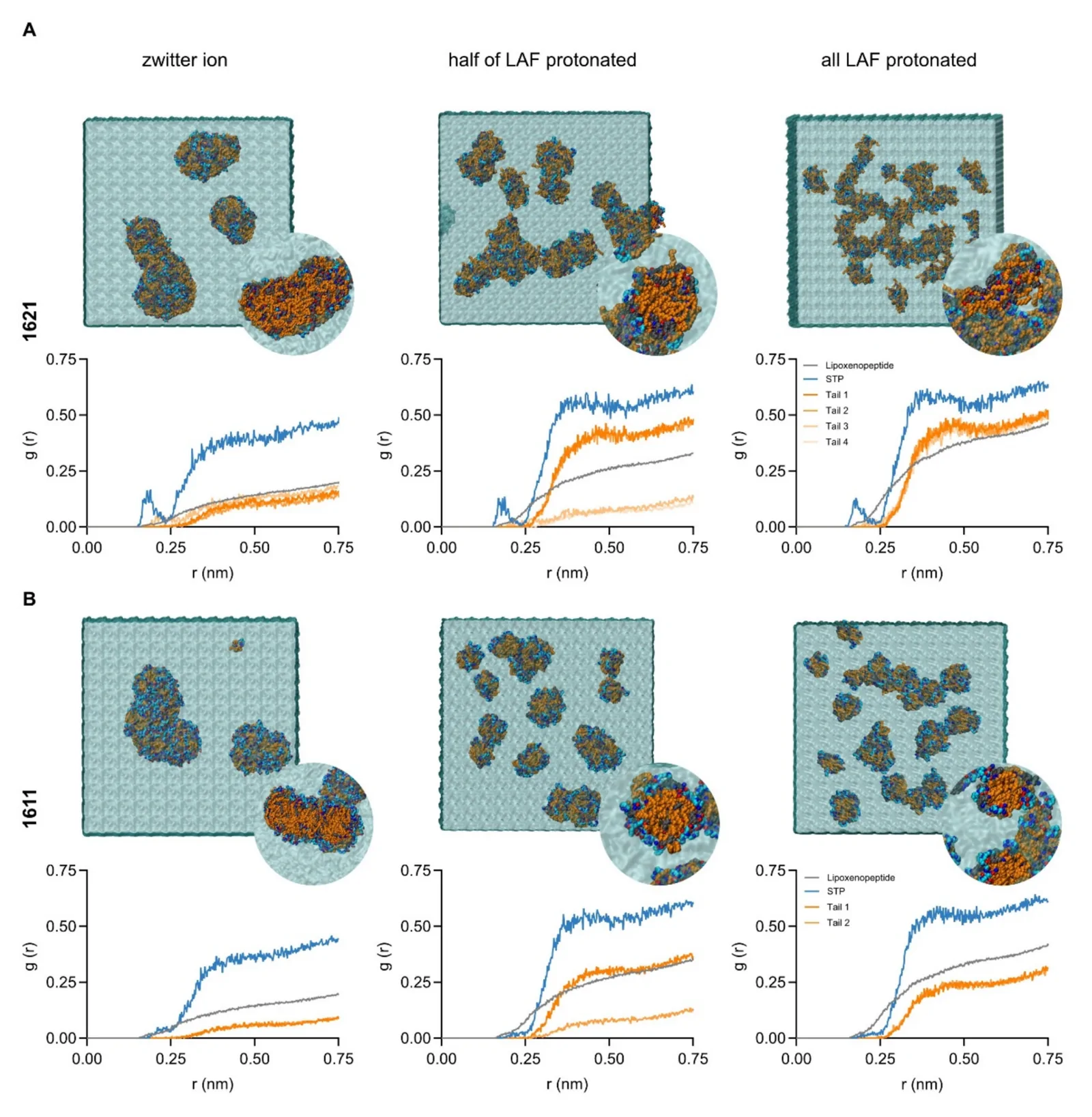
Self-assembly MD simulations of differently charged carriers in 0.9% NaCl solution. **A** Simulation snapshots of the Bundle carrier 1621 (8Oc), including a zoomed-in cross section of an individual particle along the box axis. Below, radial distribution functions (RDFs) from the final simulation frame are shown, analyzing the distribution of the carrier backbone (STP) and lipid tails relative to water. **B** Corresponding snapshots and RDFs for the U-shaped carrier 1611 (12Oc). (*n* = 1). In all representations, lipid tails are shown in orange, STP backbones in cyan, and linker atoms in gray; hydrogens and ions are omitted for clarity.

In analogy to previously published polyplex [70] and LNP self-assembly studies [71,72], all-atom (AA) self-assembly MD simulations were performed to investigate self-assembled structures at different protonation states (Fig. 2). Carriers were simulated in 0.9% NaCl for 140 ns to probe early to intermediate cluster formation and compare trends across protonation states (Fig. S11). At the zwitterionic state, the number of clusters was minimal, whereas half and fully protonated lipo-XPs formed a greater number of clusters. Combining these observations with W/O interface density distributions (Fig. 1), both carrier topologies showed distinct interfacial behaviors explaining their clustering tendencies. Bundle XPs formed elongated structures with a smaller diameter spanning the water box and exhibited pronounced redistri-bution into the aqueous layer upon protonation (Fig. 2A), reflecting stronger electrostatic interactions associated with higher LAF density. In contrast, U-shaped XPs predominantly assembled into smaller clusters, in accordance with their behavior at the W/O interface. Upon protonation they largely remained embedded within the interface without major displacement toward the aqueous phase (Fig. 2B), likely due to weaker electrostatic interactions resulting from their lower LAF content. Both topologies assemble into micellar structures, with 8Oc-B2-1:4 (1621) generating more clusters (Fig. S11). Cross sections of the zwitterionic states show clear micellar organization for Bundle (Fig. 2A) and U-shape XPs (Fig. 2B). Protonation led bundle carriers to adopt extended, tunnel-like structures spanning the entire simulation box, with less defined internal organization. In contrast, U-shapes tend to form compact, rounded assemblies. Radial distribution functions (RDFs) towards water were analyzed for the carrier, Stp domain and respective lipid tails at distinct charge states (Fig. S12). For 8Oc-B2-1:4 (1621) (Fig. 2A), increasing protonation of the Stp domain promoted migration toward water, with higher abundance observed in the first solvation shell (increase beyond 0.25 nm for half- and fully protonated state). The LAF units follow a similar trend. Uncharged tails remain distant from water. Upon half-protonation, two tails demonstrate RDF profiles comparable to the Stp group. At the fully charged state, all four tails align with the Stp curve, correspondingly resulting in a markedly increased solvation of the entire carrier. For 12Oc-U1-1:2 (1611) (Fig. 2B), the Stp domain exhibited similar trends, though interactions <0.25 nm, indicative of hydrogen bonding, were less pronounced. At zwitterionic and half-protonated state, the LAF domains resemble the behavior of 1621, shifting closer to water upon protonation. Notably, LAFs in fully charged state relocate to an intermediate position between zwitterionic and half-protonated profiles, rather than fully aligning with the Stp curve. These differences may be topology-dependent, as the C-terminal Stp headgroup of 8Oc-B2-1:4 could move more freely than the centered Stp moiety of 12Oc-U1-1:2 (1611). Interestingly, self-assembly simulations reflected trends observed at the water-octanol interface (Fig. S10). Thus, W/O interface simulations may predict self-assembly behavior, correspondingly providing a less computationally intensive proxy. In addition, RDFs are confirmed by visual inspection of the trajectories, showing carriers transitioning from tightly packed clusters with lipid tails tucked inside to more open structures. Upon full protonation, U-shape carrier 1611 remains more compact compared to Bundle carrier 1621, hence revealing clear topological differences.

### Analysis of LAF-XP self-assembly by SAXS

**Fig. 3:**
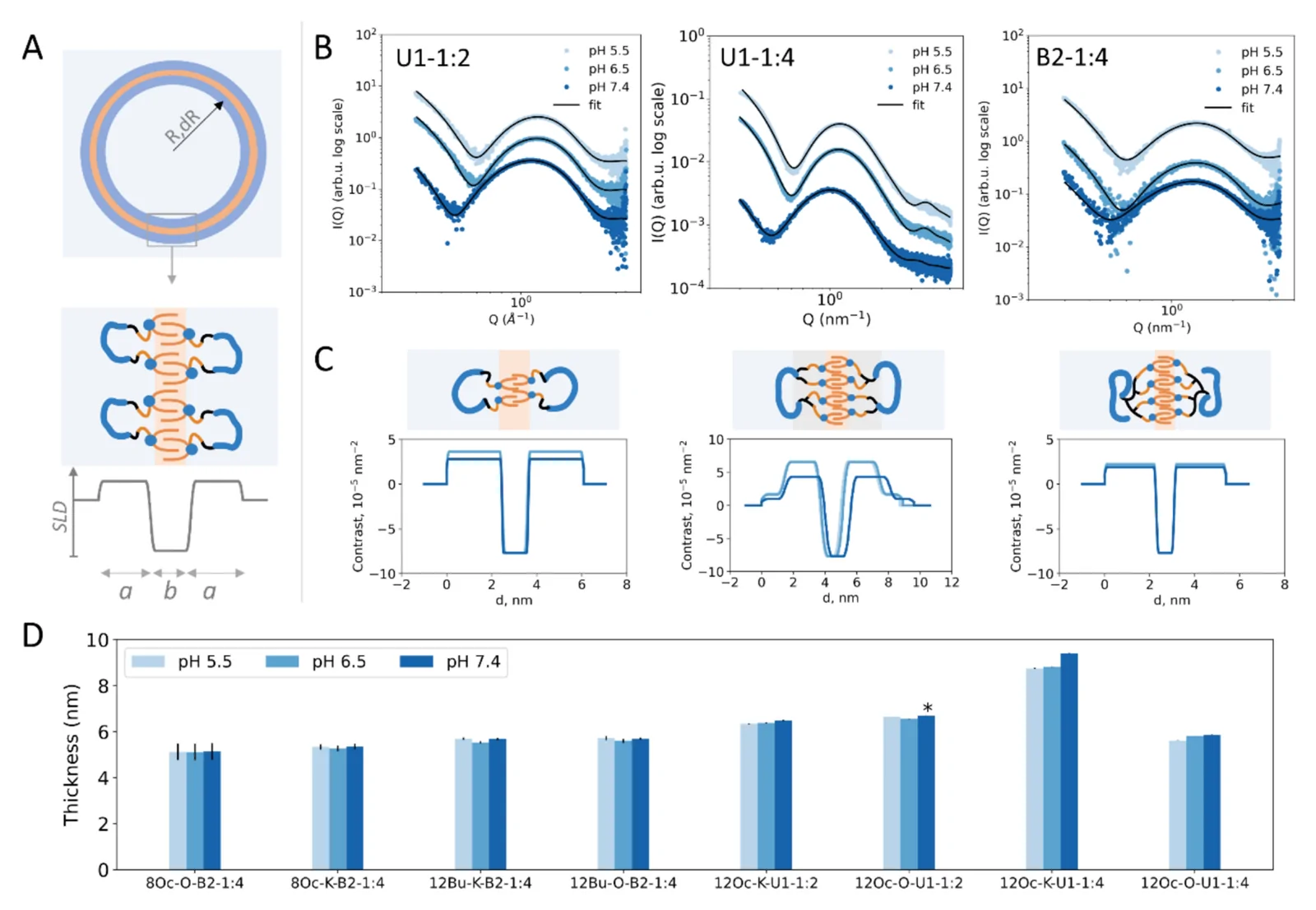
Self-assembly SAXS measurements of LAF-Stp XPs of different topologies. **A:** Model large unilamellar vesicle (LUV) made of a Bundle carrier and a representative scattering length density (SLD) contrast profile described with a three-layer model. **B**: pH-dependent SAXS data for three representative carriers of each topology: 12Oc-K-U1-1:2 (1611),12Oc-K-U1-1:4 (1718) and 8Oc-K-B2-1:4 (1621) and the LUV model fits. **C**: Contrast profiles extracted from the fit results. The color-code is the same as in B. D: Bilayer thicknesses extracted from the fits with LUV model for all measured carriers. Star indicates that the lamellar spacing instead of the bilayer spacing is presented.

The distinct topological configurations and amphiphilic character of the lipo-XPs potentially lead to a complex self-assembly behavior, as indicated by MD simulations and further examined by SAXS. Accordingly, lipo-XPs were rehydrated in aqueous buffers (2 wt%) mimicking physiological, early and late endosomal pH environments. All carriers formed stable, slightly turbid dispersions when dispersed in buffer implying the presence of sub-micron sized particles. DLS measurements confirmed particle sizes in the range of 50–300 nm, depending on the respective carrier and pH (Table S6). SAXS analysis revealed the formation of large unilamellar vesicles (LUVs) for all XPs (Fig. 3A–C).The data was fitted using a LUV model [73] in which the bilayer was modeled by three layers for the U1-1:2 and B2-1:4 topologies and five layers for U1-1:4. For all carriers the inner hydrophobic region appeared notably thin (≈ 1 nm), indicating that water penetrates deeply into the bilayer even in the non-ionized state. Acidification of the buffer had only a minor impact on the bilayer structure and did not alter the overall carrier organization. Protonation of the carriers, however, attracted more water into the bilayers headgroups reflected by a decrease in the headgroup scattering length density (SLD). A visible increase in the overall bilayer thickness was observed only for the U1-1:4 topology (Fig. 3D).

Interestingly, 12Oc-K-U1-1:4 (1718) XP required two additional layers in the fitting model. The most outer layers displayed an SLD rather close to water, indicating that they contained only a small fraction of Stp headgroups. Despite equal lipid content to the U1-1:4 topology Bundle XPs formed a bilayer comparable to that of U1-1:2, elucidating distinct incorporation modes of Stp units in U-shape and Bundle XPs. Next, the influence of structural modifications such as linker length and the position of the ionizable nitrogen within the lipid tails on the bilayer architecture was investigated. The linker length had a pronounced effect on U-shape XPs. In the U1-1:2 topology, shortening the linker from lysine (K) to ornithine (O) induced the formation of a multilamellar phase at neutral pH, with a lamellar spacing similar to the bilayer thickness at acidic pH, suggesting a water-free or a bola-like lamellar arrangement (Fig. S13A,C). The U1-1:4 topology also showed a bilayer structure depending on the type of linker. The longer lysine linker yielded a thicker bilayer comprising at least five distinct layers. In contrast, the shorter ornithine led to a bilayer organization comparable to that of other carriers and could be fitted with the classical three-layer model (Fig. S13B,D). For Bundle XPs, shifting the position of the ionizable nitrogen within the LAF domain was found to promote a slightly thicker bilayer. The repositioned tertiary nitrogen moves closer to the hydrophilic Stp headgroup [39] and may effectively become part of it, which could increase headgroup hydration and thereby contributing to the thicker bilayer.

### LAF-XP self-assembly data: comparison of MD simulation and SAXS

The amphiphilic character of LAF-Stp XPs governs their tendency to self-organize in aqueous environments. The dramatic shift in polarity upon transition from extracellular pH 7.4 to endosomal pH 5.5, well documented by an about two log unit change in the octanol/water partition coefficient (logD) (see Table S5 and [32]), is consistent with MD simulations at an octanol/water interface as presented in the current study (Fig. 1). Protonation of the tertiary amines within the LAF units and Stp headgroup explain the strongly increased hydrophilicity. Both MD simulations (Fig. 2) and SAXS measurements (Fig. 3) confirmed the formation of high-ordered structures of LAF XPs in aqueous environment, albeit distinct types of assemblies were obtained. In the simulations, the representative U-shape (12Oc-K-U1-1:2, 1611) and Bundle XP (8Oc-K-B2-1:4, 1621) predominantly formed elongated micelles and, depending on the protonation state, elongated smaller tube-like clusters for 1621. In contrast, SAXS measurements revealed the formation of LUV featuring bilayers for all topologies. These differences may arise from the distinct methodological frameworks. To capture structural rearrangements without losing the atomistic detail provided by the simulations, we retained an all-atom (AA) simulation scale. While LUV formation with phospholipids can be modeled by molecular dynamics, it is typically performed using lower-resolution, coarse-grained approaches [70]. An additional distinction lies in chemical resolution. Whereas MD simulations require fixed protonation states that are modeled individually, each SAXS measurement reflects an ensemble average over multiple protonation equilibria at a given pH. Accordingly, a limitation of the present AA-MD setup is that protonation states were assigned a priori and kept fixed throughout the simulations, which may affect absolute partitioning and long-time self-assembly behavior. Nevertheless, protonation states are evaluated qualitatively with W/O interface simulations (Fig. 1) and logD measurements (Table S5), and the direction of the simulated trends across protonation states is consistent with experimentally reported logD trends (Table S5), supporting that the chosen protonation scenarios capture the dominant qualitative changes in hydrophilicity and hydrophobicity. The accessible time scale and sampling depth of the AA-MD simulations are also limited (140 ns for self-assembly simulations, n = 1). Consequently, conclusions regarding final ultrastructure and detailed morphology should be interpreted qualitatively, whereas trends in local solvation and headgroup–tail water interactions are considered more robust. Although imaging artifacts were minimized by reconstructing whole trajectories prior to cluster analysis, large-scale morphologies may still be influenced by finite box size and periodic boundary conditions. In contrast, RDF analyses were restricted to short distances (0–0.75 nm), well below half the box length, where periodic boundary effects are negligible. Despite these limitations, self-assembly simulations clearly differentiate U-shaped and Bundle topologies, demonstrating, even at intermediate equilibrium, that carrier topology and protonation degree govern structural organization. This shared observation supports the conception that the self-assembly of LAF-Stp XPs is driven by amphiphilicity. Protonation was found to enhance solvation without inducing complete structural rearrangement. Investigating the SAXS data in detail reveals how topology and linker chemistry alter the hydration behavior. Among U-shapes, the U1-1:2 topology exhibited the highest sensitivity towards linker modifications. Shortening the linker from lysine to ornithine shrinks the distance between polar headgroup and lipophilic tail region, increases molecular rigidity and promotes the formation of multilamellar arrangements at neutral pH. In contrast, the more LAF-rich U1-1:4 carriers maintained lamellar organization, although the bilayer-thickness depended on the type of linker. While lysine allowed the formation of more hydrated, thicker bilayer (five-layer fits), the shorter ornithine resulted in a denser bilayer (three-layer-fit). Bundles, despite their dendritic geometry and high lipid content, formed bilayers comparable to the U1-1:2 topology. These findings suggest that their symmetric branching may localize hydrophobic tails and polar head-groups on opposing sides of the assemblies, thereby inducing lamellar order. This convergence emphasizes that the spatial incorporation of Stp units could dominate the supramolecular organization. Furthermore, these supramolecular structures may play a role during nanoparticle formation. The presence of the LUV domains implies that the carriers are able to pre-organize into defined, amphiphilic assemblies even prior to complexation with nucleic acids. Such pre-structuring is known to modulate physicochemical determinants and corresponding properties of nanoparticle formation along with enhanced transfection efficacy [43,74–77].

### Analysis of LAF-XP/cholesterol lipid bulk phases by SAXS

Following the supramolecular characterization of the LAF-Stp XPs by MD simulations and SAXS, their self-assembly behavior under condensed lipidic conditions was investigated, as the equilibrium phases in such states can differ from the aqueous bilayer structures observed previously. In polyplexes, these XPs are expected to form tightly packed molecular arrangements driven by electrostatic interactions with mRNA. Studying such phases within nanoparticle formulations by SAXS is challenging due to their high structural disorder. Moreover, LAF-XP molecules on the surface or released from polyplexes upon endosomal solubilization are considered to be in direct contact with cellular membranes. Therefore, we focused on the interaction of LAF XPs with cholesterol assemblies as simplified mimics of cellular endosomal membranes. Cholesterol instead of cholesterol/phospholipid mixtures were used in these first studies, to avoid complications by electrostatic interactions with phospholipids. For this purpose, lipid bulk phases containing LAF XPs and cholesterol were employed to stabilize the condensed assemblies and reduce structural disorder, thereby enabling high-resolution SAXS analysis as in previous studies [66]. Bulk samples were prepared by rapid mixing of LAF-Stp XP/cholesterol ethanolic solutions with acidic buffer, followed by a few dialysis steps ending with a desired buffer of a defined pH. These systems served as models to study structural transitions of condensed carriers under distinct pH conditions.

**Fig. 4:**
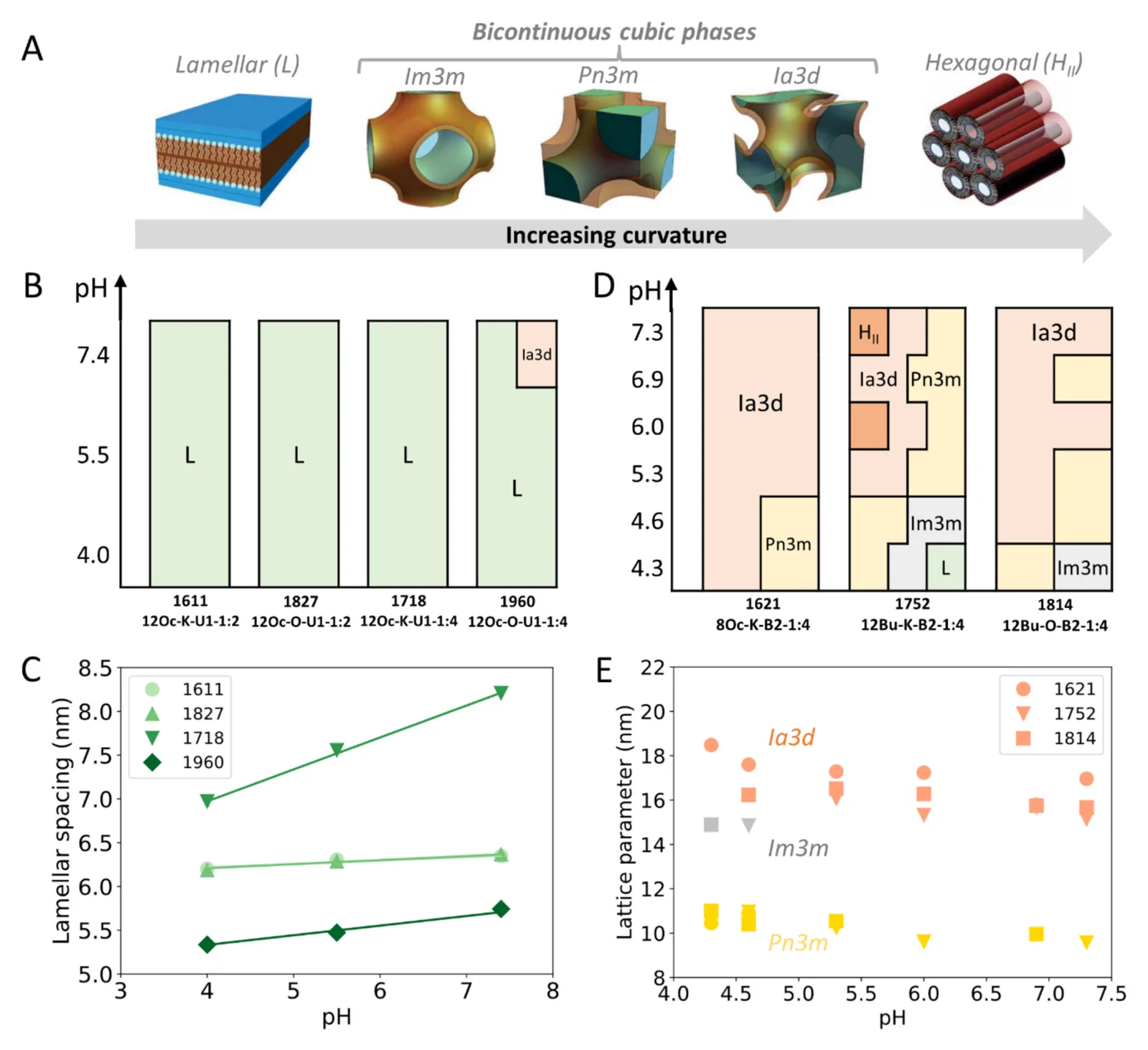
SAXS study of lipo-xenopeptide/cholesterol bulk phases formed with different carriers. A Schematic representation of the observed phases sorted by their mean curvature. Reproduced from Aleandri, S. and Mezzenga, R., The physics of lipidic mesophase delivery systems, Physics Today, 73(7), 2020, https://doi.org/10.1063/PT.3.4522; Fig. 1 with the permission of the American Institute of Physics. B Phase diagram for the U-shaped carries as a function of pH. C Lamellar spacing of the U-shaped carriers as a function of pH. D Phase diagram for the Bundle-shaped carries as a function of pH. E Lattice parameter of the Bundle-shaped carriers as a function of pH.

In bulk phases, U-shape XPs predominantly formed lamellar phases, with the exception of 12Oc-O-U1-1:4, which transitioned into a bicontinuous cubic Ia3d phase at pH 7.4 (Fig. 4B, Fig. S14D). Otherwise, variations in the buffer pH affected only the lamellar spacing, which correlated closely with the bilayer thickness of the corresponding LUVs (Fig. 4C). The lamellar spacing of the more lipid rich U1-1:4 topology was highly pH responsive and nearly identical to the bilayer thickness of LUVs observed above, indicating the absence of an interlamellar water layer. Consistent with LUVs, the linker type induced a dramatic change of the lamellar spacing. Interestingly, the flexible U1-1:2 XPs exhibited almost no pH responsiveness or dependence on the linker and displayed lamellar repeat distances equal to the bilayer thickness of LUVs. In contrast, Bundle XPs demonstrated a very rich pH-dependent phase behavior including lamellar, three different bicontinuous cubic and inverted hexagonal phases (Figs. 4A, C, Figs. S14–16). Upon acidification, ionization of the carriers triggered phase transitions to structures of lower mean curvature (Fig. 4A). This behavior resembles that of classical ionizable lipids like MC3 or ALC-315 [66,67], with the key distinction that LAF-Stp XPs already form inverted hexagonal and bicontinuous phases at high pH, whereas conventional ionizable lipids undergo these transitions at pH 5–6 in the presence as well as absence of negatively charged phospholipids. Notably, Bundle XPs form lipid phases of negative curvature with cholesterol in the absence of phospholipids. Both the linker length and position of the ionizable nitrogen within the tail region modulated the pH-responsiveness of the mesophases. Shifting the nitrogen closer to the headgroup and extending the linker increased the mean curvature of the mesophases and the pH-sensitivity of the carrier, yielding the richest phase behavior for 12Bu-K-B2-1:4 (1752). The nitrogen position, however, appeared to have a stronger impact than linker length, as 8Oc-K-B2-1:4 (1621) showed minimal pH responsiveness and persisted in a single phase across the endosomal pH range from 7.4 to 5, favoring the largest mean curvature. All bicontinuous phases expanded under acidic pH, indicating that the phase curvature decreases continuously during ionization (Fig. 4E). To enhance the biomimetic relevance of lipid bulk phases with respect to endosomal membrane environments, DOPC was incorporated together with a representative U-shape (12Oc-K-U1-1:2, 1611) or Bundle XP (8Oc-K-B2-1:4, 1621), and cholesterol. For 12Oc-K-U1-1:2 (1611), no pronounced formation of a second lamellar phase was detected, indicating high miscibility with DOPC across the investigated pH range (Fig. S20A). In contrast, for 8Oc-K-B2-1:4 (1621) a coexistence between the Bundle XP-derived Hll phase and the lamellar phase of DOPC was observed at pH 7.3 (Fig. S20B). Upon decreasing the pH to 5.4, the H_ll_ phase was no longer present, and the scattering profiles were dominated by a lamellar phase coexisting with lipid vesicles. Although SAXS does not resolve the location of the LAF-Stp XP within the lipid assemblies, the observed phase behavior indicates that the XP adopts a lamellar organization either within the multilayers, vesicular structures, or both, suggesting that it mixed with DOPC.

### Relationship of LAF-XP ultrastructure and bioactivity

**Scheme 2:**
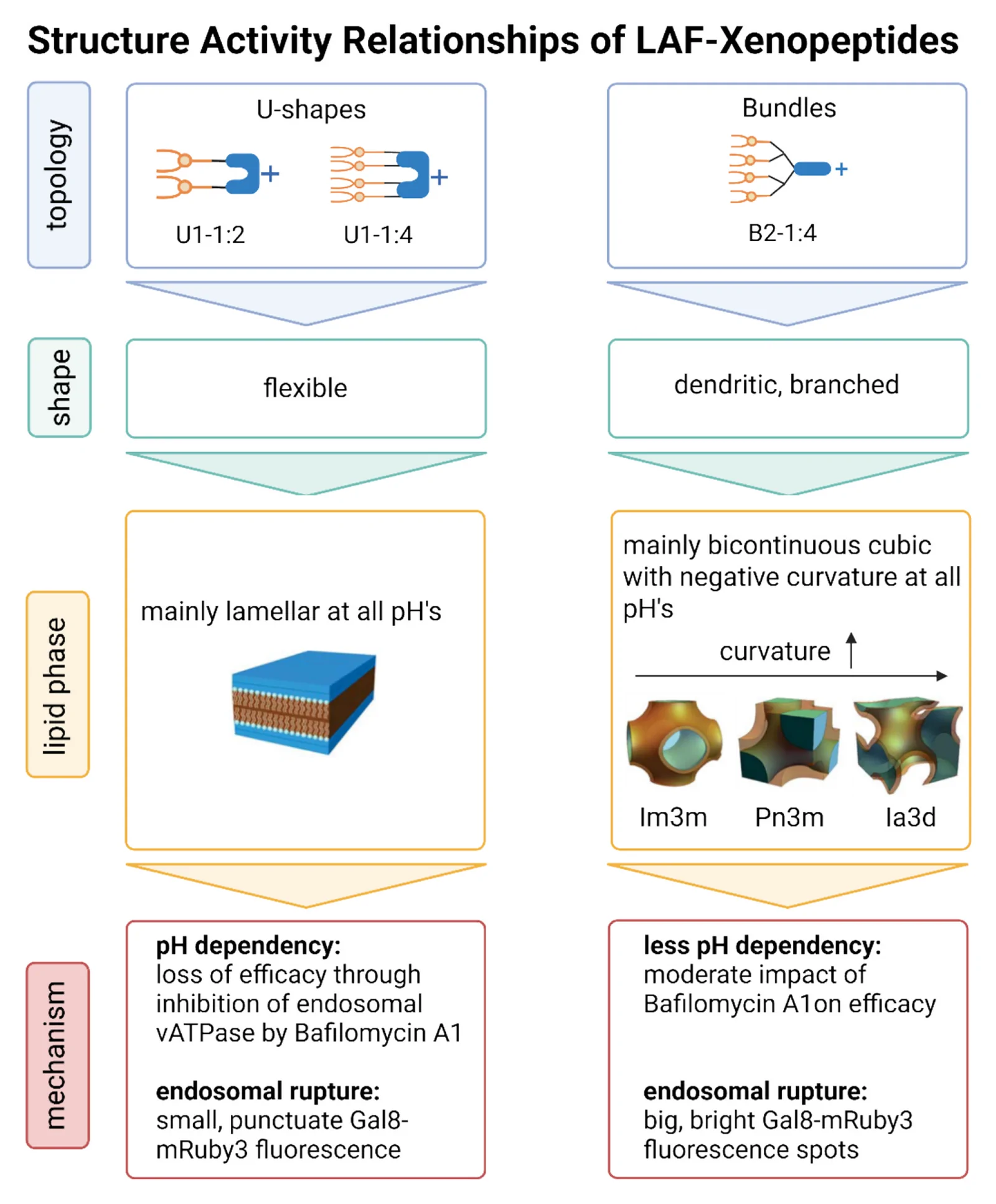
Comparison of distinct structure–activity relationships between U-shape and Bundle LAF-Stp XPs. Images of the lipid mesophases, also presented in the graphical abstract were reproduced from Aleandri, S. and Mezzenga, R., The physics of lipidic mesophase delivery systems, Physics Today, 73(7), 2020, https://doi.org/10.1063/PT.3.4522; Fig. 1 with the permission of the American Institute of Physics.

The LAF-Stp XP topology, chemical constitution and ratio of lipophilic/hydrophilic domain (LAF/Stp) define the resulting lipid-phase organization and potentially underlying endosomal escape mechanisms. Even subtle molecular variations, including the position of the ionizable nitrogen within the LAF domain [32,39], polarity of the headgroup [78,79], or the distance between headgroup and tail region [80], modulate functional responsiveness. For example, spacers inserted between LAF and Stp domains altered the mRNA transfection efficiencies and kinetics of expression [80]. Such previous findings were largely empirical. The current information obtained by SAXS analyses and MD simulations enables for the first time integration and correlation of ultrastructural information on LAF-XPs with their performance in biological processes such as endosomal escape and mRNA transfection (Scheme 2).

For U-shape carriers, both the U1-1:2 and U1-1:4 topology maintains predominantly lamellar arrangement across the entire pH range, displaying no transition toward more highly curved structures such as bicontinuous cubic or inverted hexagonal phases (Fig. 4B). These findings were further supported by transmission electron microscopy (TEM) analysis of mRNA polyplexes containing 12Oc-K-U1-1:2 (1611, Fig. S18). At physiological pH, TEM images revealed globular nano-particles with a multilamellar, onion-shape like internal organization. The mean diameter was approximately 42 nm and thus smaller than the corresponding hydrodynamic diameter obtained for DLS. The discrepancy may reflect the high sensitivity of DLS to minor aggregate fractions. Consistent with the lipid bulk phases, the lamellar organization was preserved upon acidification. However, the particles appeared more elongated, accompanied by increased mean diameters and increased polydispersity, implying structural arrangements within the polyplex in an acidic environment (Fig. S18B). Consequently, endosomal escape mechanisms based on lipid curvature-related membrane destabilization as postulated for LNPs with a pH-triggered hexagonal H_II_ phase formation [55,57] are unlikely to be involved. A more realistic mechanism is based on a protonation triggered effect similar as hypothesized for cationizable polymers such as PEI [43,48,50,53]. Upon acidification, protonation of both the Stp headgroups and tertiary amines within the LAF domains alters intralamellar electrostatics and aqueous solubility (see large changes in logD [32], and ultrastructural parameters Figs. 1–3), leading to exposure of positive charges at the surface. This could facilitate direct electrostatic interaction with endosomal membranes and transient pore formation without disrupting the overall lamellar structure. In addition, partial molecular disassembly may release individual XP molecules capable of integrating into the endosomal membrane and locally perturbing its integrity. Both phenomena could act synergistically, resulting in localized, reversible membrane permeabilization, a behavior more commonly associated with lipoplex-based delivery systems [46,81,82]. This hypothesis is consistent with two separate biological findings; inhibition of endosomal acidification via vATPase inhibitor bafilomycin A1 strongly (up to >20-fold) reduces mRNA delivery efficacy of U-shapes (see Table 1), indicating the dependence on pH-triggered escape mechanisms, similar to those found for polycations such as PEI [43,50,51,53]. This hypothesis is also supported by confocal imaging of galectin-8-mRuby3 fusion protein fluorescence (Fig. S8C), which revealed small, dispersed fluorescent puncta characteristic of localized membrane perturbation rather than massive rupture events as observed for Bundle XPs [32]. Modulation of distinct molecular features may impact the proposed delivery mechanism. Parallel studies on xenopeptides replacing Stp with hydrophobically refined headgroups report improved delivery efficacies for selected U-shape XPs as compared to Stp-based analogues [78,79]. Altered headgroup polarity may support lipid-based membrane interactions and enhance endosomal escape.

In comparison to U-shapes, Bundle XPs (B2-1:4) display a fundamentally different ultrastructural organization in lipid bulk phase and differing endosomal escape profile. Their dendritic, highly branched and symmetric architecture promoted the formation of bicontinuous cubic and inverted hexagonal phases already at neutral pH. These phases are characterized by a continuous, three-dimensionally interconnected lipid matrix and aqueous channels, which are considered highly fusogenic and have been directly associated with efficient endosomal cargo release in lipid nanoparticle systems. In these studies, cubic phases were shown to facilitate lipid mixing and transient pore formation at the endosomal membrane, effectively bridging the gap between stability and fusogenicity [60,83]. The observation of such phases for Bundle XPs therefore indicates that their architecture inherently predisposes them to adopt curvature-frustrated, fusion-competent geometries [84,85], providing a structural rationale for their high and fast transfection efficacy. Notably, TEM images show polyplexes with isotropic contrast patterns, consistent with a non-lamellar, potentially bicontinuous internal organization (Fig. S18C,D). Upon acidification, similar to conventional ionizable lipids like MC-3 or ALC-315 [64,66,67,86], their mean curvature further decreased, most likely due to electrostatic repulsion, particularly between the protonated LAF-nitrogens and the Stp headgroup. In addition, the position of the ionizable nitrogen within the LAF domain appears to impact on ultrastructure formation and membrane curvature. For 8Oc-K-B2-1:4 (1621) the ionizable nitrogen is positioned centrally, enabling the maintenance of an Ia3d phase across the entire pH range. Conversely, closer proximity between the LAF nitrogen and the Stp headgroup (12Bu-K-B2-1:4) may promote an even more cone-shaped geometry resulting in the presence of a H_II_ phase at neutral pH and the smallest lattice constant (Fig. 4D,E). Functionally, these structural characteristics may contribute to a curvature-driven, less pH-dependent endosomal escape mechanism. This explanation is supported by the moderate impact of bafilomycin A1 on luciferase expression (only 1.6- to 4.5-fold reduction, see Table 1). In addition, large and bright Galectin-8-mRuby3 spots, characteristic of extensive endosomal rupture and rapid cytosolic cargo release were observed (Fig. S8D and [32]). Hence, endosomal escape of Bundle XPs may occur from early endosomes as indicated by recent studies demonstrating that membrane rupture events predominantly originate in these compartments rather than in late endosomes or lysosomes [87]. Early endosomal escape would also be consistent with the observed very fast transfection kinetics (see Fig. S5 and [80]) and higher mRNA transfection efficacy of Bundle XPs when transfected *in vitro* and upon i.v. administration *in vivo* [32], or also as mRNA or siRNA LNPs [41,42].

To further elucidate the structure–activity relationship of Bundle XPs, their ultrastructural properties were examined to predict their relative transfection efficacies. At physiological pH, all evaluated Bundle XPs form a Ia3d bicontinuous cubic phase, with 12Bu-K-B2-1:4 (1752) showing an equilibrium between Ia3d, H_ll_ and Pn3m. Their relative fusogenic potential was evaluated through fusion (pore formation) energies, comprising bending and Gaussian contributions:

$$\text{Δ}G_{fusion\;}\text{Δ}G_{bend\;}+\text{Δ}G_{Gaus\;}$$

The Gaussian contribution can be expressed as $\text{Δ}G_{Gaus\;}=-4\pi \tilde{k}$ [88], and the bending contribution is defined as [89]:

$$\text{Δ}G_{bend\;}=G_{pore\;}-G_{initial\;}=2\kappa \int \left[{\left(H_{pore\;}-H_{0}\right)}^{2}-{\left(H_{initial\;}-H_{0}\right)}^{2}\right]dA$$

Here, *H*_*pore*_ represents the mean curvature of the transient pore formed during fusion events, *H*_*initial*_ is the mean curvature of the initial *Ia3d* phase, and *H*_0_ denotes the spontaneous curvature of the XPs. In summary, this term reflects how much the membrane must deform relative to its initial state. Given the similar chemical composition and preferred mesophases of Bundle XPs, variations in *κ* are not expected to vary significantly. Hence, differences in the fusion barriers may be dominated by the bending contribution. The mean curvature of the Ia3d phase scales with the lattice constant *a* as *H*_*initial*_ 1/*a*^2^. At neutral pH, the lattice constants of the Ia3d phases are 17.0, 15.7, and 15.1 nm for 1621 (8Oc-K-B2-1:4), 1814 (12Bu-O-B2-1:4), and 1752 (12Bu-K-B2-1:4), respectively. These estimations indicate that fusogenicity (and correspondingly endosomal escape and transfection efficacy) should be highest for 1752, followed by 1814 and 1621. In analogy, the relative transfection efficacies display the same trend (Table 2). The comparatively small difference between 12Bu-K-B2-1:4 (1752) and 12Bu-O-B2-1:4 (1814) may be attributed to the presence of additional mesophases for 1752 at high pH, which could enhance transfection by inducing extra perturbations. Furthermore, enhanced mixability of Bundle XPs with endosomal lipids at acidic pH may positively impact on fusion, supported by the moderate response to bafilomycin A1 (Table 2, Fig. S6, Fig. S20B). Overall, increased mean curvature of Bundle XPs within the Ia3d phase benefits endosomal escape and corresponding mRNA expression.

Maximum endosomal escape, however, might not be the optimum for all therapeutic applications. Pronounced and persistent Gal8 recruitment has been recently reported as a marker for irreversible endosomal damage and cytotoxicity [10]. In this sense, the extensive fluorescence patterns visualized and quantified for Bundle XPs along with correlations between Galectin-8 recruitment and relative metabolic activity (Fig. S8) likely reflect highly efficient yet mechanically aggressive escape routes, emphasizing that curvature-driven efficacy must be balanced against endosomal and cellular integrity.

At low mRNA doses, adverse effects were negligible as relative metabolic activity and transfection efficacy remained high [32]. At higher doses, however, potency was accompanied by cytotoxicity. In recent work by Steffens et al. [80], both U-shape and Bundle XPs were altered by inserting additional linker motifs, either bioreducible disulfide building blocks (ssb) or non-reducible hydrophobic spacers, between the hydrophilic headgroup and lipophilic tails. These spacer-analogues were compared to the original XPs in terms of efficacy, underlying biological mechanisms and cytotoxicity. Notably, they induced less improved cellular proliferation and reduced apoptotic and necrotic events indicating that modification of the linker region could pave the way towards potent nucleic acid delivery and improved biocompatibility.

Colloidal stability displays a critical safety parameter for polyplexes, as they are prone to aggregation in the presence of salts, proteins and serum. According to Folda et al. [90], top-performing U-shape (12Oc-K-U1-1:2, 1611) and Bundle carriers (8Oc-K-B2-1:4, 1621 and 12Bu-K-B2-1:4, 1752) aggregated after 2 h incubation in 90% fetal bovine serum at 37 °C, while their efficacy remained unaffected. PEGylation effectively prevented aggregation, requiring minimal amounts of 3–10 mol% DMG-or DSPE-PEG for Bundles and 10 mol% DSPE-PEG-N3 for U-shape XPs.

Translating *in vitro* to *in vivo* performance remains challenging due to the complex environment that nanoparticles encounter upon systemic administration [91–94]. Nevertheless, certain structural features observed *in vitro* may provide qualitative guidance for *in vivo* efficacy. For Bundle XPs, SAXS measurements of LAF-Stp XP/lipid bulk phases revealed inverted, fusogenic mesophases. TEM imaging of intact polyplexes further confirmed that these ultrastructures are not solely induced upon membrane interaction but may be already present in the assembled nanoparticles (Fig. S18C and D). Consistently, these characteristics are reflected in the unexpectedly high transfection efficacy of Bundle LAF-XP mRNA polyplexes in full serum, even at ultra-low doses—down to 3 pg mRNA per well (10,000 cells) *in vitro* and 1 µg mRNA per mouse *in vivo*. Similar to observations for lipid nanoparticles [93], these findings indicate that internal organization of LAF-Stp XP containing polyplexes and their specific membrane interactions are key determinants of delivery potency, underscoring the importance of combining detailed structural analysis with *in vitro* and subsequent *in vivo* studies to rationally design carriers for potent and safe mRNA delivery.

The distinct behavior of the two different (U-shape and Bundle) topologies of LAF-Stp XPs can be further set in a broader context regarding structure–activity-relationships for ionizable amphiphilic systems [12,95–99]. Previous studies on Janus-type dendrimers [30,31,100,101] have elucidated that even minimal constitutional modifications such as the inversion of ester linkages or repositioning the ionizable amine groups profoundly affect molecular arrangement and formation of different ultrastructural types including columns and spheres. These subtle variations directly translate into altered biodistribution and mRNA delivery efficacy of corresponding nanoparticle formulations [30]. Moreover, physicochemical parameters such as the effective pKa and consequently ionizability and biological activity can be tuned through modifications in the linker domain. In particular, linker length displays a crucial parameter. Longer linkers may exhibit steric and electronic inductive effects, whereas shorter linkers result in a closer location to electron-withdrawing carbonyl groups, thus lowering the effective amine pKa. These alterations directly modulate the protonation behavior and, in turn, the biological performance of these systems [31,95]. Lipophilic tail region displays another structural feature, crucial for endosomal escape [13,102]. Studies on branched-tail ionizable lipidoids have resolved that in contrast to linear tails, branching domains enhance ionization efficiency at endosomal pH, thus facilitating endosomal escape and delivery efficacy [95]. These results are in line with previously established concepts for phospholipids in which lipid geometry demonstrates a key determinant in lipid phase formation and efficacy. While cylindrical lipids such as DSPC stabilize bilayers, cone-shaped lipids such as DOPE promote fusogenic phases with negative curvature [65,103]. It is not surprising that LAF_4_-Stp_1_ XP bundles, containing eight short alkyl residues (two per LAF unit) attached to Stp in a dendron-like architecture, form lipid phases with negative curvatures. Also here, as outlined in the current work, small changes in topology and linker domains may have a strong effect on structure and efficacy. Recently, related LAF-based peptides with impressive activity as peptide ionizable lipids (PIL) as part of mRNA LNPs were reported [97]. The cone-shaped LAF motif was directly incorporated into α-amino acids that were used to form potent transfection competent peptides. Interestingly, extension with additional amino acids and screening in an evolution-type setting identified mRNA ‘PILOT’ LNPs for *in vivo* tissue-targeted delivery and prime editing without need for biological targeting ligands [97].

## Conclusion

The current study highlights how the spatial interplay of molecular domains, including a polar Stp headgroup, pH-responsive lipophilic LAF tail regions and connecting linkers in distinct topologies dictate structural organization of LAF-Stp XPs, their pH-responsive self-assembly and distribution between water and lipidic phase, and their performance in intracellular delivery and mRNA transfection. The balance between hydrophilic and lipophilic moieties along with their chemical constitution define amphiphilicity, curvature and hydration behavior, ultimately shaping both ultrastructure formation and biological properties. By combining MD simulations and SAXS experiments, a coherent picture emerges that connects self-assembly with mesoscale organization. Flexible U-shaped carriers maintain lamellar order and require pH-triggered rearrangements or partial disassembly to enable localized, non-fusion-based membrane permeabilization for endosomal escape. Bundle XPs containing LAF domains in dendritic constrained constitution, in contrast, form intrinsically curved, bicontinuous cubic phases and inverted phases that promote membrane disruption largely independent of pH. Altogether, these results highlight that topology and domain act as key regulators of structural adaptability and endosomal escape mechanisms. The work emphasizes that higher-order organization is not merely a structural consequence but a mechanistic feature that underlies the function of amphiphilic carriers.

## Supplementary Material

Supplementary data to this article can be found online at https://doi.org/10.1016/j.mattod.2026.103353. The supplementary data consists of an experimental section, supporting data and references.

Appendix A. Supplementary Data

## Figures and Tables

**Table 1:** Chemical characteristics of U-shape and Bundle xenopeptides (XP).

ID	code	topology	Stp:LAF ratio	spacer	lipo amino fatty acid (LAF)
**U-shapes**					
1611	12Oc-K- U1-1:2	U1	1:2	L-lysine	12Oc
1827	12Oc-O- U1-1:2	U1	1:2	L-ornithine	12Oc
1718	12Oc-K- U1-1:4	U1	1:4	L-lysine	12Oc
1960	12Oc-O- U1-1:4	U1	1:4	L-ornithine	12Oc
**Bundles**					
1621	8Oc-K-B2- 1:4	B2	1:4	L-lysine	8Oc
1813	8Oc-O-B2- 1:4	B2	1:4	L-ornithine	8Oc
1752	12Bu-K- B2-1:4	B2	1:4	L-lysine	12Bu
1814	12Bu-O- B2-1:4	B2	1:4	L-ornithine	12Bu

**Table 2:** Relative transfection efficacies of mRNA polyplexes formed with distinct LAF-Stp XPs at an mRNA concentration of 12.5 µg mL^-1^ in N2a and HeLa cells. Luciferase expression was determined 24 h after transfection. Impact of endosomal acidification on transfection efficacy in the absence or presence of the endosomal vATPase inhibitor bafilomycin A1 in N2a cells 4 h post transfection. All experiments were performed in biological triplicates at a dose of 15 ng mRNA per well. Statistical analysis was conducted via unpaired Student’s two-tailed *t*-test with Welch’s correction in GraphPad Prism 10. Significance levels: ns p > 0.05; * p ≤ 0.05; ** p ≤ 0.01; *** p ≤ 0.001; **** p ≤ 0.0001.

Carrier ID	Code	Relative efficacy compared to 1611			Dependency on endosomal acidification		
		N2a	HeLa		– BafA1/ +BafA1 in N2a	p-value	Statistical significance
**1611**	12Oc- K-U1:2	1	1		14.6	0.0015	**
**1827**	12Oc- O-U1:2	1.5	1.1		23	0.0462	*
**1718**	12Oc- K-U1:4	0.9	1		20.9	0.0123	*
**1960**	12Oc- O-U1:4	0.3	0.7		8.2	0.001	***
**1621**	8Oc-K- B2-1:4	1.5	1.2		1.6	0.1199	ns.
**1813**	8Oc-O- B2-1:4	5	3.7		4.5	<0.0001	****
**1752**	12Bu- K-B2-1:4	3.7	3.8		2	0.0212	*
**1814**	12Bu- O-B2- 1:4	1.6	1		3.1	0.1327	ns.
**Controls**							
**Controls succPEI**	–	0.3	0.3				
**MC3**	–	3.6	3.8				

## Data Availability

Data will be made available on request.
