# Supplementary material for: Chemical topology of lipo-amino fatty acid mRNA carriers triggers distinct lipid bulk phases and endosomal escape mechanisms
Source: Mater Today (Kidlington). Author manuscript; Available in PMC 2026 Oct 31. (PMC7619666; doi:10.1016/j.mattod.2026.103353)
Supplement: Supplementary file 1 [file EMS221002-supplement-Appendix_A__Supplementary_Data.pdf]

Chemical topology of lipo-amino fatty acid mRNA carriers triggers distinct lipid bulk phases and endosomal escape mechanisms

Melina Grau[1+], Ekaterina Kostyurina[2+], Johanna Seidl[1], Nora Martini[3], Benjamin Winkeljann[3,4,5,6], Olivia M. Merkel[3,4,5], Joachim O. Rädler[2,4]* and Ernst Wagner[1,4]**

[1] Pharmaceutical Biotechnology, Department of Pharmacy, Ludwig-Maximilians-Universität München, Butenandtstrasse 5-13, Munich 81377, Germany

[2] Faculty of Physics, Ludwig-Maximilians University, Geschwister-Scholl-Platz 1, Munich, Germany

[3] Pharmaceutical Technology and Biopharmaceutics, Department of Pharmacy, Ludwig-Maximilians-Universität München, Butenandtstrasse 5-13, Munich 81377, Germany

[4] Center for NanoScience (CeNS), Ludwig-Maximilians-Universität München, Munich 80799, Germany

[5] Comprehensive Pneumology Center (CPC-M), Ludwig-Maximilians-Universität München, Member of the German Center for Lung Research (DZL), Munich 81377, Germany

[6] Pharmaceutical Engineering and Technology Research Scientists (PETRS)

Table of Contents

1. Experimental section 2
2. Supporting data 36
3. References... 60

1. Experimental section

Materials

8-Aminooctanoic acid, octanal, dodecanal, sodium cyanoborohydride, triisopropylsilane (TIS), 1-hydroxybenzotriazole (HOBt), benzotriazol-1-yloxytripyrrolidinophosphonium hexafluorophosphate (PyBOP), Triton X-100, N,N-diisopropylethylamine (DIPEA), hydrazine monohydrate, phenol, potassium cyanide and super-DHB (9:1 mixture of 2,5-dihydrocybenzoic acid and 2-hydroxy5-methoxybenzoic acid) were purchased from Sigma Aldrich (Munich, Germany). 4-aminobutyric acid was obtained from TCI Germany (Eschborn, Germany). Ninhydrin was purchased from AppliChem (Darmstadt, Germany). 2-Chlorotrityl chloride resin, dimethylformamide (DMF), piperidine, Fmoc-L-Lysine(Fmoc)-OH, Fmoc-L-Lysine(Dde)-OH and Boc-L-Lysine(Fmoc)-OH were obtained from Iris Biotech (Marktredwitz, Germany). Succinylated branched polyethyleneimine (PEI) 25 kDa (succPEI; succinylation degree of 10%) was synthesized and analyzed as described previously [1]. Starting products branched PEI (brPEI) 25 kDa as well as poly(2-ethyl-2-oxazoline) were purchased from Sigma Aldrich (Munich, Germany). HEPES was obtained from Biomol (Hamburg, Germany), disodium ethylenediaminetetraacetic acid (EDTA) as well as glucose from Merck (Darmstadt, Germany). Cholesterol was purchased from Sigma Aldrich (Munich, Germany), while 1,2-Distearoyl-sn-glycero-3-phosphocholin (DSPC), 1,2-dioleoyl-sn-glycero-3-phosphocholine (DOPC) and DMG-PEG 2000 were obtained from Avanti Polar Lipids (Alabaster, AL, USA). Solid-phase synthesis reactors (polypropylene reactors with polytetrafluoroethylene (PTFE) frits and Luer stopper) were purchased from Multi-SynTech (Witten, Germany). Deuterated solvents were purchased from Eurisotop (Fluorochem, Hadfield, UK). All further solvents and other reagents were purchased from Sigma-Aldrich (Munich, Germany), Iris Biotech (Marktredwitz, Germany), Merck (Darmstadt, Germany), or AppliChem (Darmstadt, Germany). RNase-free water was purchased from Sigma Aldrich (Munich, Germany) and heparin-sodium salt 5000 I.U. mL^{-1} from B. Braun SE (Melsungen, Germany). All cell culture consumables were obtained from Faust Lab Science (Klettgau, Germany). Cell culture media, fetal bovine serum (FBS), antibiotics, as well as trypsin/EDTA were purchased from Sigma Aldrich (Munich, Germany) and PANBiotech (Aidenbach, Germany). Cell culture 5× lysis buffer, and D-luciferin sodium salt were obtained from Promega (Mannheim, Germany); 3-(4,5- dimethylthiazol-2-yl)-2,5-diphenyiltetrazolium bromide (MTT), dithiothreitol (DTT), adenosine 5′-triphosphate (ATP) disodium salt trihydrate, coenzyme A trilithium salt, bafilomycin A1, glycylglycine, magnesium chloride ($MgCl_2$) were purchased from Sigma Aldrich (Munich, Germany). µ-Slides 8 well ibidiTreat were obtained from ibidi GmbH (Gräfelfing, Germany), non-transparent black flat bottom 96-well plates from Thermo Fisher Scientific (Schwerte, Germany). Quant-iT™ RiboGreen RNA Assay-Kit was

purchased from Thermo Fisher Scientific (Schwerte, Germany). The murine neuroblastoma cell line Neuro2a (N2a) was obtained from the American Type Culture Collection (ATCC; Manassas, Virginia, USA), the human adherent cervix carcinoma cell line HeLa from the German Collection of Microorganisms and Cell Cultures (DSMZ; Braunschweig, Germany). CleanCap® Fluc mRNA (5 moU) was purchased from Trilink Biotechnologies (San Diego, CA, USA).

Electrospray ionization – mass spectrometry (ESI-MS)

ESI-MS was carried out using a Thermo Scientific LTQ FT Ultra Fourier transform ion cyclotron and an IonMax source. Data is presented as [M+X] z after positive ionization. Samples were kindly processed by Dr. Werner Spahl from the analytical core facility at the Department of Chemistry, LMU Munich.

^{1}H-NMR

Spectra were recorded by using a Bruker Avance III HD 400 (400 MHz). Signals were calibrated to the non-deuterated signals of the used solvent as an internal standard (CDCl3 7.26 ppm;). Chemical shifts (δ) were reported in parts per million (ppm). The spectra were analyzed by MestreNova (MestReLab Research x64, Version 10.0). Integration was performed manually.

MALDI-TOF mass spectrometry (MALDI-TOF MS)

For MALDI-TOF MS, a 10 mg mL^{-1} solution of super-DHB (9/1 (w/w) mixture of 2,5-dihydroxybenzoic acid and 2- hydroxy-5-methoxybenzoic acid) in 69.93/30/0.07 (v/v/v) H2O/ACN/TFA served as the matrix. One µL of matrix solution was spotted on a MTP AnchorChip (Bruker Daltonics, Germany). Subsequently, 1 µL of sample solution dissolved in H_2O at a concentration of 1 mg mL^{-1} was added onto the matrix and co-crystallized. Samples were analyzed using an Autoflex II mass spectrometer (Bruker Daltonics, Germany). Spectra were recorded in positive ion mode.

Note: Small deviations (0.19 – 0.25%) between calculated molecular weights and obtained m/z values are attributed to limited mass accuracy of the used device.

Synthesis of Lipo Amino Fatty acids (LAFs)

The protocol was adapted from Thalmayr et al.[2] with minor modifications. Briefly, 1 equivalent (eq.) of the respective amino fatty acid (4-aminobutyric acid or 8-aminoctanoic acid) was dissolved in a 2:3 (v/v) mixture of dry methanol (MeOH) and tetrahydrofuran (THF). Subsequently, 2 eq. of the indicated fatty aldehyde (octanol or dodecanal) and the reducing agent sodium cyanoborohydride ($NaBH_3CN$) were added. The mixture was stirred at room temperature (RT) for 48 hours (h). After completion, the solvents were removed under reduced pressure. The resulting solid residue was dried under high vacuum. The crude product was then re-dissolved in dichloromethane (DCM) and filtrated. The filtrate was concentrated and purified by siliga gel column chromatography (solvent gradient DCM/MeOH 50:1 – 9:1 v/v). Product elution was monitored by thin-layer chromatography (DCM/MeOH 9:1 (v/v)) and staining with basic potassium permanganate ($KMnO_4$) solution. The obtained products were analyzed by ESI-MS and ^{1}H-NMR.

8Oc

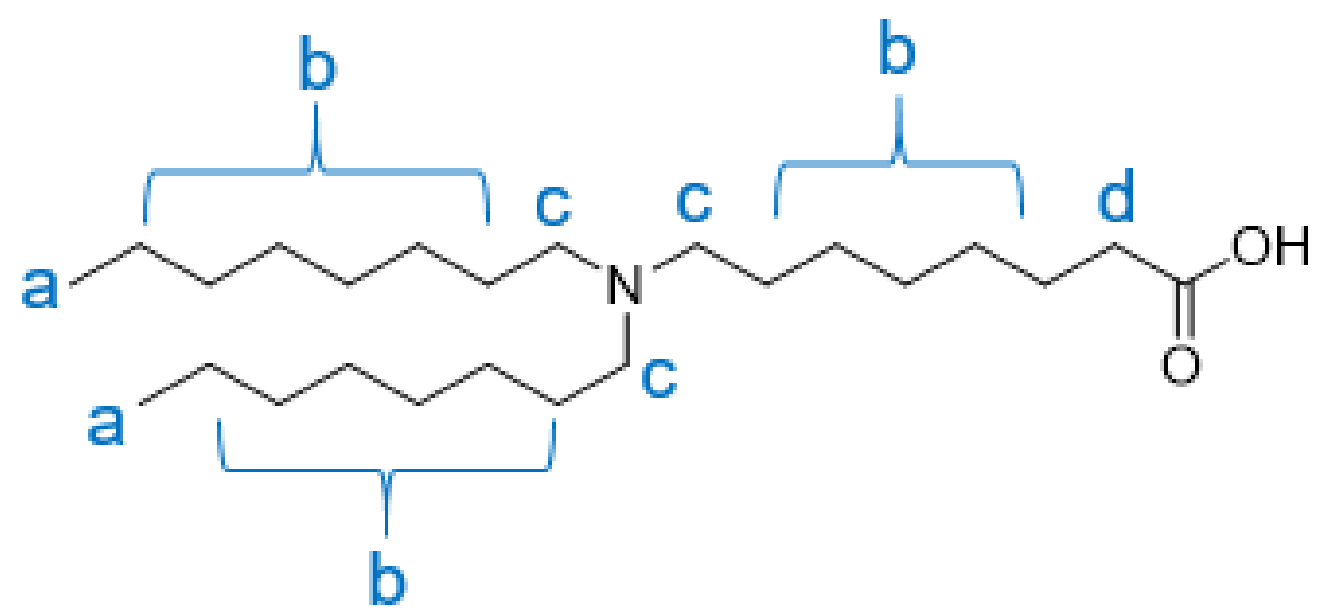

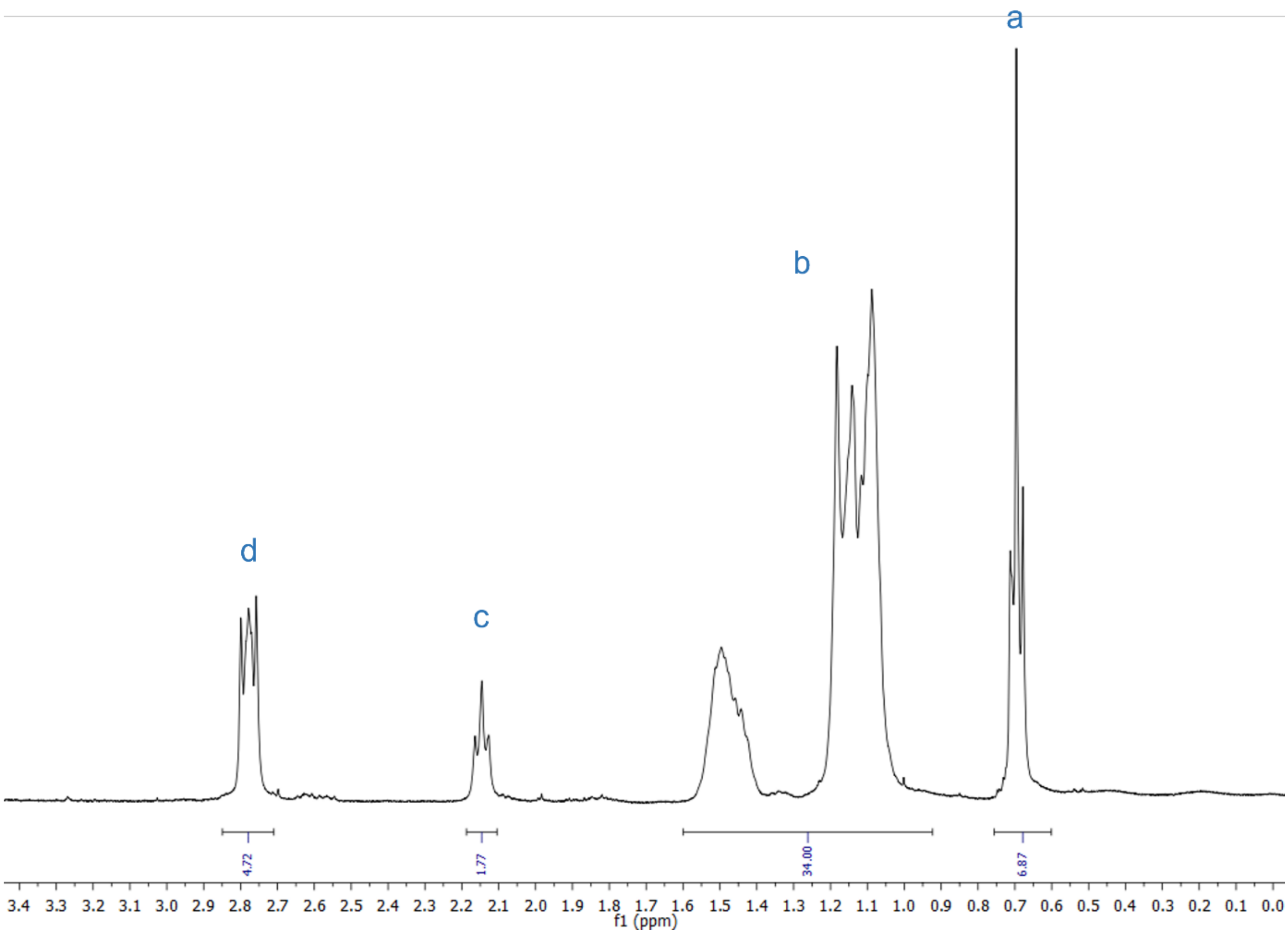

^{1}H-NMR (400 MHz, $CDCl_3$) δH (ppm) = 0.601 – 0.756 (m, 6H, -CH_3), 0.923 - 1.6 (m, 34H, -CH_2-), 2.71- 2.849 (m, 6H, -CH_2-N-), 2.105 - 2.187 (m, 2H, -CH_2-CO)

ESI-MS: calculated for $C_{24}H_{49}NO_2$ = 383.38; found: $[M+H]^+$ = 384.40

12Bu

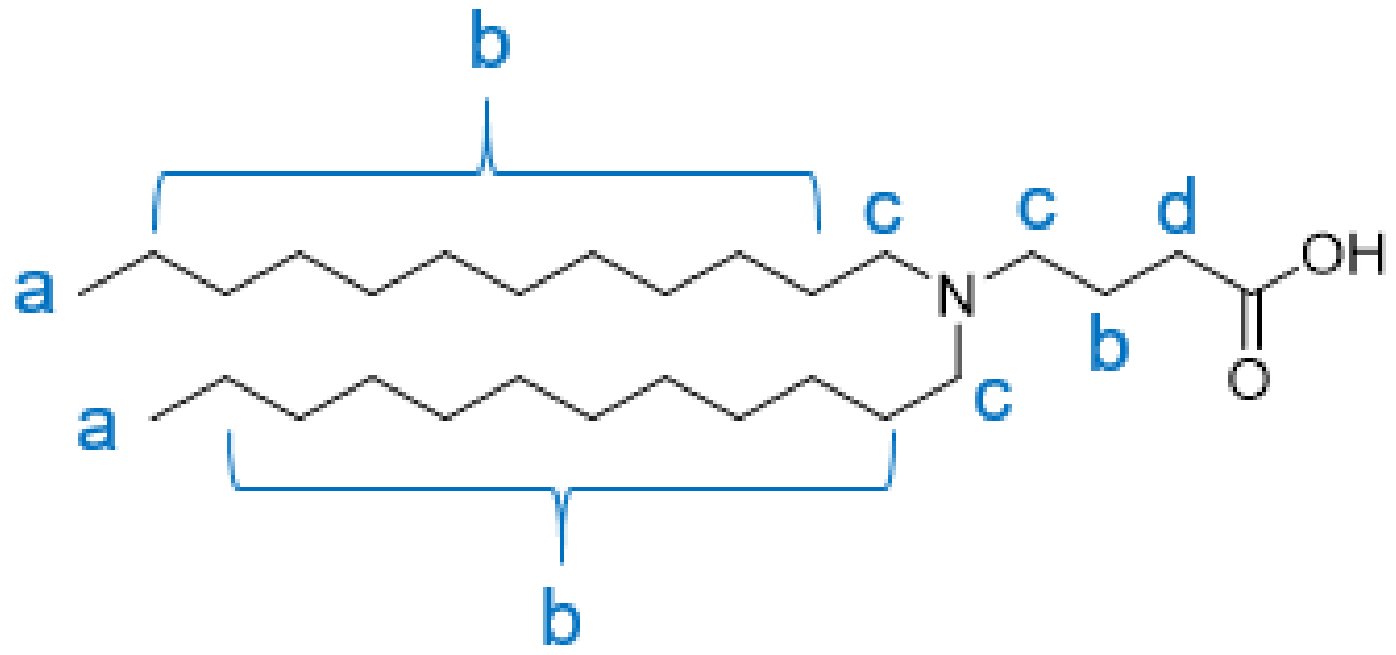

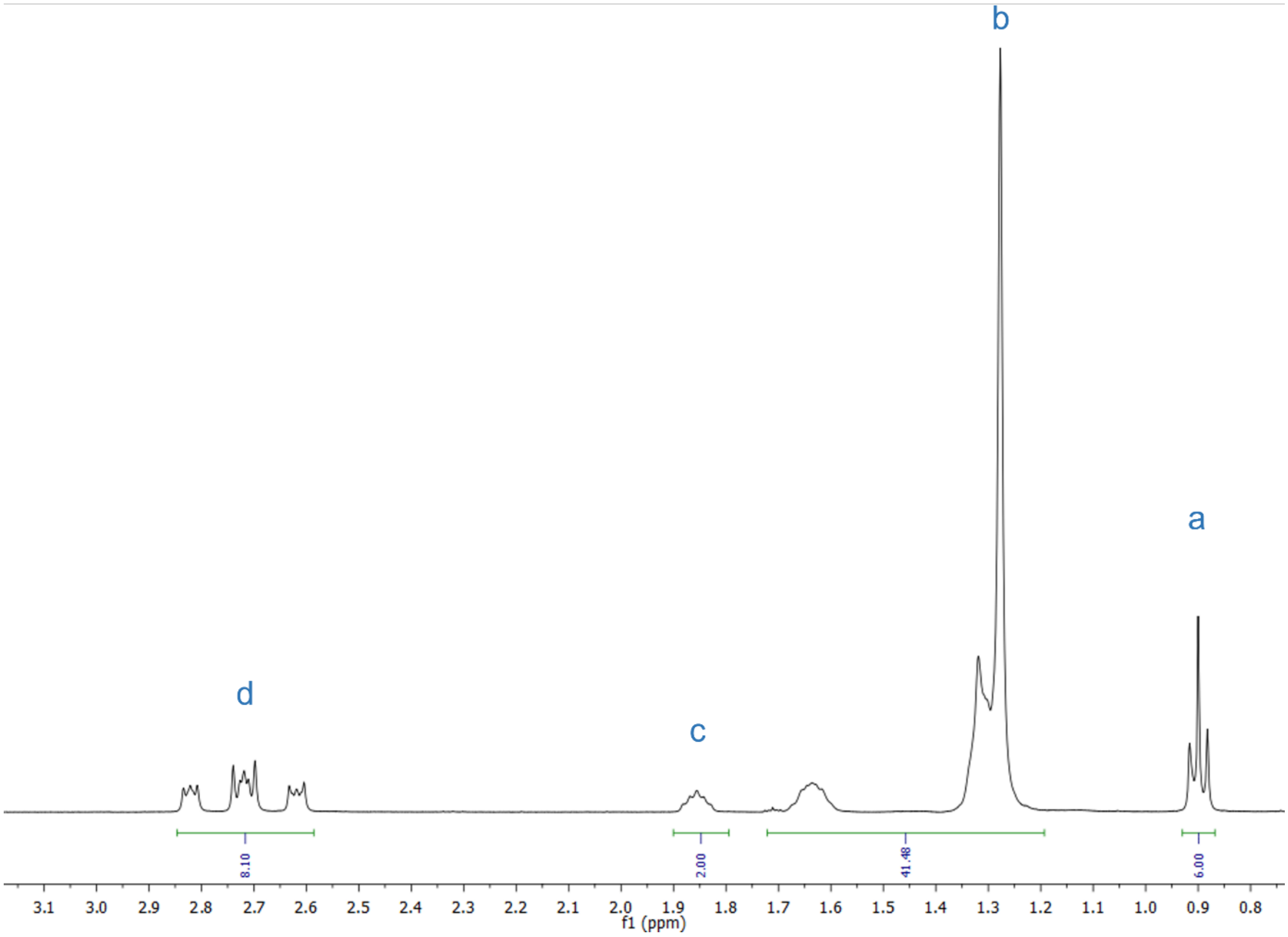

^{1}H-NMR (400 MHz, $CDCl_3$) δH (ppm) = 0.868 - 0.930 (m, 6H, $-CH_3$), 1.193 - 1.722 (m, 42H, $-CH_2-$), 2.586 - 2.847 (m, 6H, $-CH_2-N-$), 1.795 - 1.9 (m, 2H, $-CH_2-CO$)

ESI-MS: calculated for $C_{28}H_{57}NO_2$ = 439.44; found: $[M+H]^+$ = 440.44

12Oc

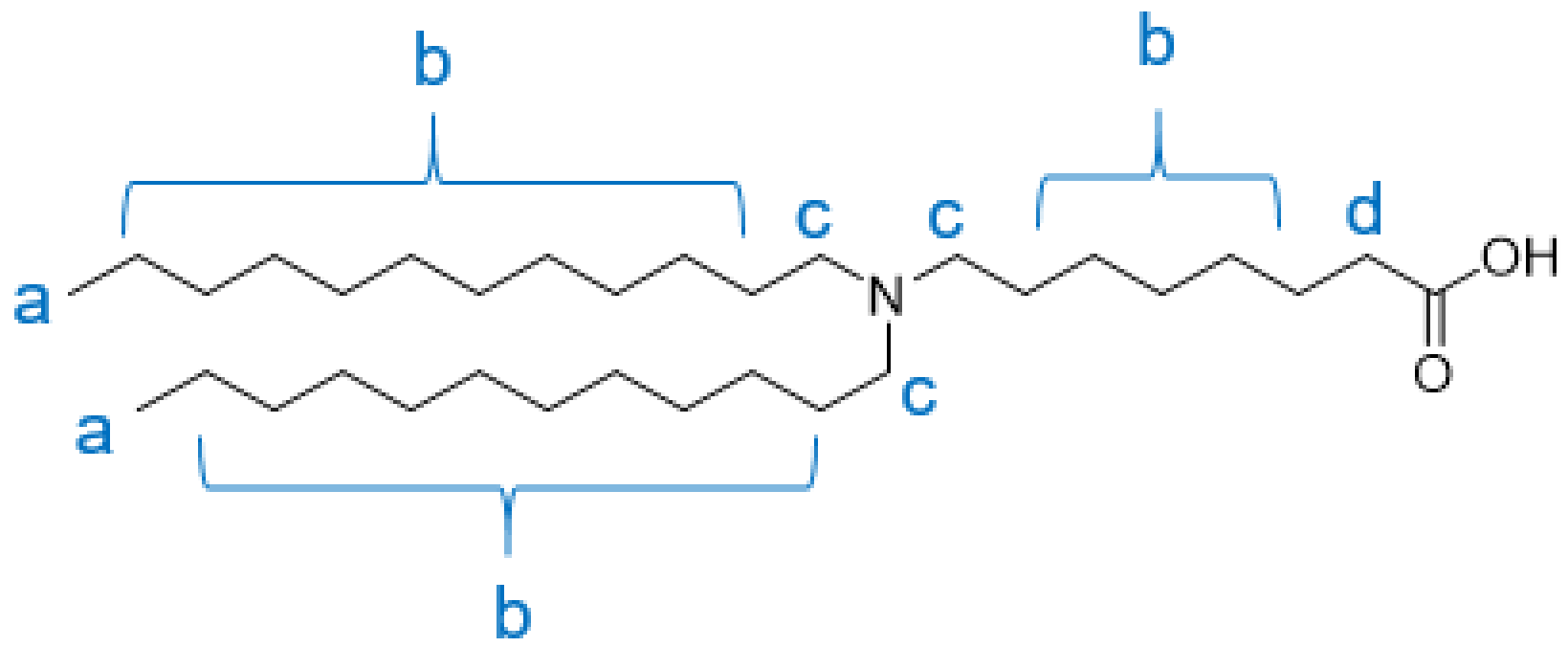

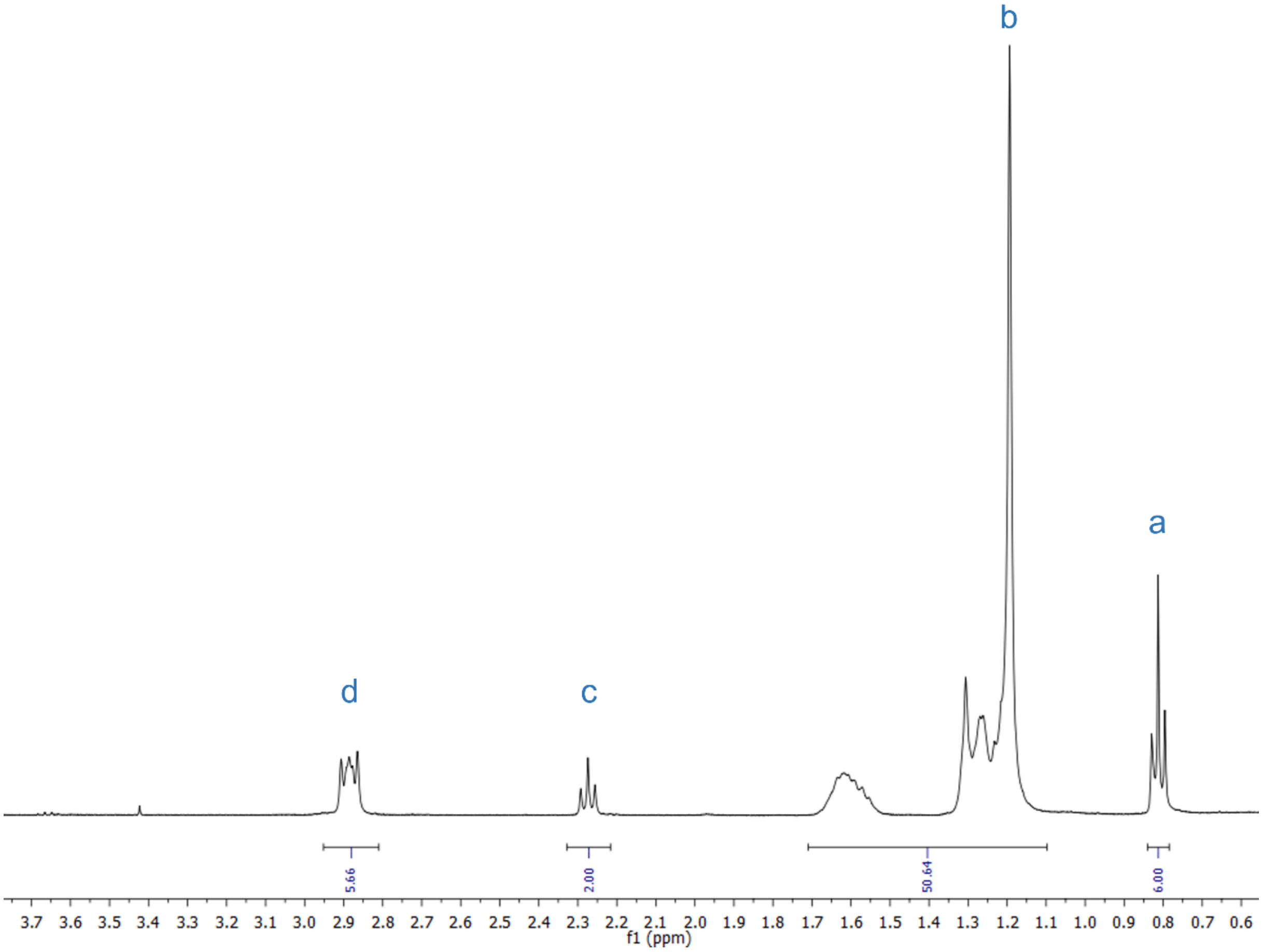

^{1}H-NMR (400 MHz, $CDCl_3$) δH (ppm) = 0.785- 0.84 (m, 6H, $-CH_3$), 1.098 - 1.71 (m, 50H, $-CH_2$), 2.81 - 2.952 (m, 6H, $-CH_2$-N-), 2.216 - 2.327 (m, 2H, $-CH_2$-CO)

ESI-MS: calculated for $C_{32}H_{65}NO_2$ = 495.50; found: $[M+H]^+$ = 496.87

Solid phase assisted LAF-xenopeptide synthesis

Resin loading. The desired amount of 2-chlorotrityl resin was transferred to a syringe reactor and swelled in anhydrous DCM for 20 minutes (min). Depending on the peptide topology, either the C-terminal amino acid or building block was dissolved in dry DCM, followed by the addition of 3-fold molar excess of DIPEA. The solution was immediately added to the syringe and incubated for 75 min. To cap residual reaction sides on the resin, a solution containing 2 mL dry DCM, 1 mL dimethylformamide (DMF), 1 mL MeOH and 0.25 mL DIPEA was added and incubated for 60 min. Afterwards, the solution was drained, and the resin washed three times each with DMF and DCM, followed by drying under vacuum. Resin loading was determined by photometric quantification of free fluorenyl derivatives after piperidine deprotection. Therefore, three samples of the resin (5-10 mg) were treated with 1 mL deprotection solution consisting of 20% piperidine in DMF (v/v) for 75 min under constant shaking. After incubation, the samples were vortexted and 25 µL of the supernatant were diluted with 975 µL DMF. The absorbance was measured at 301 nm, using 20% piperidine in DMF (v/v) as a blank. Resin loading was calculated using the following equation: resin loading [mmol g -1] = (A x 1000) x (m [mg] x 7800 x df) -1 with df= dilution factor.

Fmoc deprotection. After loading determination, Fmoc protection groups were removed. Therefore, the resin was incubated with Fmoc-deprotection solution (20% piperidine in DMF (v/v)) 3 times for 15 min. Last, the resin was washed with DMF and DCM (three times each).

Coupling of Fmoc-protected amino acids and artificial building blocks. Indicated topologies were synthesized manually by sequential coupling of Fmoc-protected amino acids or the artificial building block succinoyl tetraethylene pentamine (Stp) to the pre-loaded resin. In particular, 4 eq. of the respective amino acid or Stp were dissolved in 1 mL DCM and 8 eq. of DIPEA were added. The solution was mixed with 4 eq. of PyBOP and 4 eq. HOBt, dissolved in 1 mL DMF, transferred to the resin and incubated for 90 min. After incubation, the resin was washed three times each with DMF and DCM. Both, coupling and deprotection steps were monitored with a Kaiser test to confirm the reaction completion. Symmetrical branching points were introduced using Fmoc-L-Lys(Fmoc)-OH or Fmoc-L-Orn(Fmoc)-OH, and asymmetrical branching points by using Fmoc-L-Lys(Dde) or Fmoc-L-Orn(Dde). In case of U-shapes the N-terminal Fmoc-L-Lys(Dde) or Fmoc-L-Orn(Dde) was replaced with Boc-L-Lys(Fmoc) or Boc-L-Orn(Fmoc).

Dde-deprotection. Removal of the Dde protection group [N-(1-(4,4-dimethyl-2,6-dioxocyclohexylidene)ethyl)] was achieved by treating the resin with 2% hydrazine in DMF (v/v) for 15 cycles of 2 min, respectively. Afterwards, the resin was washed with 10% DIPEA in DMF (v/v) (five times), DMF and DCM (three times each).

***LAF coupling*.** The LAF coupling was carried out as described above. Yet, the resin was allowed to stir for 24 h for U-shapes and 72 h for Bundles to ensure reaction completion.

Kaiser Test. A small amount of washed resin was transferred into an Eppendorf reaction tube. One drop of 80% (w/v) phenol in ethanol (EtOH), 5% (w/v) ninhydrin in EtOH and 20 µM potassium cyanide (KCN) in pyridine (1 mL aqueous KCN solution, c = 1mM in 49 mL pyridine) were added. The Eppendorf tube was incubated under shaking at 500 rpm at 99 °C for 3 min**.** A dark blue colour of both resin beads and reaction solution indicated the presence of free amines. Absence of free amines resulted in no change of colour.

***Resin cleavage*.** The resin was dried in vacuo prior to cleavage. For cleavage and removal of acid-labile protection groups, a cleavage cocktail containing TFA/TIS/H_2O (95/2.5/2.5 v/v/v) was added to the resin and stirred for 1 h. The cleavage solution was drained and collected. The resin was washed with DCM two times, and the solutions were combined and dried under constant nitrogen flow.

Purification. The crude product was dissolved in EtOH or EtOH/H_2O (1:1, v/v). For Bundles, the clear solutions were dialyzed against EtOH for 24 h, followed by dialysis against 0.1 mM hydrochloric acid (HCl) for an additional 48 h at 4 °C. In case of U-shapes, the sequences were directly dialyzed against 0.1 mM HCl for 48 h. Depending on the molecular weight of the synthesized xenopeptide, Spectra/Por Dialysis Membranes (Carl Roth, Karlsruhe, Germany) with a molecular weight cut-off (MWCO) of 1 or 2 kDa were used. Afterwards, the compounds were diluted with water and lyophilized. The final products were analysed by MALDI-TOF MS. Stock solutions were prepared in EtOH/H_2O mixtures for U-shapes (1:1 v/v for U1-1:2 and 7:3 v/v for U1-1:4) or EtOH/aqueous HCl (9:1 v/v, 121 mM HCl) for Bundles. The chemical characterization of each xenopeptide is provided in **Table S1**.

1611: **12Oc-K-U1-1:2**

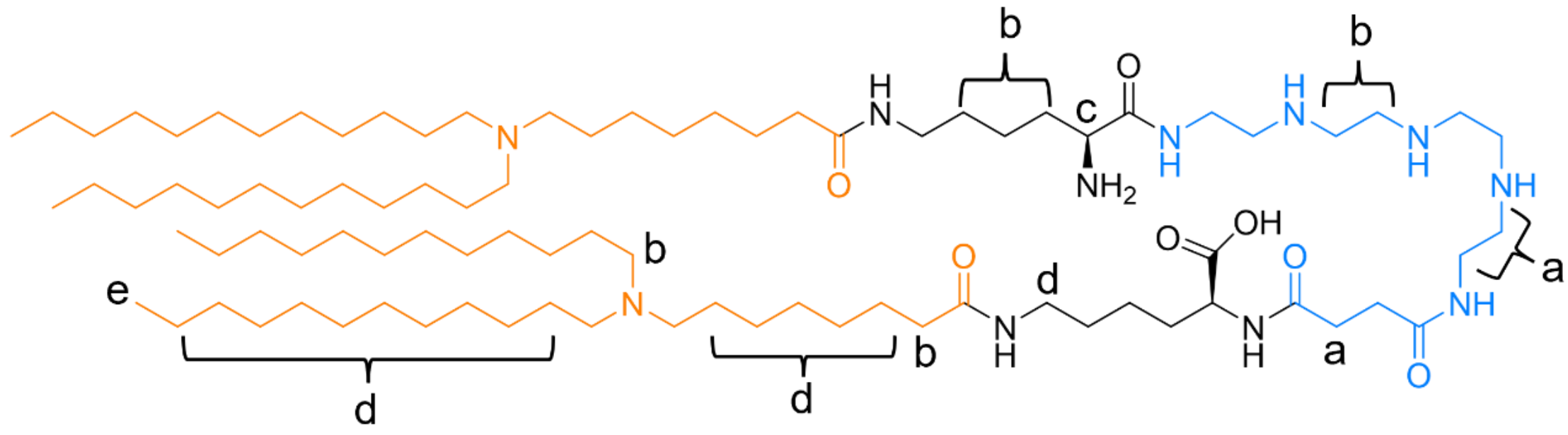

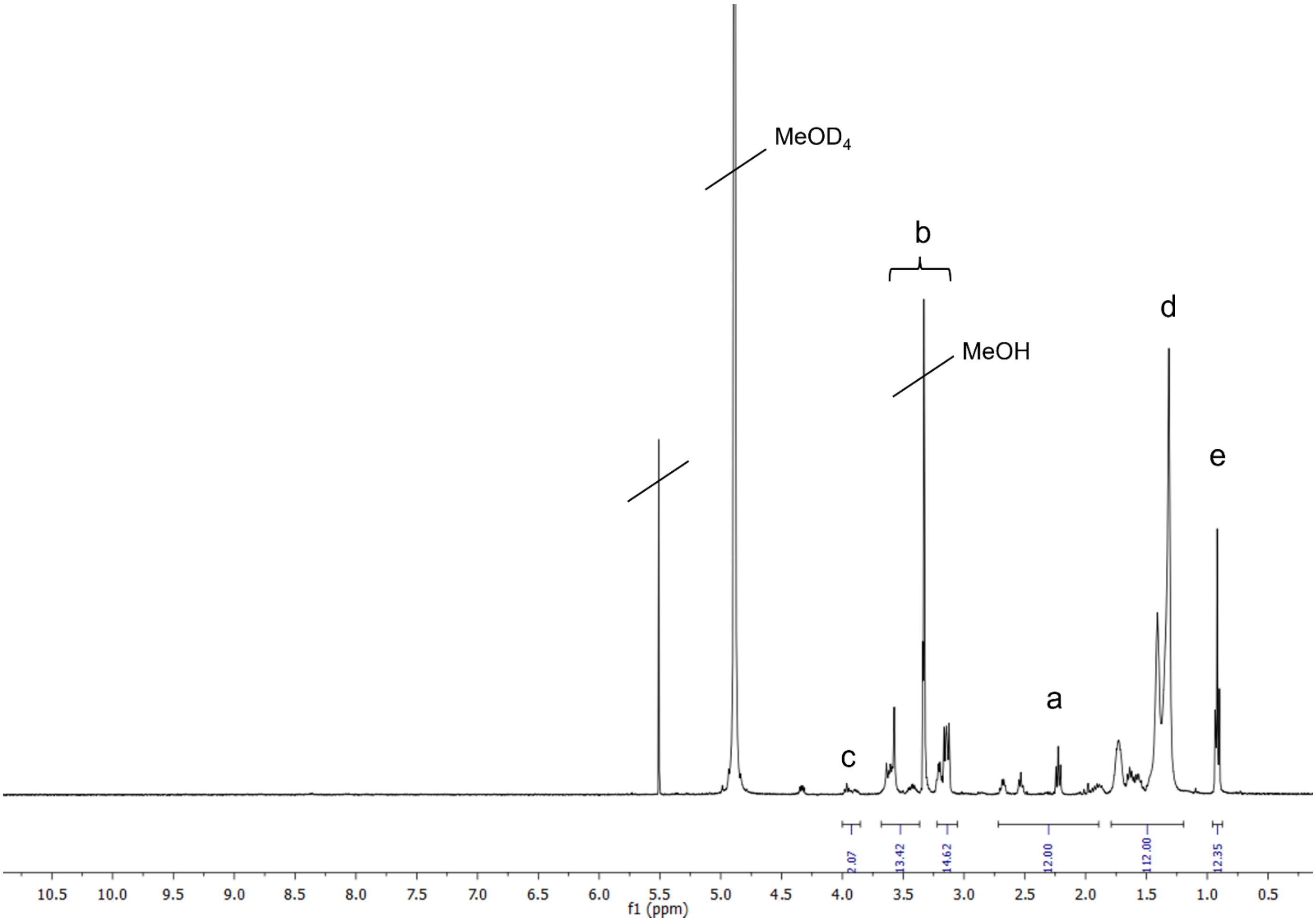

^{1}H-NMR (400 MHz, methanol-d4) δ (ppm) = 0.874 – 0.955 (t, 12H, $-CH_3$, dodecanal), 1.196 – 1.791 (m, 112H, βγδ H lysine, βγδεz H Oc, $-CH_2-$ dodecanal), 1.892 – 2.718 (m, 8H, $-NH-CH_2-CH_2-NH-$ Stp, 4H, $-CO-CH_2-CH_2-CO$ Stp), 3.055 – 3.681 (m, 28H, $-CH_2-N-$ LAF, α H Oc, ε H lysine, $-CO-NH-CH_2-CH_2-NH-$ Stp), 4.291 – 4.383 (m, 2H, α H amino acids).

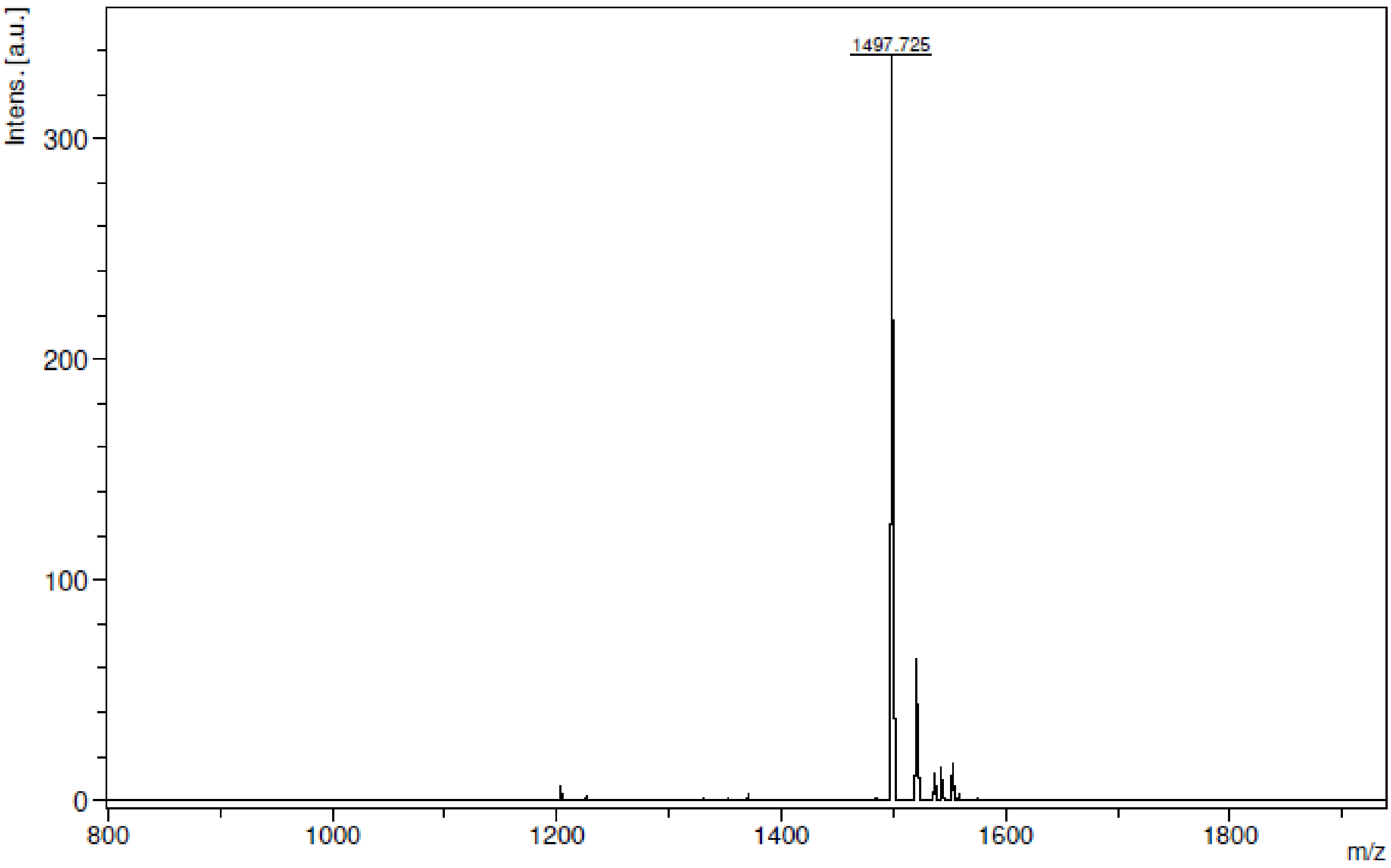

MALDI-TOF MS: calculated for $C_{88}H_{177}N_{11}O_7$ = 1501.42 found: $[M+H]^+$ = 1497.73

1827: **12Oc-O-U1-1:2**

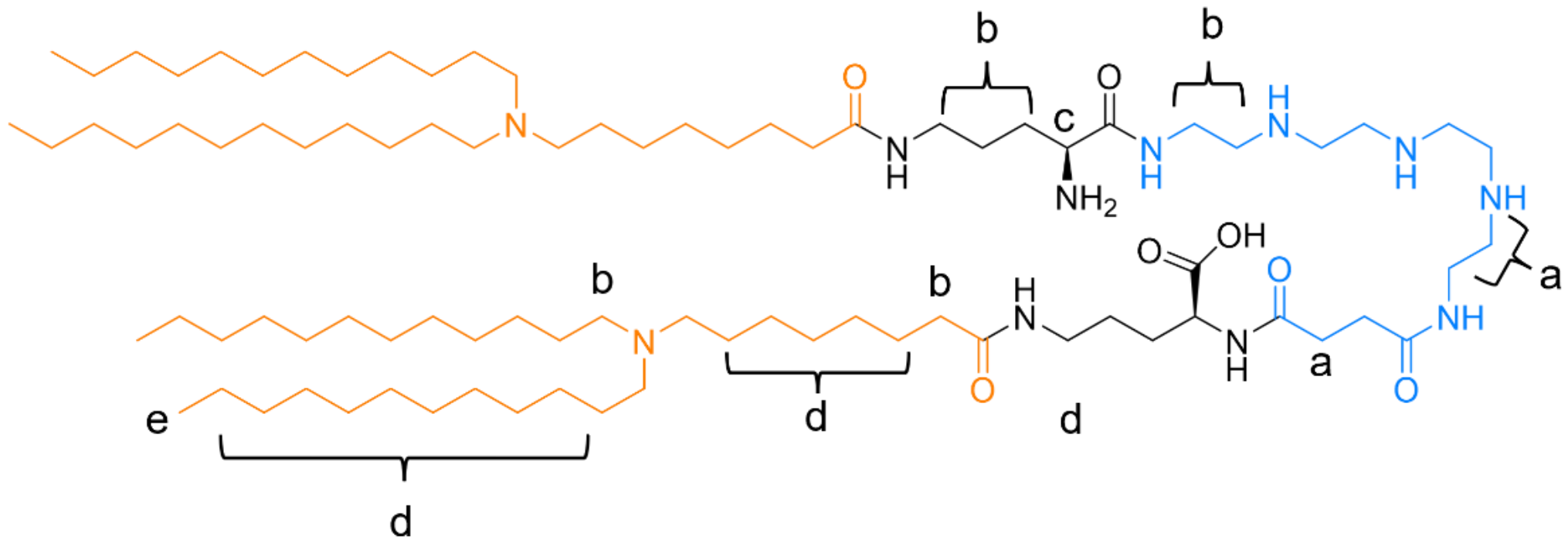

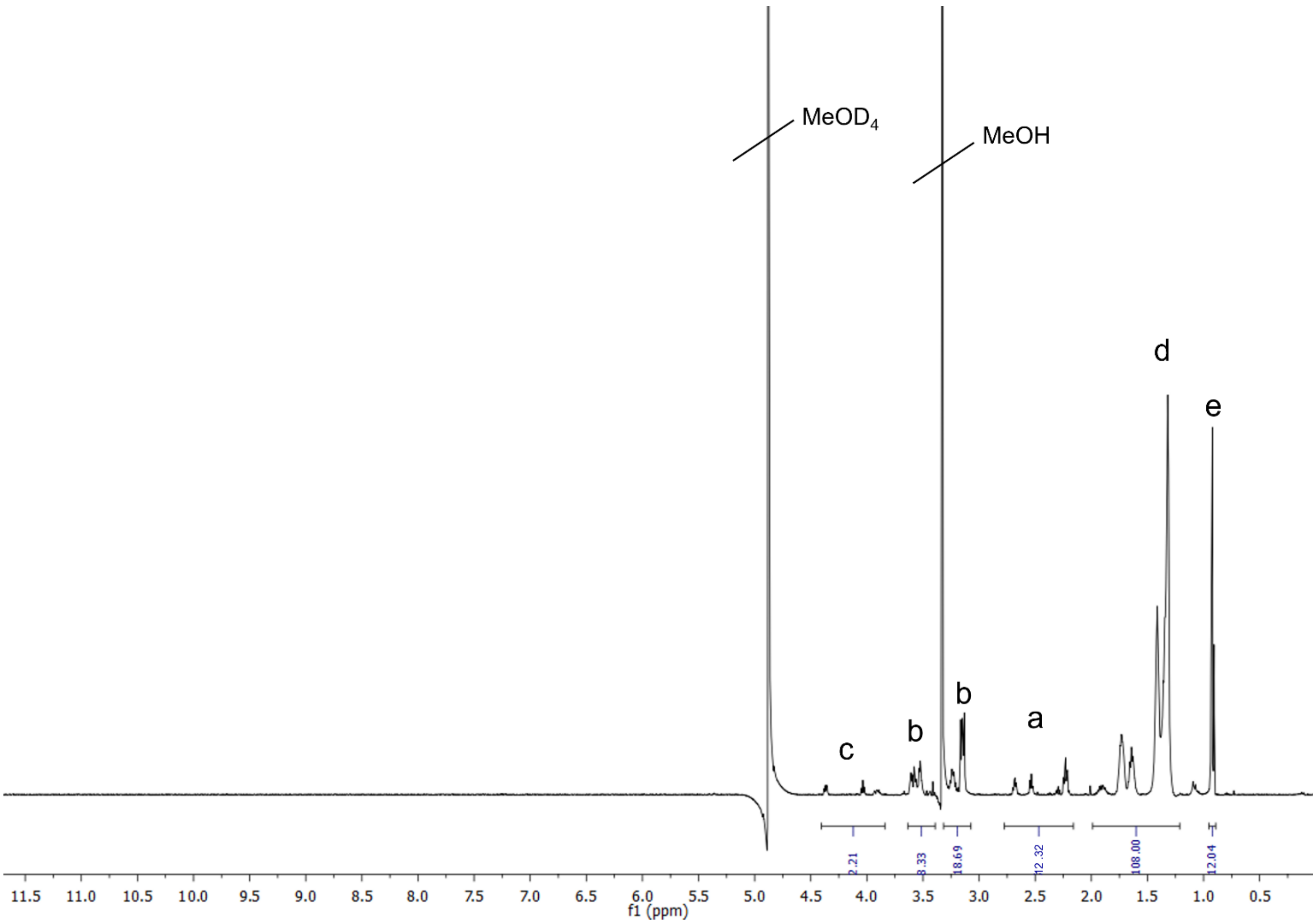

^{1}H-NMR (400 MHz, methanol-d4) δ (ppm) = 0.892 – 0.952 (t, 12H, $-CH_3$, dodecanal), 1.213 – 1.989 (m, 108H, βγ H ornithine, βγδεz H Oc, $-CH_2-$ dodecanal), 2.16 – 2.777 (m, 8H, $-NH-CH_2-CH_2-NH-$ Stp, 4H, $-CO-CH_2-CH_2-CO$ Stp), 3.073 – 3.636 (m, 28H, $-CH_2-N-$ LAF, α H Oc, δ H ornithine, $-CO-NH-CH_2-CH_2-NH-$ Stp), 3.84 – 4.404 (m, 2H, α H amino acids).

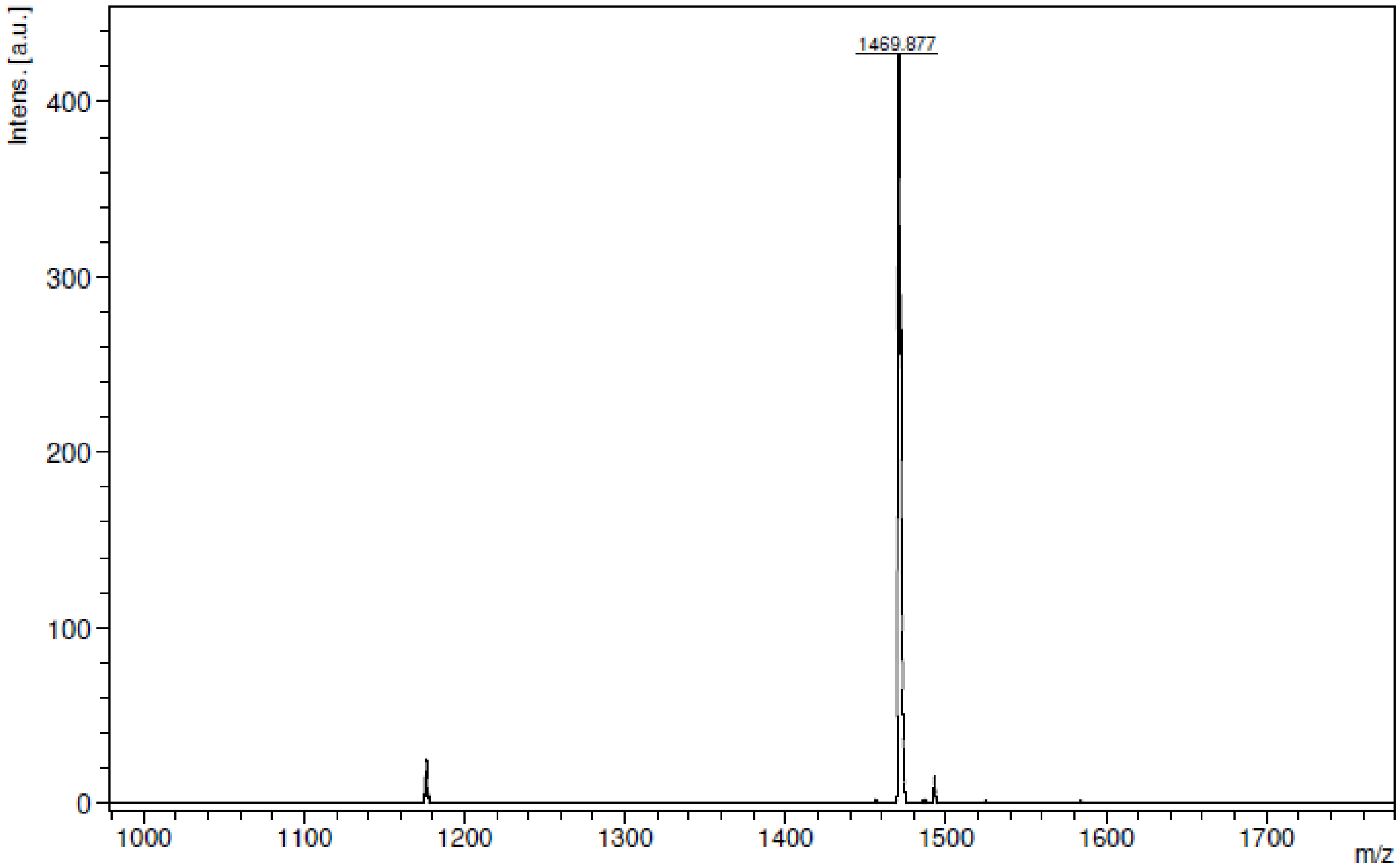

MALDI-TOF MS: calculated for $C_{86}H_{173}N_{11}O_7$ = 1473.40 found: $[M+H]^+$ = 1469.877

1718: **12Oc-K-U1-1:4**

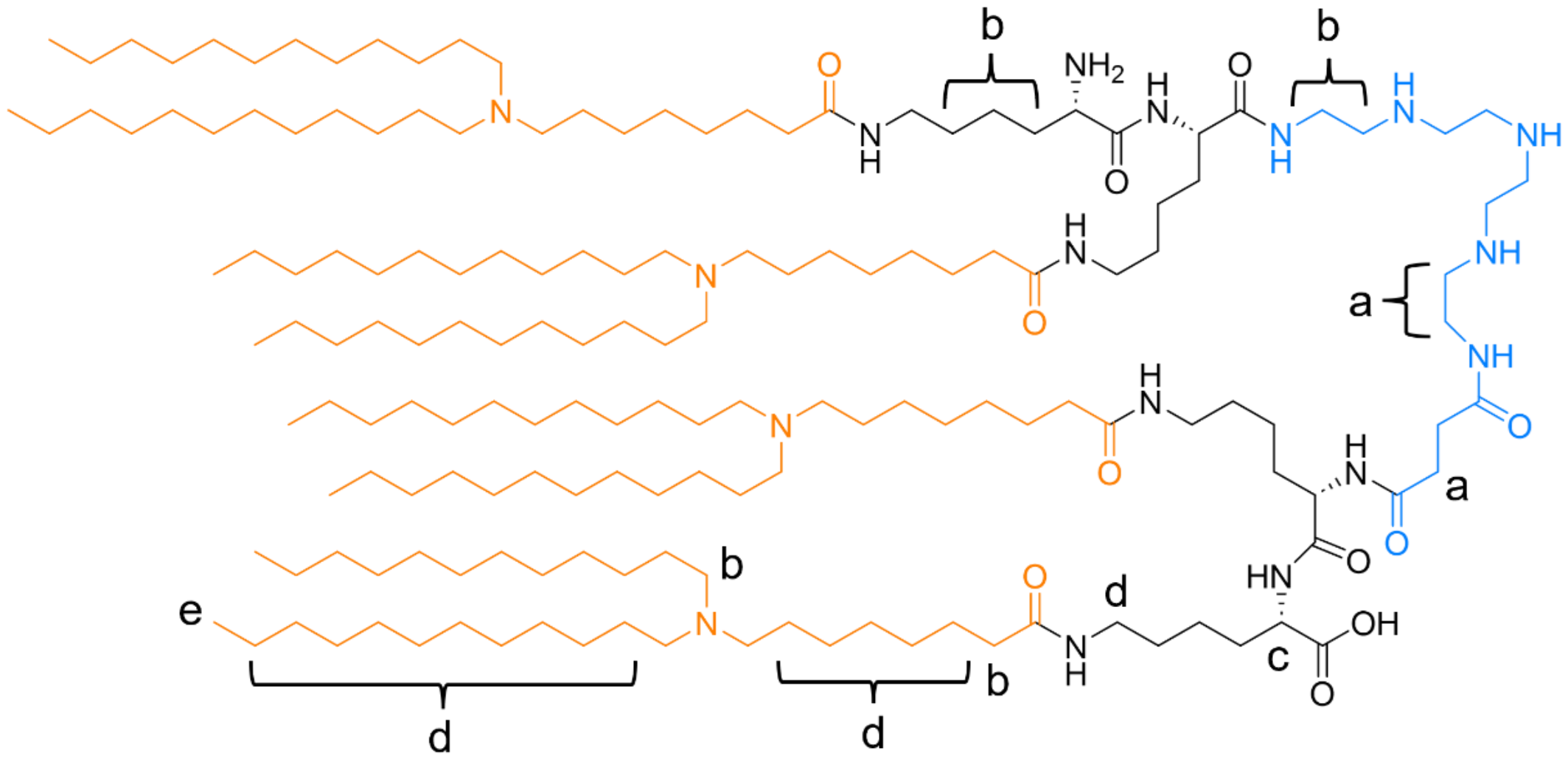

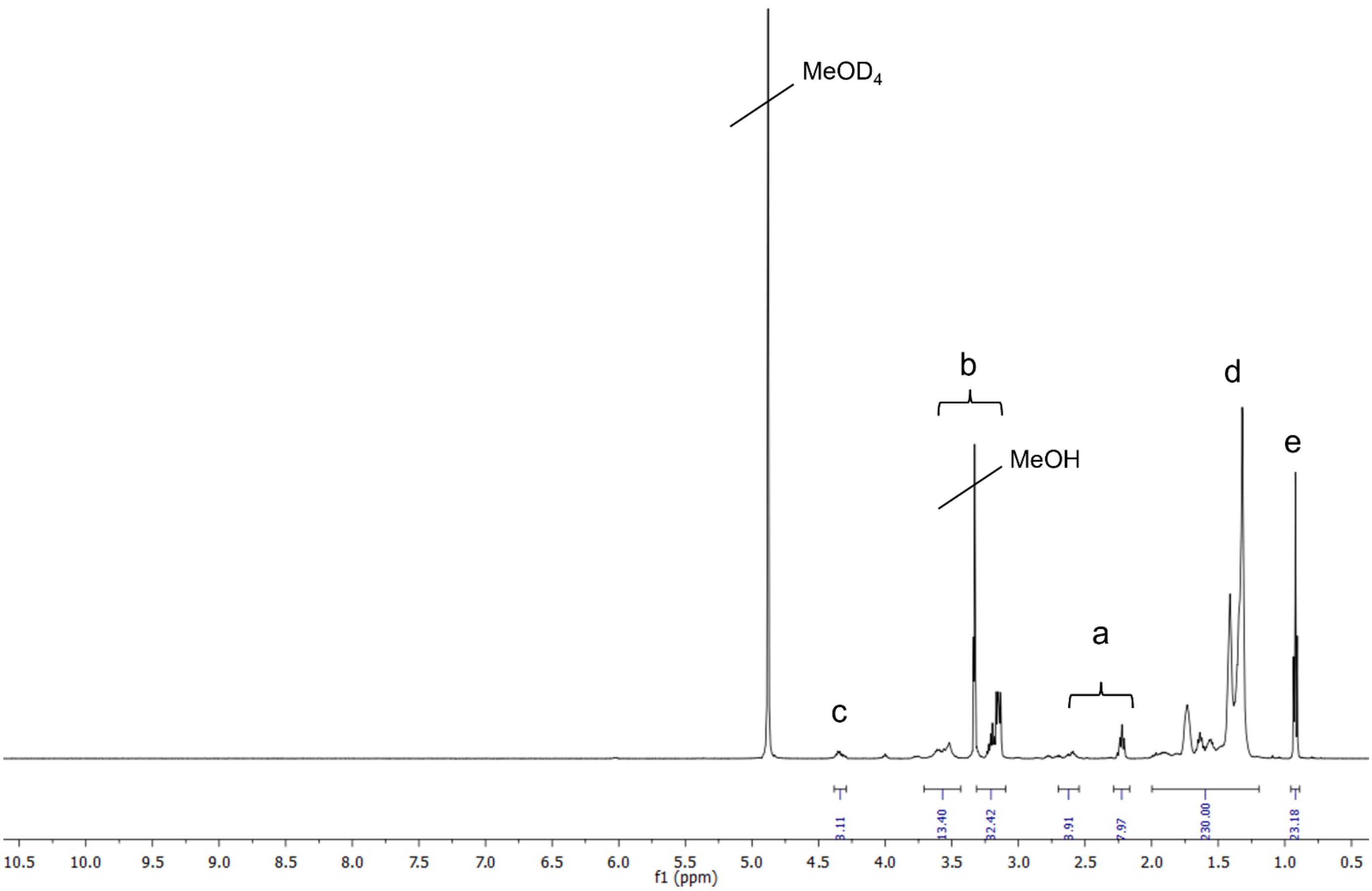

^{1}H-NMR (400 MHz, methanol-d4) δ (ppm) = 0.888 – 0.952 (t, 24H, -CH_3, dodecanal), 1.278 – 1.791 (m, 230H, βγδ H lysine, βγδεz H Oc, -CH_2- dodecanal), 2.166 – 2.545 (m, 8H, -NH-CH_2-

CH_2-NH- Stp, 4H, -CO-CH_2-CH_2-CO Stp), 3.096 – 3.707 (m, 46H, -CH_2-N- LAF, α H Oc, ε H lysine, -CO-NH-CH_2-CH_2-NH- Stp), 4.291 – 4.383 (m, 3H, α H amino acids).

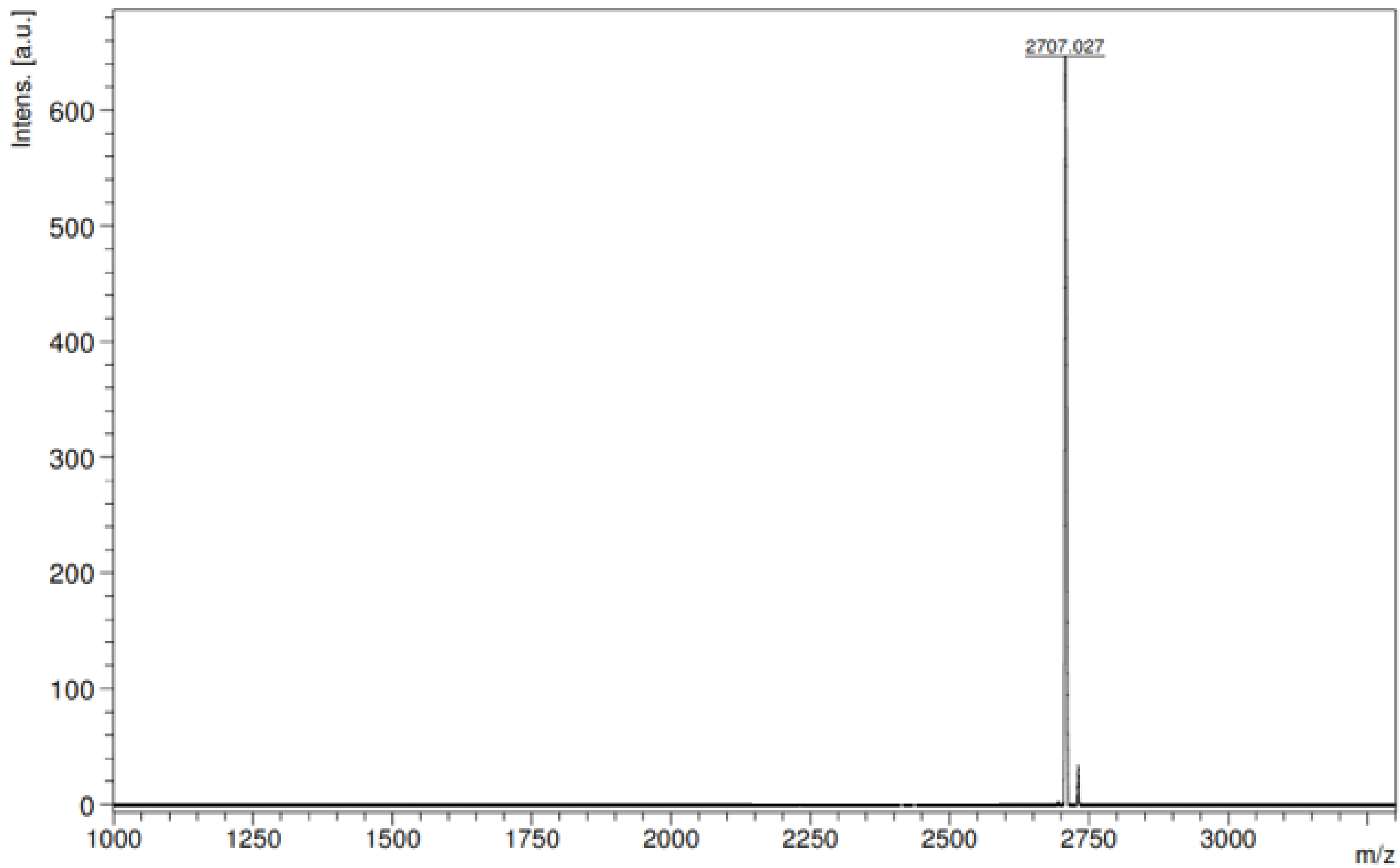

MALDI-TOF MS: calculated for $C_{164}H_{327}N_{17}O_{11}$ = 2713.53 found: $[M+H]^+$ = 2707.027

1960: **12Oc-O-U1-1:4**

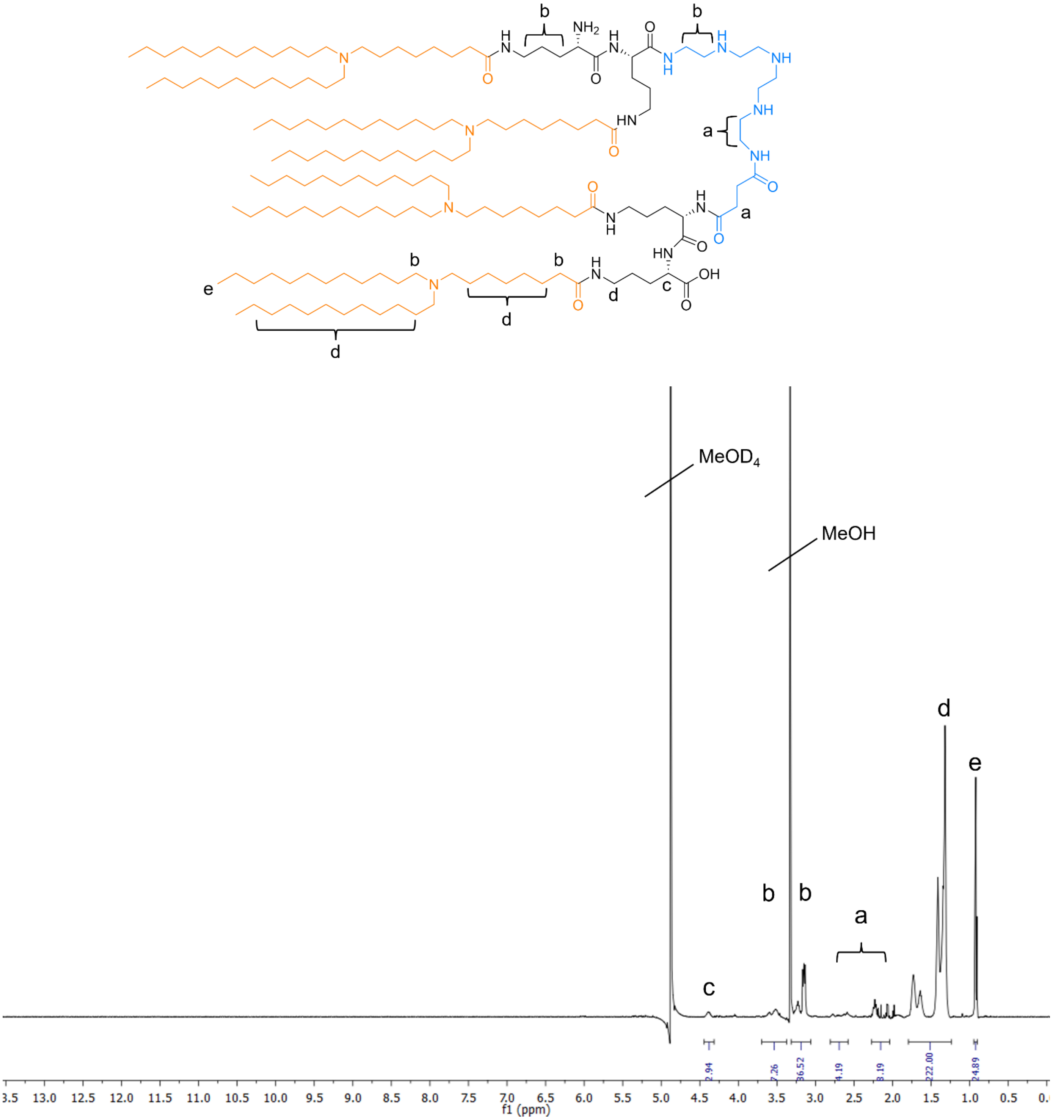

^{1}H-NMR (400 MHz, methanol-d4) δ (ppm) = 0.9 – 0.945 (t, 24H, $-CH_3$, dodecanal), 1.195 – 1.998 (m, 222H, βγ H ornithine, βγδεz H Oc, $-CH_2-$ dodecanal), 2.03 – 2.808 (m, 8H, $-NH-CH_2-CH_2-NH-$ Stp, 4H, $-CO-CH_2-CH_2-CO$ Stp), 3.061 – 3.698 (m, 46H, $-CH_2-N-$ LAF, α H Oc, δ H ornithine, $-CO-NH-CH_2-CH_2-NH-$ Stp), 4.315 – 4.444 (m, 3H, α H amino acids).

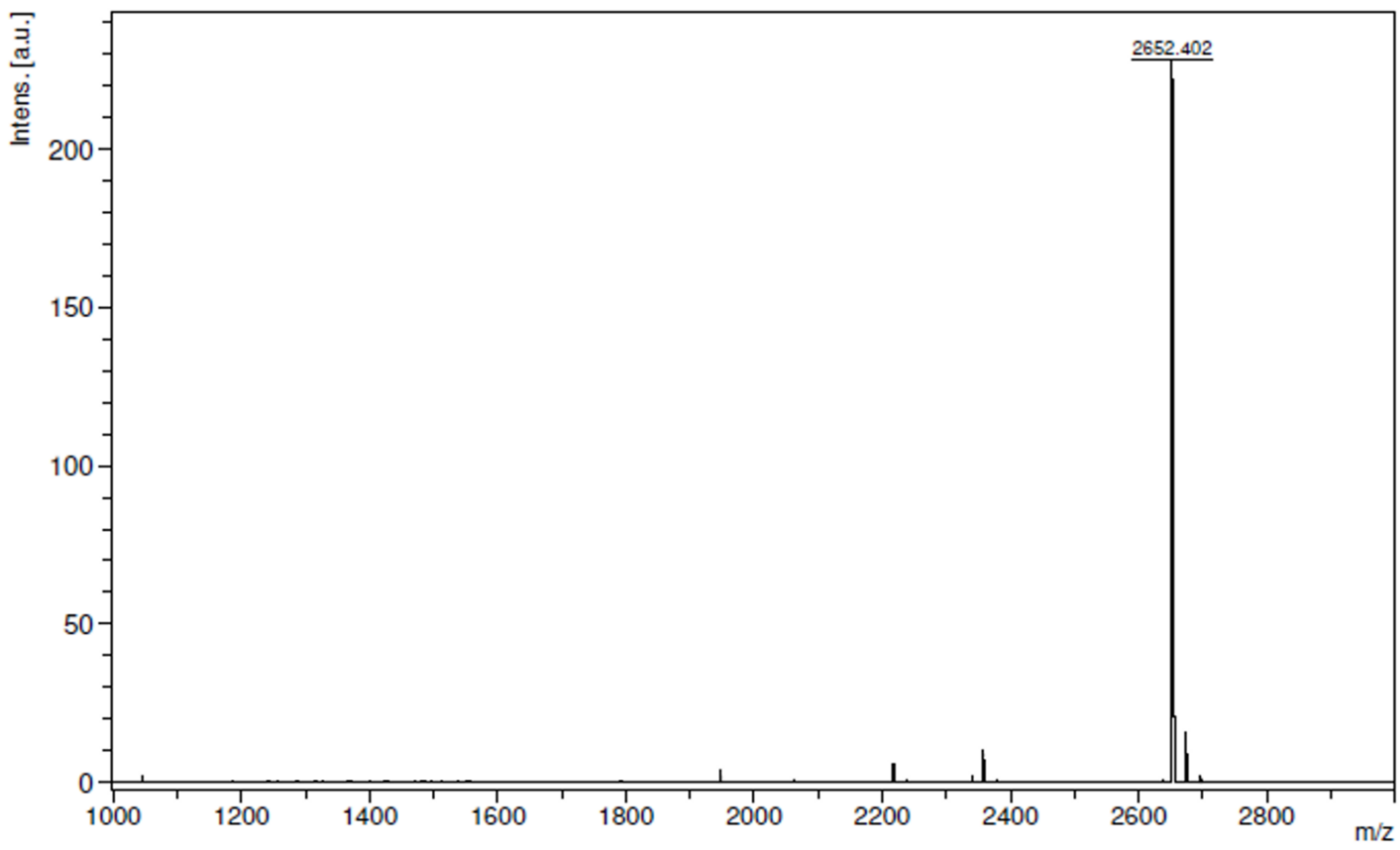

MALDI-TOF MS: calculated for $C_{160}H_{319}N_{17}O_{11}$ = 2657.42 found: $[M+H]^+$ = 2652.402

1621: **8Oc-K-B2-1:4**

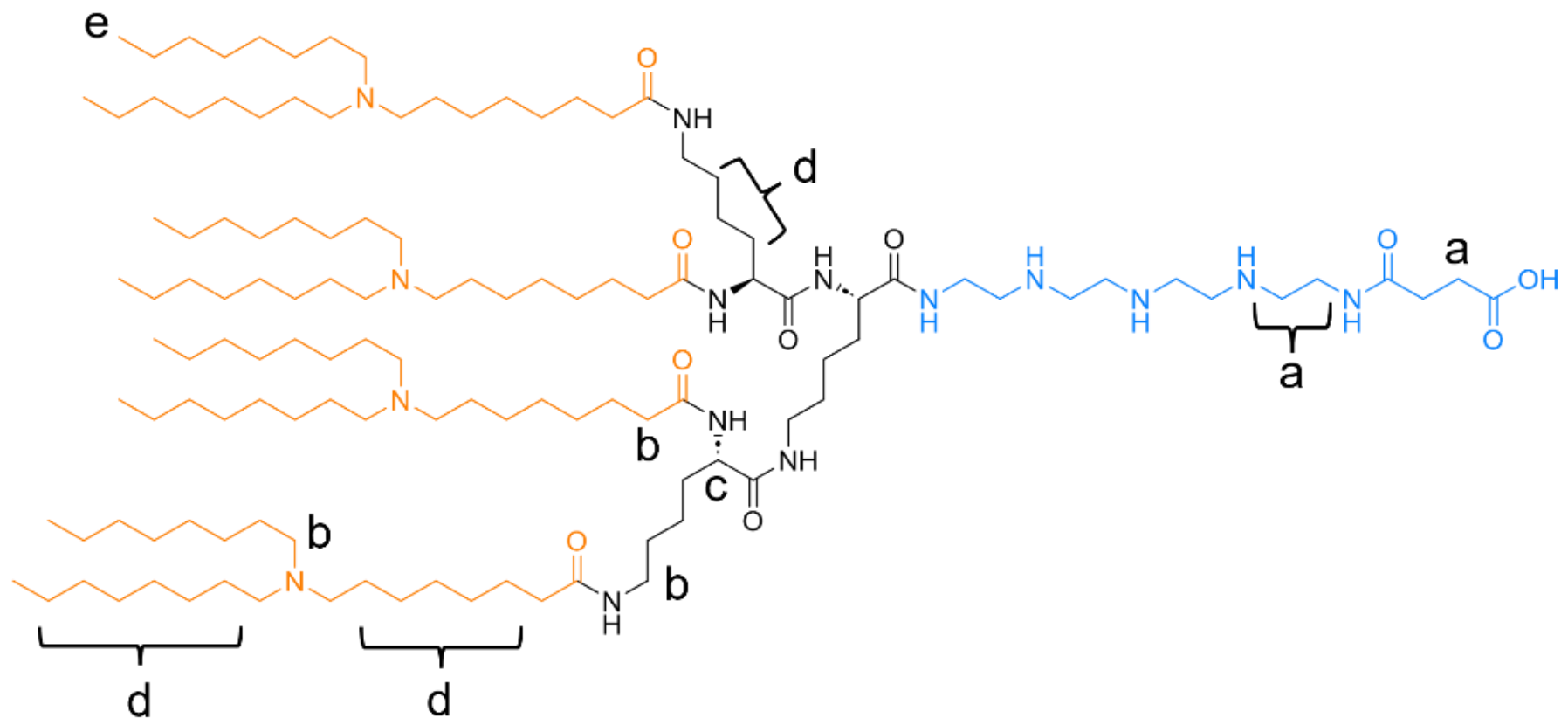

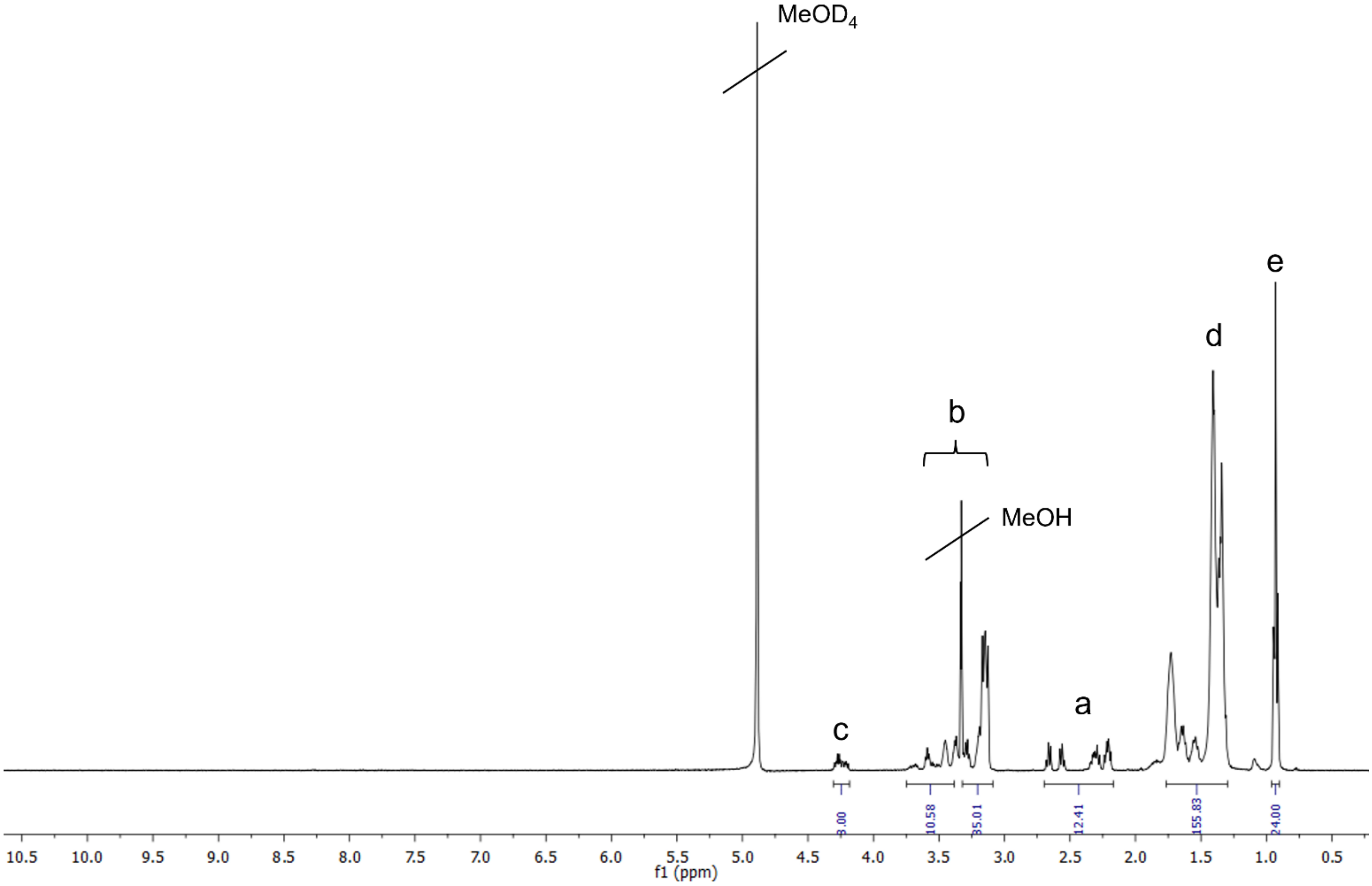

^{1}H-NMR (400 MHz, methanol-d4) δ (ppm) = 0.88 – 0.98 (t, 24H, $-CH_3$, octanal), 1.241 – 1.904 (m, 154H, βγδ H lysine, βγδεz H Oc, $-CH_2-$ octanal), 2.159 – 2.694 (m, 8H, $-NH-CH_2-CH_2-NH-$ Stp, 4H, $-CO-CH_2-CH_2-CO$ Stp), 3.008 – 3.497 (m, 46H, $-CH_2-N-$ LAF, α H Oc, ε H lysine, $-CO-NH-CH_2-CH_2-NH-$ Stp), 4.171 – 4.322 (m, 3H, α H amino acids).

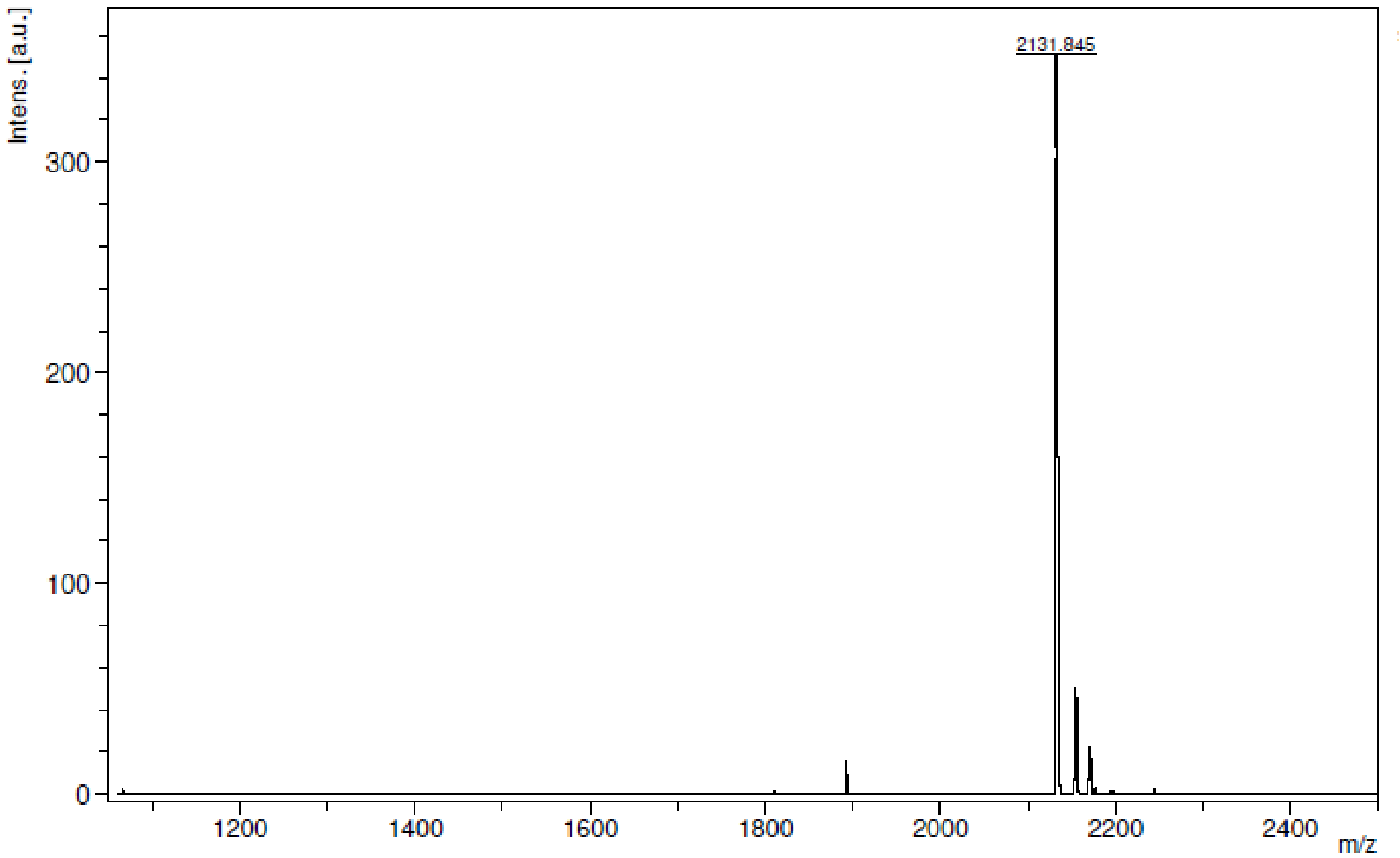

MALDI-TOF MS: calculated for $C_{126}H_{251}N_{15}O_{10}$ = 2136.49 found: $[M+H]^+$ = 2131.845

1813: **8Oc-O-B2-1:4**

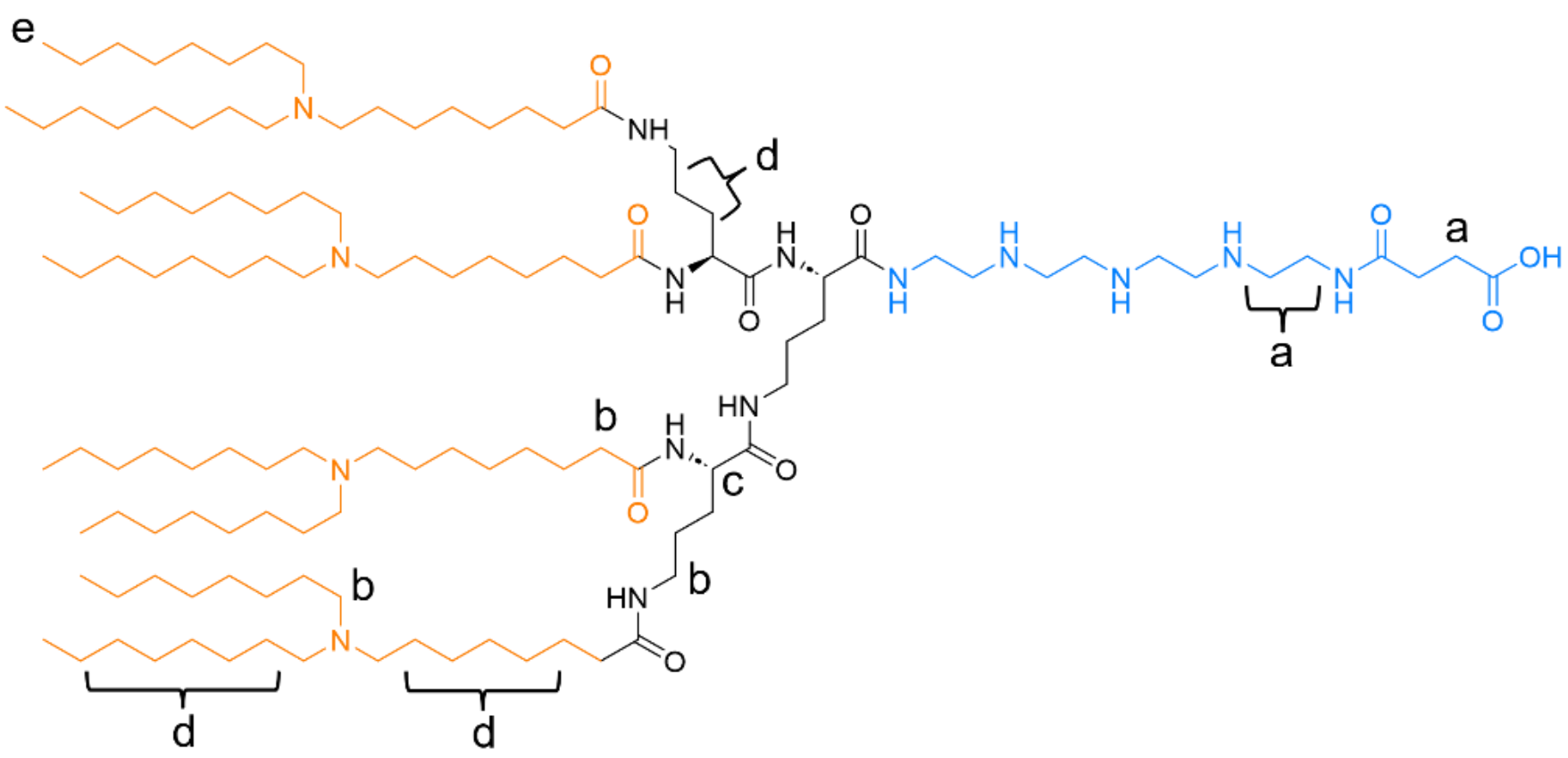

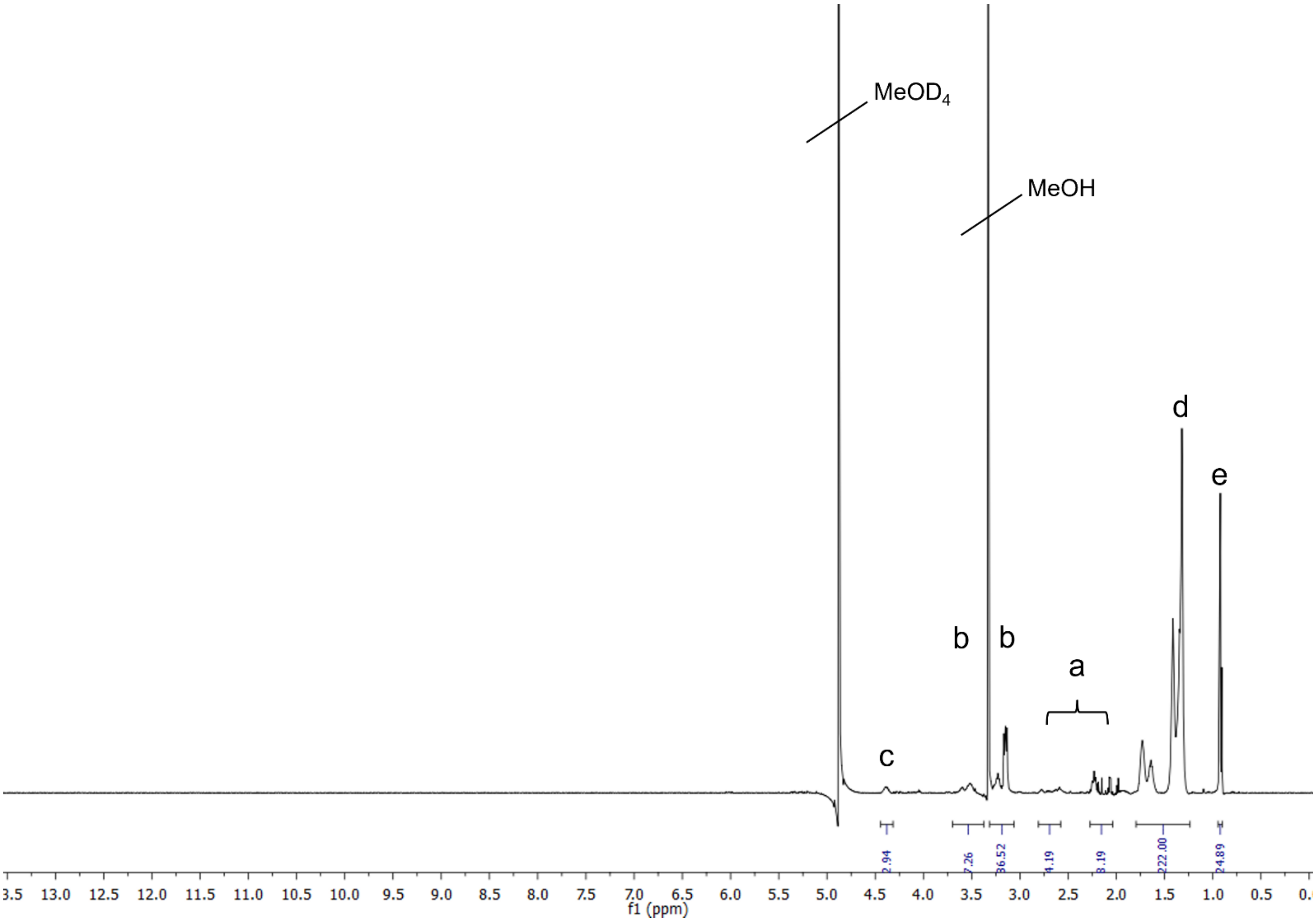

^{1}H-NMR (400 MHz, methanol-d4) δ (ppm) = 0.89 – 0.974 (t, 24H, -CH$_3$, octanal), 1.278 – 1.791 (m, 148H, βγ H ornithine, βγδεz H Oc, -CH$_2$- octanal), 2.186 – 2.699 (m, 8H, -NH-CH$_2$-CH$_2$-NH- Stp, 4H, -CO-CH$_2$-CH$_2$-CO Stp), 3.082 – 3.638 (m, 46H, -CH$_2$-N- LAF, α H Oc, δ H ornithine, -CO-NH-CH$_2$-CH$_2$-NH- Stp), 4.104 – 4.244 (m, 3H, α H amino acids).

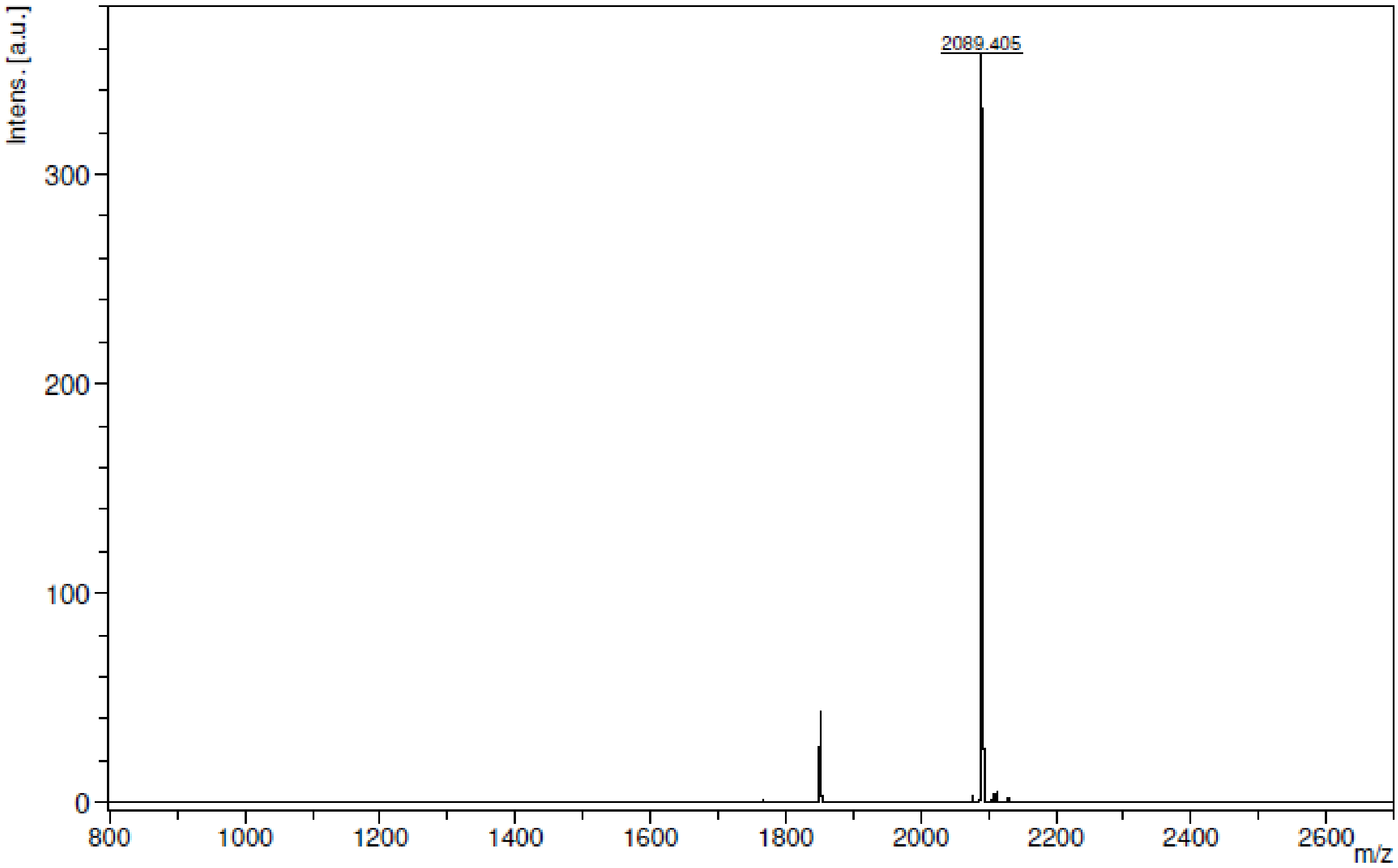

MALDI-TOF MS: calculated for $C_{123}H_{245}N_{15}O_{10}$ = 2094.41 found: $[M+H]^+$ = 2089.405

1752: **12Bu-K-B2-1:4**

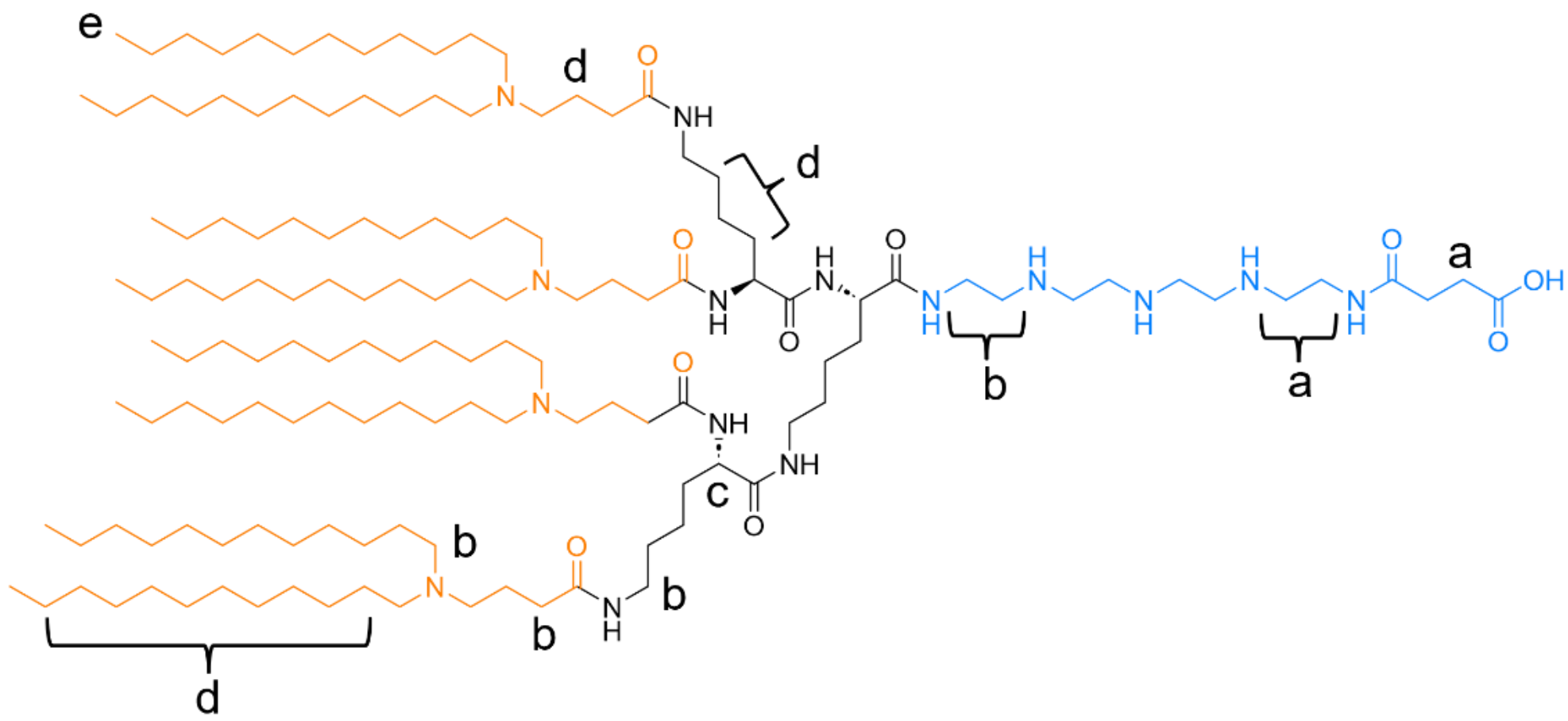

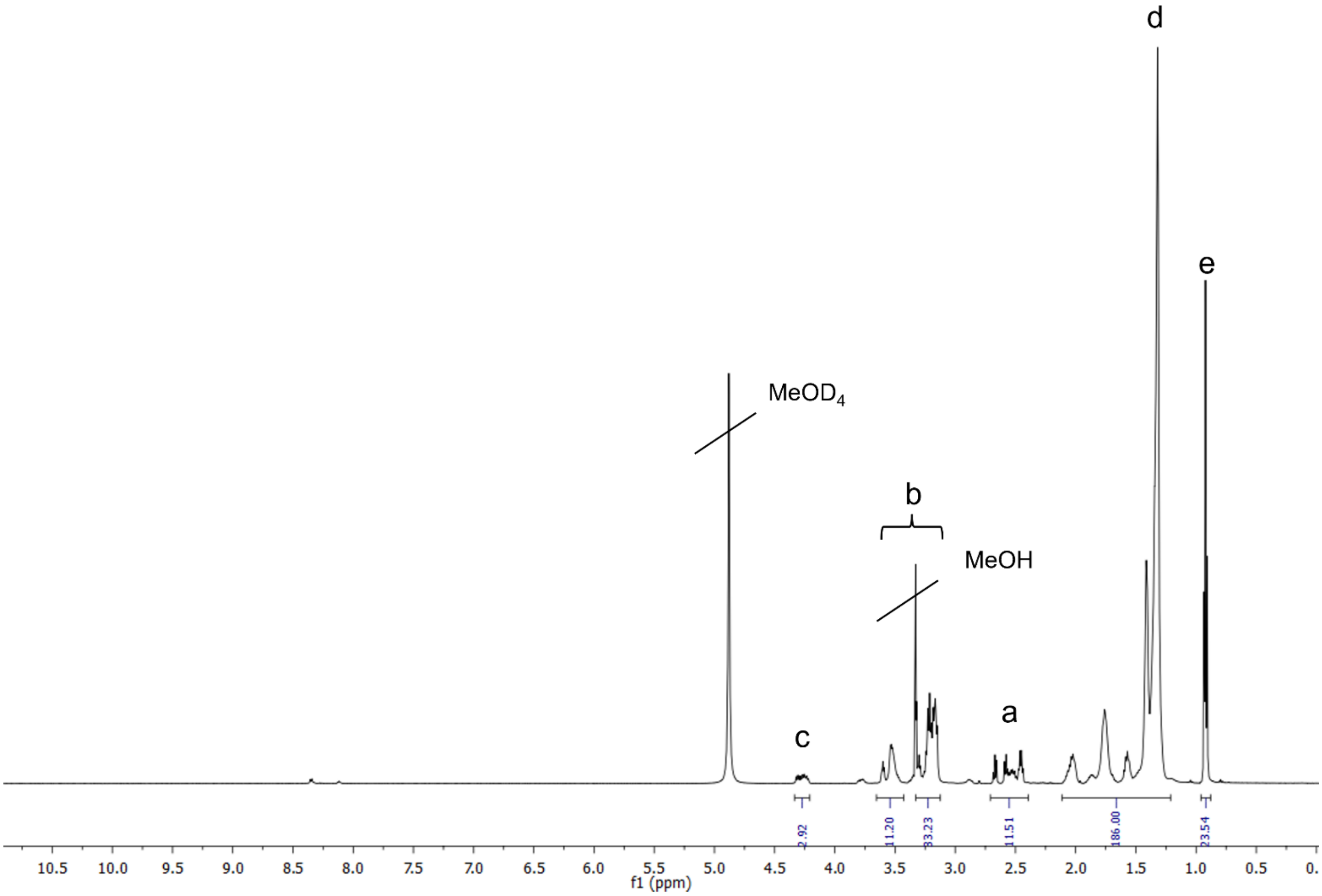

^{1}H-NMR (400 MHz, methanol-d4) δ (ppm) = 0.892 – 0.952 (t, 24H, $-CH_3$, dodecanal), 1.236 – 2.1 (m, 186H, βγδ H lysine, βγ H Bu, $-CH_2-$ dodecanal), 2.396 – 2.707 (m, 8H, $-NH-CH_2-CH_2-NH-$ Stp, 4H, $-CO-CH_2-CH_2-CO$ Stp), 3.139 – 3.598 (m, 46H, $-CH_2-N-$ LAF, α H Bu, ε H lysine, $-CO-NH-CH_2-CH_2-NH-$ Stp), 4.204 – 4.33 (m, 3H, α H amino acids).

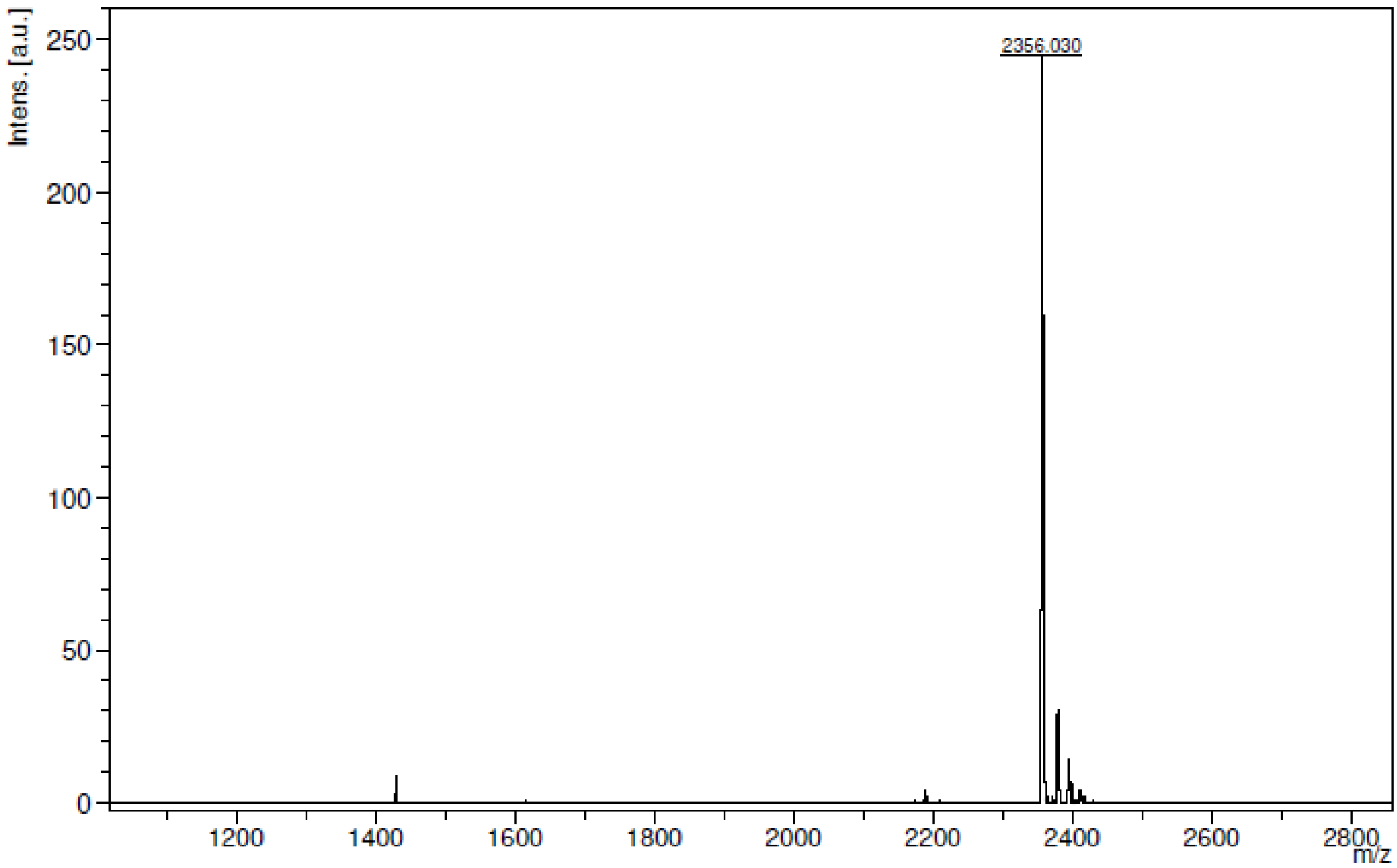

MALDI-TOF MS: calculated for $C_{142}H_{283}N_{15}O_{10}$ = 2360.92 found: $[M+H]^{+}$ = 2356.030

1814: **12Bu-O-B2-1:4**

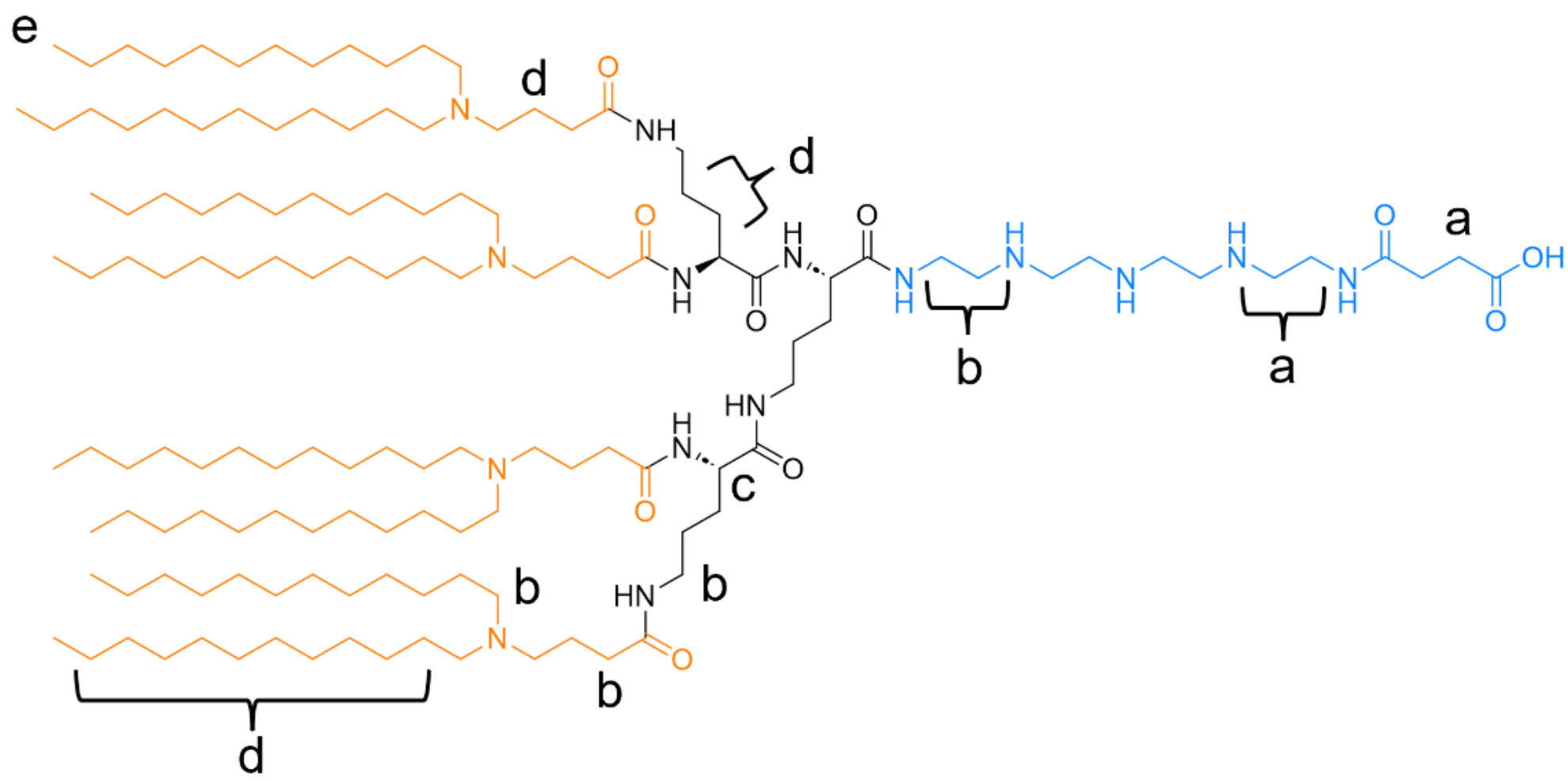

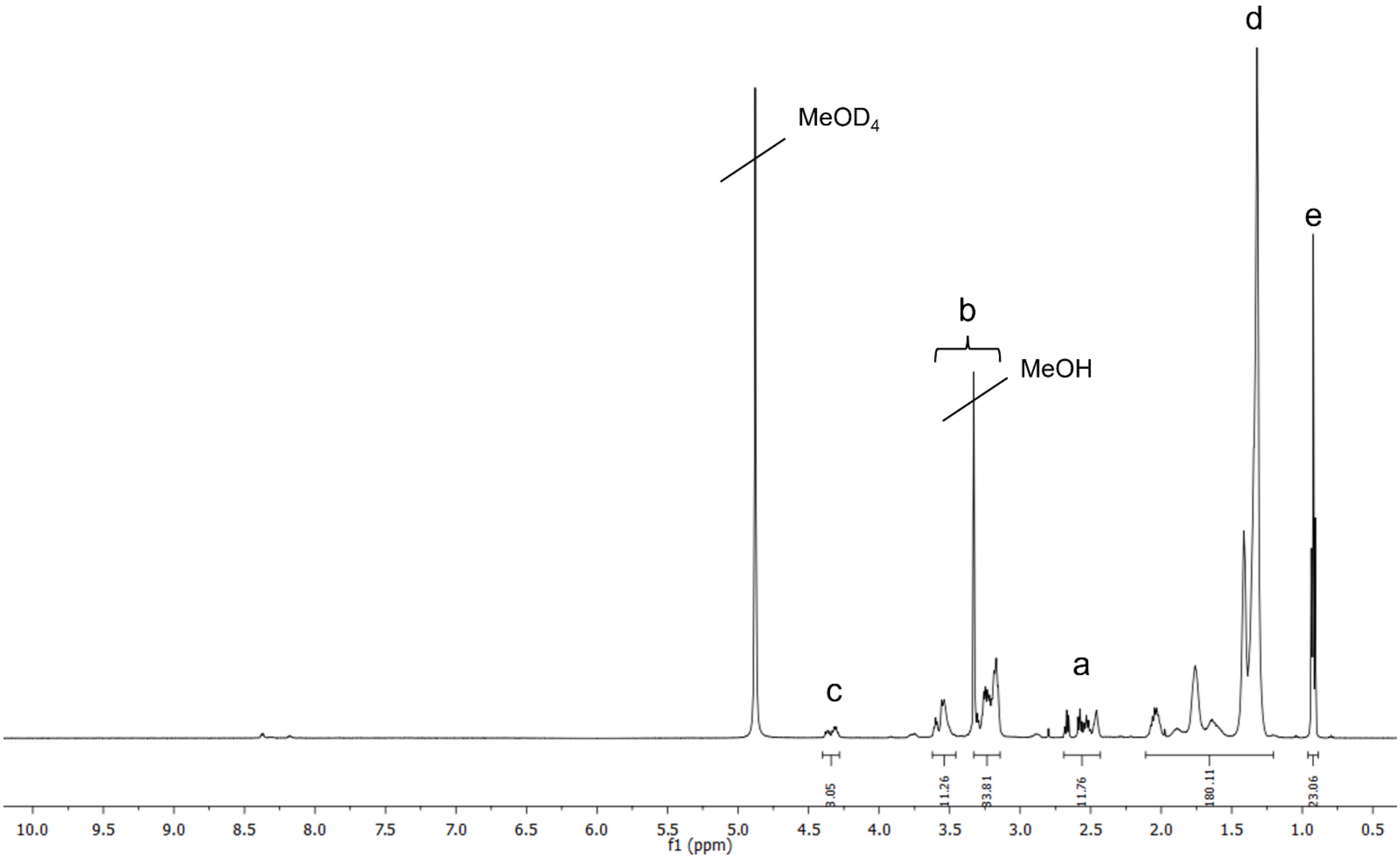

^{1}H-NMR (400 MHz, methanol-d4) δ (ppm) = 0.888 – 0.962 (t, 24H, $-CH_3$, dodecanal), 1.204 – 2.11 (m, 180H, βγ H ornithine, βγ H Bu, $-CH_2-$ dodecanal), 2.433 – 2.69 (m, 8H, $-NH-CH_2-CH_2-NH-$ Stp, 4H, $-CO-CH_2-CH_2-CO$ Stp), 3.09 – 3.633 (m, 46H, $-CH_2-N-$ LAF, α H Bu, δ H ornithine, $-CO-NH-CH_2-CH_2-NH-$ Stp), 4.28 – 4.39 (m, 3H, α H amino acids).

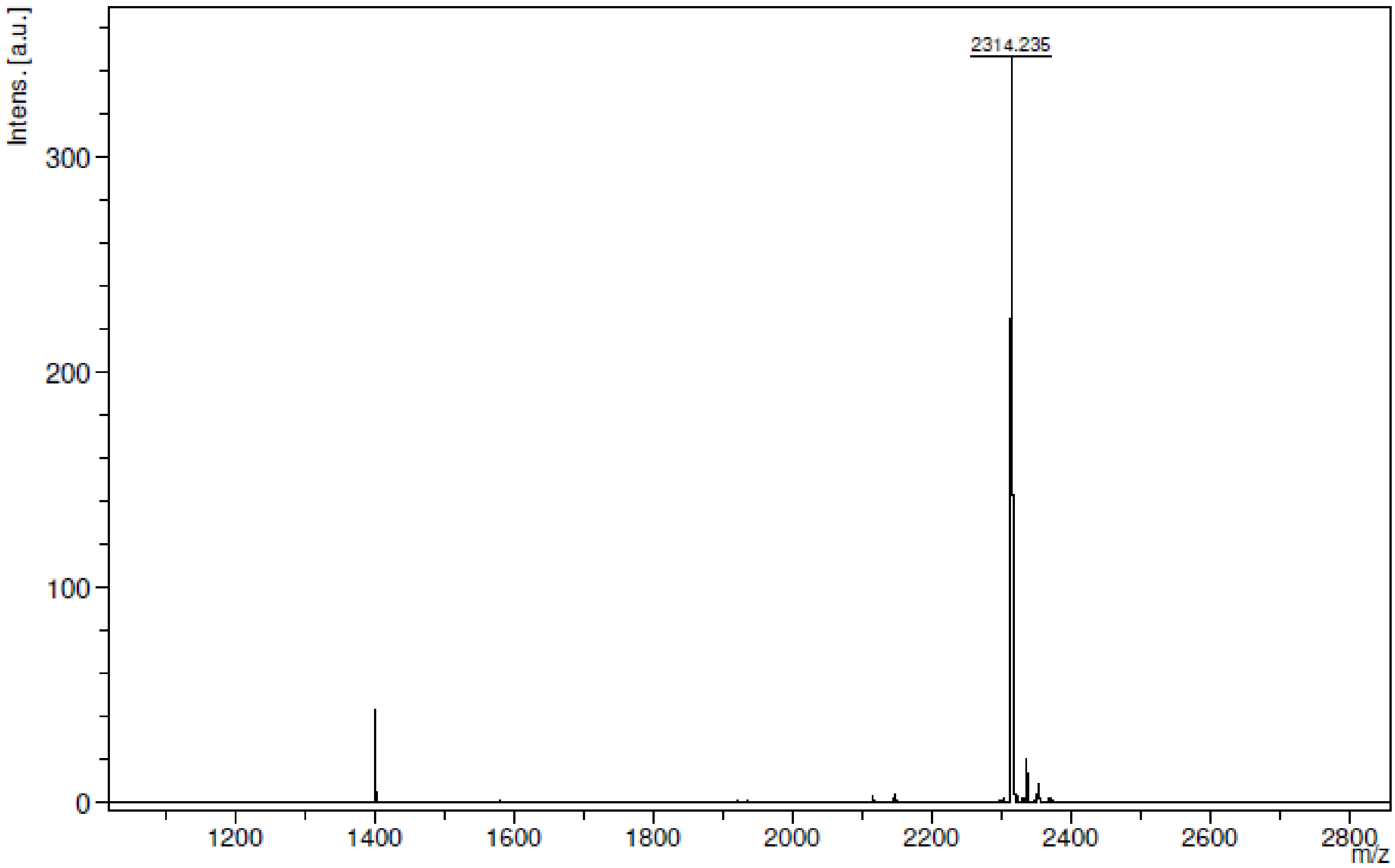

MALDI-TOF MS: calculated for $C_{139}H_{277}N_{15}O_{10}$ = 2318.84 found: $[M+H]^+$ = 2314.236

Determination of the octanol/water partition coefficient (logD)

Selected LAF-Stp XPs were synthesized as described in the section regarding solid phase assisted peptide synthesis. An additional C-terminal tyrosine was incorporated to enable UV–vis detection (wavelength λ = 280 nm). A standard curve of each carrier was prepared with concentrations ranging from 0.1 to 1 mg mL^{-1} using 1-octanol to prepare the dilutions and the blank. Samples were prepared by diluting a 10 mg mL^{-1} stock solution 1:10 (v/v) with 20 mm HEPES buffer at physiological (7.4) and endosomal (6.5, 5.5) pH values. After adding an equal volume of 1-octanol, the mixture was incubated under constant shaking (45 rpm) at RT for 24 h. Afterwards, samples were centrifuged (4000 rpm, 20 °C, 4 min) and stored at 4 °C for 1 h to allow phase separation. At acidic pH, all XPs align towards the interphase between water and octanol. Albeit displaying a separate layer, the interphase was considered as a component of the aqueous phase. Due to the occurrence of opalescence in the inter- and aqueous phase, only the hydrophobic layer (1-octanol) phase was measured at wavelength λ = 280 nm against 1-octanol as blank using a Cary 3500 UV–vis Multicell Peltier spectrophotometer (Agilent Technologies, Waldbronn, Germany). The calculated carrier concentration in 1-octanol was subtracted from 1 mg mL^{-1} to obtain the concentrations in the combined inter- and aqueous layer. logD values were calculated as log($c_{octanol}$ / c_{water}).

Formation of LAF-Stp XP or succPEI polyplexes and D-Lin-MC3-DMA based lipid nanoparticles (LNPs)

For LAF-Stp XP polyplex formation, CleanCap® FLuc mRNA (5moU) (Trilink Biotechnologies, San Diego, CA, USA) was diluted in HBG (HEPES buffered glucose, 20 mM HEPES, 5% glucose (w/v), pH 7.4). The calculated amount of LAF-Stp carrier at indicated N/P (nitrogen/phosphate) ratio was diluted in sterile Millipore water from a stock solution (10 mg ml^{-1} in EtOH/H2O at specific solvent ratios). For N/P ratio calculation all secondary amines of the Stp unit, terminal primary amines and tertiary amines of the LAF domains were considered. Equal volumes of mRNA and LAF-Stp carrier dilutions were mixed by rapid pipetting and incubated at RT for 40 min. The final mRNA concentration was 12.5 µg mL^{-1}, unless stated otherwise. For succPEI polyplexes, indicated amounts of nucleic acid were diluted in HBG and mixed with succPEI at a weight to weight (w/w mRNA to succPEI) ratio of 4 dissolved in the same volume of HBG.

D-Lin-MC3-DMA based mRNA LNPs were formed using the ethanol-dilution method in a self-assembly process. Initially, D-Lin-MC3-DMA, cholesterol 1,2-distearoyl-sn-glycero-3-phosphocholine (DSPC) and DMG-PEG2000 were dissolved in EtOH at molar ratios of D-Lin-MC3-DMA /cholesterol/DSPC/DMG-PEG2000 38.5/50/10/1.5 mol%. LNPs were formed at N/P

ratio 3. FLuc mRNA was diluted in citrate buffer (10 mM, pH 4.0). Subsequently, the ethanolic and aqueous phase were mixed by rapid pipetting (1:3 v/v). After incubation at RT for 10 min, the formulated LNPs were diluted with HBG to a final EtOH concentration below 5% and mRNA concentration of 3 µg mL^{-1}.

Formation of LAF-Stp XP LUVs and cholesterol/DOPC containing bulk phases

For self-assembly studies, each LAF-Stp XP was rehydrated in 20 mM HEPES buffer at neutral (7.4) and acidic (6.5, 5.5) pH at a XP concentration of 20 mg mL^{-1}. The ionic strength of the HEPES buffer varied with pH but remained below 0.02 M across the range of conditions used. Samples were vortexed at RT for 2 min to ensure a homogeneous distribution of the carrier molecules within the dispersion. All XPs formed stable, slightly turbid dispersions.

LAF-Stp XP/chol and LAF-Stp XP/chol/DOPC bulk phases were prepared at a range of pH conditions via three dialysis steps[3]. Initially, the respective XP, Chol and potentially DOPC were dissolved in ethanol and mixed in a molar ratio of 1:1 (LAF-Stp XP:chol) and 1:1:1 (LAF-XP:chol:DOPC) to a total lipid concentration of 56.1 mg/mL. The ethanol mixture was introduced into a dialysis cup with a molecular weight cutoff of 3.5 kDa (Slide-A- Lyzer™ MINI dialysis device, Thermo Scientific) and 50 mM citrate buffer (pH 3) was rapidly added to the cup at a volume ratio 1:3 buffer:ethanol. The dialysis cup was placed into a beaker containing 500× the volume of dialysis buffer at 22 ± 2 °C. Samples were first dialyzed against 50 mM citrate buffer (pH 3) containing ethanol (at a volume ratio of 3:1, buffer: ethanol) for 48 h. In the second step, samples were dialyzed against PBS (1 mM KH2PO4, 155 mM NaCl, and 3 mM Na2HPO4 0.7H2O, pH 7.4) for 48 h. In the third step, the samples were dialyzed against HBG (HEPES buffered glucose, 20 mM HEPES, 5% glucose (w/v)) with the respective final pH (4.3, 4.6, 5.3, 6, 6.9 and 7.3) for 48 h. The ionic strength of HBG was below 0.02 M for all pH conditions. During the dialysis process, large precipitates were formed from the carriers and cholesterol, which were extracted for characterization by SAXS.

Particle size and zeta potential

Dynamic and electrophoretic laser light scattering (DLS, ELS) measurements were performed using a Zetasizer Nano ZS (Malvern Instruments, Malvern, Worcestershire, United Kingdom) and a folded capillary cell (DTS1070). In terms of particle size and polydispersity index (PDI), 80 µL of mRNA-polyplex dispersion were formulated as described above. The following settings were conducted: 30 s of equilibration time, temperature of 25 °C, refractive index 1.330, viscosity 0.8872 mPa s. Samples were measured three times with six sub-runs per measurement. For zeta potential measurements, samples were diluted with HBG to a final volume of 800 µL immediately prior to measurement. The equilibration time was extended to

60 s. All other measurement parameters remained unchanged and were identical to those used for size determination. Three measurements with 15 sub-runs were conducted and zeta potential was calculated by Smoluchowski equation. To examine storage stability and batch-to-batch reproducibility, two independently formed polyplex batches were either evaluated immediately upon incubation or after 7 days of storage at 4 °C. Intensity-size distribution plots were proposed for all samples to assess reproducibility between batches and detect any changes in particle size over time. LUV samples were prepared by diluting the respective XP stock solution (10 mg mL^{-1}) in 20 mM HEPES buffer at cytosolic (7.4) and endosomal (6.5, 5.5) pH to reach a final concentration of 0.25 mg mL^{-1}. Size measurements were conducted in equal fashion to mRNA polyplexes.

Encapsulation efficiency of mRNA polyplexes

The encapsulation efficiency of mRNA polyplexes assembled with different Bundle and U-shape LAF-Stp XPs was determined using a Quant-iT™ RiboGreen RNA Assay-Kit (Thermo Fisher Scientific). Polyplexes were prepared as described above at an mRNA concentration of 12.5 µg mL^{-1}. The samples were then diluted with 1x TE (10 mM Tris-HCl, 1 mM EDTA, pH 7.5 in RNase-free water) to an mRNA concentration of 2 µg mL^{-1}. An aliquot of 50 µL was either mixed with 50 µL of 1x TE as untreated control or with 50 µL of 1x TE containing 2% (v/v) TritonX-100 and 250 I.U. mL^{-1} heparin as treated sample, which represents fully disassembled polyplexes. The samples were incubated under constant shaking at 37 °C for 10 min. After cooling down at RT for 5 min, 100 µL of a 200-fold dilution RiboGreen reagent in 1x TE were added. The fluorescence intensity was measured with a Tecan microplate reader (Spark, Tecan, Männersdorf, Switzerland) at excitation/emission wavelength of 485/535 nm. All samples were prepared in technical duplicates. Pure HBG in 1x TE or 1x TE with TritonX-100 and heparin were measured as background signals. The encapsulation efficiency was calculated after background subtraction according to the following equation:

$$ee\ [\%] = 100\% - \frac{mean\ emission\ (untreated\ sample)}{mean\ emission\ (treated\ sample)}\ x\ 100\%$$

Transmission electron microscopy (TEM)

LAF-Stp XP containing polyplexes were formed as described above at an mRNA concentration of 50 µg mL^{-1}. After incubation at RT for 40 min, polyplexes were diluted with equal volumes of 20 mM HEPES buffer at pH 7.4 or pH 4.0 to reach a final mRNA concentration of 25 µg mL^{-1} and pH's of 7.4 and 5.5, respectively. Subsequently, mild plasma cleaning was used to activate the formvar/carbon-coated 300 mesh copper grids (Ted Pella Inc., Redding, CA,

USA). Afterwards, 10 µL of the polyplex solution was placed on the grids and incubated for 2.5 min. Excess liquid was removed with filter paper. Next, the grids were washed with 5 µL of staining solution for 5 s and incubated with 5 µL of a 2% (w/v) aqueous uranylformate solution for another 5 s, followed by air-drying for 10 min. Samples characterization was carried out at 80 kV using a JEM-1011 electron microscope (JEOL, Tokyo, Japan). Images were analyzed using ImageJ 1.54g (Java 1.8.0_345).

Cell culture

Human adherent cervix carcinoma cell line (HeLa), HeLa-Gal8-mRuby stably expressing a galectin8-mRuby3 fusion protein [4,5] and murine neuroblastoma cell line Neuro2a (N2a) cells were cultured in Dulbecco's modified Eagle's medium (DMEM)-low glucose (1 g L^{-1} glucose) supplemented with 10% (v/v) FBS, 4 mM of stable glutamine, 100 U mL^{-1} of penicillin, and 100 µg mL^{-1} of streptomycin. The cells were cultured at 37 °C, 5% CO_2 and relative humidity of 95%.

Transfection

Either 10,000 N2a or 5,000 HeLa cells were seeded in 96-well plates 24 h before the transfection. Prior to treatment, the cell culture medium was replaced with 98.8 µL fresh medium for polyplexes and 95 µL for LNPs. Transfection efficacy was evaluated for a dose of 15 ng mRNA per well. Hence, 1.2 µL LAF-Stp XP or succPEI polyplex solution containing 12.5 µg mL^{-1} or 5 µL LNP solution containing 3 µg mL^{-1} Luc mRNA were transferred to the corresponding wells in biological triplicates, ensuring a final volume of 100 µL.

Luciferase expression assay

Transfection efficacy was evaluated after incubating the cells at 37 °C for 24 h. The medium was removed, and cells were treated with 100 µL of 0.5× lysis buffer, and frozen at −80 °C at least overnight. Before measuring the luciferase activity, the plates were thawed for 1 h at RT. The cell lysate was diluted in PBS at a ratio of 1:100. Thirty-five µL of cell lysate dilution were measured using a Centro LB 960 plate reader luminometer (Berthold Technologies GmbH & Co. KG, Bad Wildbad, Germany) for 10 s after addition of 100 µL LAR buffer (20 mM glycylglycine; 1 mM $MgCl_2$; 0.1 mM ethylenediaminetetraacetic acid; 3.3 mM dithiothreitol; 0.55 mM adenosine 5′-triphosphate; 0.27 mM coenzyme A, pH 8–8.5) supplemented with 5% (v/v) of a mixture of 10 mM luciferin and 29 mM glycylglycine. Transfection efficacy was

calculated for the seeded number of cells and presented as relative light units (RLU) per well. Data was presented after background (HBG-treated cells) subtraction (mean ± SD, n=3, biological replicates).

Relative metabolic activity assessed with MTT assay

Transfections were performed as described above. Briefly, 10 µL MTT (5 mg mL^{-1}) were added to each well 24 h after transfection, reaching a final concentration of 0.5 mg mL^{-1}. After incubation at 37 °C for 2 h, the supernatant was removed. The plates were stored at -80 °C for at least 1 h. Next, 100 µL of dimethylsulfoxide (DMSO) were added to each well to dissolve the purple formazan product. After incubation under constant shaking (250 rpm) for 30 min at 37 °C, quantification was carried out photometrically in biological triplicates, using a Tecan microplate reader (Spectrafluor Plus, Tecan, Männedorf, Switzerland). The absorbance was measured at 590 nm with background correction at 630 nm. Relative metabolic activity related to control wells (HBG-treated cells) was calculated by the equation: $\frac{[A]sample}{[A]control}\ x\ 100\%$

Inhibition of endosomal acidification assessed with bafilomycin A1 assay

The impact of inhibiting the endosomal vATPase on transfection efficacy was evaluated by performing a bafilomycin A1 (BafA1) assay. Twenty-four h prior to transfection, 10,000 N2a cells were seeded into 96 well plates. Thirty min before transfection, the culture medium was replaced either with fresh medium or medium supplemented with BafA1 (stock solution: 0.1 µg mL^{-1} in DMSO) to reach a final concentration of 200 nM BafA1 per well upon LAF-XP polyplex addition. Polyplexes were formed as described above and transfected at a dose of 15 ng mRNA per well. After polyplex addition, cells were incubated for 4 h in the continued absence or presence of BafA1 without further washing steps. This incubation time was chosen to avoid possible toxicity occurring from BafA1. Afterwards, cells were lysed and read-out performed via a luciferase expression assay as described above. All samples were carried out in biological triplicates.

To clarify whether bafilomycin has an effect on cellular uptake, N2a cells (10,000 cells per well) were seeded in 96-well plates 24 h before transfection. Thirty min prior to treatment, the culture medium was replaced with fresh medium or medium containing BafA1 (stock solution: 0.1 µg mL^{-1} in DMSO). The final concentration of BafA1 was 200 nM per well. Polyplexes were formed as described above at an mRNA concentration of 12.5 µg mL-1, containing 80% (w/w) mRNA-Luc and 20% (w/w) mRNA-Cy5-FLuc and transfected at an mRNA dose of 15 ng per well. The cells were incubated for 2 h. Afterwards, the medium was removed, 80 µL of cold heparin

solution (1000 I.U. / mL in PBS) were added, followed by 15 min of incubation. Subsequently, the cells were washed once with PBS and trypsinized (1× trypsin/EDTA, 30 µL) for 5 min at 37 °C. Next, the cell suspension was resuspended with 90 µL of PBS containing 10% FBS and supplemented with DAPI (1 µg/mL) and transferred to flat bottom FACS plates. Cells were analyzed by flow cytometry using a CytoFLEX S Flow Cytometer (Beckman Coulter, Brea, CA, USA) over 60 seconds at a flow rate of 170 µL min^{-1}. Flow cytometry data were analyzed using FlowJo™ v10.8 flow cytometric analysis software by FlowJo, LLC (Becton, 20 Dickinson and Company, USA). Data are presented as mean ± SD (n= 3, biological triplicates).

Cellular association and transfection efficacy evaluated by flow cytometry

HeLa cells (18,000 cells per well) were seeded in 24-well plates 24 h before transfection to evaluate the cell association and expression kinetics. Polyplexes were either formed at N/P 18 for U-shape or N/P 24 for Bundle LAF-XPs with a mixture of CleanCap® EGFP mRNA (5moU) (80 wt%) and EZ Cap Cy5 Firefly Luciferase mRNA (5moUTP) (20 wt%) at a total mRNA concentration of 12.5 g mL^{-1}. The cells were transfected in biological duplicates with a dose of 100 ng mRNA per well and incubated for 1 h, 2 h, 4 h, 6 h, 24 h and 48 h. Afterwards, the cells were prepared for flow cytometry-based analysis. The medium was removed, and the cells were gently washed with cold PBS (4 °C) and trypsinized for 5 min at 37 °C. Next, the cell suspension was resuspended with 500 µL of FACS buffer (10% FBS in PBS without DAPI) and transferred to a FACS tube. Cells were analyzed by flow cytometry using a CytoFLEX S Flow Cytometer (Beckman Coulter, Brea, CA, USA) over 60 seconds at a flow rate of 170 µL min^{-1}. Flow cytometry data were analyzed using FlowJo™ v10.8 flow cytometric analysis software by FlowJo, LLC (Becton, 20 Dickinson and Company, USA). Data are presented as mean ± SD (n= 2, biological duplicates).

Endosomal disruption assessed by a Galectin 8 assay

HeLa Gal8 mRuby3 cells (15,000 per well) were seeded in an ibidi µ-slide 8-well 24 h prior to transfection. Before treatment, the cell culture medium was replaced with 296.4 µL fresh medium and the assay was performed at a dose of 45 ng mRNA per well. Hence, 3.6 µL of polyplex solution formed at an mRNA concentration of 12.5 ng μL^{-1}, containing 20% (w/w) Cy5 labelled, and 80% (w/w) unlabeled firefly luciferase mRNA were transferred to the corresponding well. After transfection, the cells were incubated for 4 h. The medium was removed, cells were washed twice with PBS, subsequently fixed with 4% (w/v) paraformaldehyde in PBS and stored in the dark at RT for 1 h. Next, cells were washed twice with PBS and nuclei were stained with DAPI (1 µg mL^{-1}) at RT for 30 min in the dark. The

staining solution was replaced with fresh PBS. Cells were stored under light protection at 4 °C until imaging. Imaging was performed with a Leica-TCS-SP8 CLSM equipped with an HC PL APO 63× 1.4 objective and images were processed with the LAS X software from Leica. Puncta counting from 4 images per sample was performed with ImageJ 1.54g (Java 1.8.0_345) [13] by smoothing and subsequent counting of maxima in the DAPI channel to count nuclei (20 to 44 cells per image, prominence >30.0) and mRuby3 channel to count Gal8 recruitment sites (prominence >10). Results are presented as mean Gal8 puncta per cell ± SD. To assess the relationship between endosomal membrane destabilization and functional delivery outcomes, the Spearman correlation coefficient was calculated between the number of Gal8 puncta per cell and either the percentage of GFP-positive cells or relative metabolic activity.

Molecular dynamics simulations

2D mol files of the carriers were generated in-house and processed using a custom RDKit-based Python script for hydrogen addition and 3D embedding. Mol2 files were generated with Open Babel and parametrized using AmberTools22 with GAFF2 atom types and Gasteiger charges (Antechamber), followed by Parmchk2. Topologies and PDB files were prepared with tleap (including water and counterions) and converted to GROMACS input using ParmEd and an in-house script. Carriers and octanol were described with the amber-derived GAFF2 force field, and TIP3P water with corresponding ion parameters was used. As all molecules were treated consistently across conditions, the reduced absolute accuracy of the charge model is expected to have limited impact on relative comparisons between systems.

All simulations employed Particle Mesh Ewald (PME) electrostatics with a real-space cutoff of 1.2 nm. Lennard–Jones interactions were treated using a force-switch modifier from 1.0 to 1.2 nm. All bonds involving hydrogen atoms were constrained using LINCS, and integration was performed using the leap-frog algorithm. Periodic boundary conditions were applied in all dimensions. Temperature was maintained at 289.15 K and pressure at 1 atm, unless stated otherwise pressure coupling was isotropic.

Water–octanol interface simulations

Carriers were placed at a pre-equilibrated water–octanol interface using GROMACS 2023.5 and energy-minimized in a stepwise manner for 50,000 steps. This was followed by a 200 ps NVT equilibration using a Nosé–Hoover thermostat, during which the carrier was pulled into the octanol phase using Gromacs. Subsequently, a 1.5 ns NPT equilibration was performed using a Berendsen barostat and Nosé–Hoover thermostat. Production runs (n=3) were performed in triplicate for 10 ns using the Parrinello–Rahman barostat and v-rescale thermostat.

DOPC/cholesterol membrane simulations

A DOPC/Cholesterol bilayer comprising 100 lipids (70 DOPC/ 30 Cholesterol) per leaflet was generated with the CHARMM-GUI Membrane Builder and parametrized using the Lipid21 force field. Prior to carrier placement, the membrane was equilibrated for 100 ns under NPT conditions. The full systems were constructed in an 8.2 × 8.2 × 14 nm simulation box, then solvated, charge-neutralized, and brought to 0.15 M NaCl. Following energy minimization (50 000 steps), the protocol included a 125 ps NVT equilibration using a v-rescaling thermostat, followed by an additional 125 ps NVT stage in which the carriers were steered into the bilayer midplane under Nose–Hoover temperature coupling using Gromacs (insertion depth into the box was 6 nm). Membrane adaptation was then achieved through sequential NPT equilibration steps: 1.25 ns using a Berendsen barostat with separate V-rescale thermostat groups (for solvent, carrier and membrane), and 10 ns using a C-rescale barostat. Finally, 100 ns NPT production (n=3) simulations were carried out employing PME for long-range electrostatics and a dispersion correction. Semiisotropic pressure coupling was applied for all NPT simulations in this experiment.

Self-assembly simulations

For self-assembly simulations in 0.9% (w/v) NaCl, 150 copies of the parametrized carriers were randomly placed in a cubic simulation box (20 nm edge length) under periodic boundary conditions. Systems were neutralized and solvated with TIP3P water following GROMACS conventions. After stepwise energy minimization (50,000 steps), systems were equilibrated for 200 ps in the NVT ensemble using a Nosé–Hoover thermostat, followed by 1.5 ns in the NPT ensemble using a Nosé–Hoover thermostat and Berendsen barostat. Production simulations were performed as single trajectories (n=1) (one replica) per carrier state for 140 ns using a 1.5 fs time step, employing the Parrinello–Rahman barostat and v-rescale thermostat. Trajectory frames were saved every 7.5 ps.

To reduce storage requirements, a reduced trajectory (XTC) without water molecules was saved for the self-assembly simulations. For analyses requiring water, RDFs were computed from the corresponding water-containing coordinates.

Analysis

Trajectories were analyzed using GROMACS tools and MDAnalysis together with in-house Python scripts. System compositions are provided in Tables S3, S4 and S5. All carriers were simulated under identical conditions within each simulation class, and results are interpreted primarily as relative trends between systems.

Synchrotron small-angle X-ray scattering

Synchrotron small-angle X-ray scattering (SAXS) was carried out at the P12 EMBL BioSAXS and P62 SAXSMAT beamlines, PETRA III, DESY (Hamburg, Germany). The beamline instrumentation has been described previously [6,7]. All the measurements were performed in borosilicate glass capillaries purchased from Hilgenberg, except for the self-assembly measurements of carriers 1718 and 1960, which were performed at the automated flow-through sample environment of P12. The scattering data background was subtracted by measuring empty capillaries for the bulk phase samples and the corresponding buffer for samples in solution. The data for self-assembly were fitted using the unilamellar vesicle model [8]. The crystallographic space groups of the liquid crystalline phases were determined from relative peak positions.

Fitting of the solution SAXS data

SAXS data of the LAF-Stp XPs dispersed in aqueous buffer were fitted using a large unilamellar vesicle (LUV) model implemented into the jscatter free software [9]. The broad peak observed for all samples is typical of the lipid bilayer form factor when the scattering contrast of the tail region relative to the buffer has the opposite sign to that of the head groups. This scenario is common in X-ray scattering from lipid bilayers. When the contrast of the heads and tails relative to the buffer has the same sign, as well as for various homogeneous or core–shell particles, the correlation peaks oscillate much more frequently and fail to fit the data.
Importantly, the fitted region is dominated by the bilayer form factor, while the overall particle form factor contributes only weakly in the measured Q-range. Therefore, we cannot determine the overall particle shape with confidence, and a spherical shape is the minimal assumption based on the energy minimization argument. The particle sizes obtained by DLS fall within the typical LUV range of 50–200 nm.
For each LAF-Stp XP, the samples at different pH values were fitted simultaneously. The headgroup thickness *a* was constrained to be the same across all pH values, whereas the tail thickness *b* and the headgroup SLD (*SLDa*) were allowed to vary. The SLD of the tails was fixed at 8.68×10^{-4} nm^{-1}. For LAF-Stp XP 1718, a second headgroup layer had to be included. To improve the fit of the second form-factor maximum, bilayer thickness fluctuations were taken into account [10]. For all samples, the thickness fluctuation *d*s was fixed to 0.1 nm, and some fits benefited from adding a Debye–Waller factor to the model [11].This effectively reduces the intensity of higher-order peaks while increasing the background at large *Q* values, and describes the local loss of correlations (thermal displacement fluctuations) in the bilayer. This contribution improved the fits of samples 1621 and 1813.

Table S7 summarizes the fit results and the χ^2 values for all measured samples. Figure S19 shows two examples of the residual distributions. The minor deviations between the fit and the data may be due to a more complex scattering length density distribution than the step profile used in the model.

Statistical analysis

Results are presented as arithmetic mean of triplicates, if not stated otherwise ± standard deviation (SD), displayed with error bars. In terms of the bafilomycin A1 assay, statistical analysis of the decrease in transfection efficacy and cellular uptake (mean ± SD) were performed using unpaired Student's two-tailed t-test with Welch's correction. For stability studies and batch-to-batch comparison, results were analyzed by Brown-Forsythe and Welch ANOVA test. Correlations between Gal8 recruitment and transfection efficacy or relative metabolic activity were performed via determining the Spearman correlation coefficient. All calculations were conducted with GraphPad Prism 10 and significance levels indicated with symbols: ns (statistically not significant) $p > 0.05$; *$p \leq 0.05$; **$p \leq 0.01$; ***$p \leq 0.001$; ****$p \leq 0.0001$. Affinity Designer 218.2 (version 2.5.7, Serif Ltd., West Bridgford, UK), VMD 2.0, and GraphPad Prism were used for visualization. Some graphics contain elements created in BioRender. Due to the small sample size (n=3), assumptions of normality and variance homogeneity could not be formally tested. The chosen statistical approaches (Welch's correction and Brown-Forsythe ANOVA) are therefore considered robust methods suitable for these small-sample comparisons.

2. **Supporting data**

Table S1. Chemical characteristics of U-shape and Bundle LAF-Stp xenopeptides

**ID**	**code**	**sequence**	**protonatable amines**	**stock solution**
			U-shapes	**EtOH/H_2O**
**1611**	12Oc-K-U1-1:2	H_2N-K(12Oc)-Stp-K(12Oc)	6	1:1
**1827**	12Oc-O-U1-1:2	H_2N-O(12Oc)-Stp-O(12Oc)	6	1:1
**1718**	12Oc-K-U1-1:4	H_2N-[K(12Oc)]$_2$-Stp-[K(12Oc)]$_2$	8	7:3
**1960**	12Oc-O-U1-1:4	H_2N-[O(12Oc)]$_2$-Stp-[O(12Oc)]$_2$	8	7:3
			Bundles	**EtOH/HCl**
**1621**	8Oc-K-B2-1:4	K[K(8Oc)$_2$]$_2$-Stp-OH	7	9:1
**1813**	8Oc-O-B2-1:4	O[O(8Oc)$_2$]$_2$-Stp-OH	7	9:1
**1752**	12Bu-K-B2-1:4	K[K(12Bu)$_2$]$_2$-Stp-OH	7	9:1
**1814**	12Bu-O-B2-1:4	O[O(12Bu)$_2$]$_2$-Stp-OH	7	9:1

Table S2. Physicochemical characterization of mRNA polyplexes containing distinct U-shape and Bundle LAF-Stp xenopeptides. Determination of size distribution (Z-average), polydispersity (PDI) and zeta potential was carried out in three technical replicates. Data are presented as mean ± SD.

			Z-average [nm]		PDI		zeta potential [mV]	
**ID**	**code**	**N/P**	**mean**	**SD**	**mean**	**SD**	**mean**	**SD**
**U-shapes**								
**1611**	12Oc-K-U1-1:2	12	72.7	2.4	0.286	0.043	21.8	3.8
		18	61.9	1.0	0.21	0.032	23.8	2.4
		24	68.6	2.6	0.256	0.041	28.4	2.6
**1827**	12Oc-O-U1-1:2	12	9253.3	1228.92	0.385	0.054	21.1	1.8
		18	1637.0	59.6	0.467	0.081	22.8	0.3
		24	944.0	309.7	0.632	0.467	24.2	1.7
**1718**	12Oc-K-U1-1:4	12	158.6	2.1	0.241	0.008	36.1	2.7
		18	167.8	1.8	0.253	0.002	37.8	1.6
		24	151.2	3.2	0.209	0.006	39	2.3
**1960**	12Oc-O-U1-1:4	12	127.1	2.4	0.124	0.014	13	1.1
		18	112.1	0.9	0.136	0.012	17.5	0.9
		24	101.7	2.6	0.201	0.024	18	0.1
**Bundles**								
**1621**	8Oc-K-B2-1:4	12	105.5	0.4	0.124	0.009	11.1	0.9
		18	96.6	1.4	0.144	0.014	13.9	0.9
		24	103.3	1.9	0.176	0.023	19.7	0.8
**1813**	8Oc-O-B2-1:4	12	161	2.0	0.124	0.02	8.1	0.9
		18	113.3	1.0	0.118	0.017	12.2	1.1
		24	99.9	1.4	0.133	0.021	19.7	0.5
**1752**	12Bu-K-B2-1:4	12	235.2	1.7	0.212	0.013	-12.2	1.0
		18	106.3	1.6	0.126	0.009	-3.5	0.3
		24	112	1.8	0.206	0.026	5.4	0.1
**1814**	12Bu-O-B2-1:4	12	3138.3	163.2	0.39	0.17	14.3	1.5
		18	99.5	1	0.148	0.021	20.1	2.4
		24	93.1	3.6	0.204	0.029	21.1	1.4

Table S3. Evaluation of polyplex formation reproducibility and storage stability. Representative U-shape and Bundle XPs were assembled into two independent mRNA polyplexes containing an mRNA concentration of 12.5 ng µL^{-1}. Size distribution (Z-average) and polydispersity (PDI) of each formulation were evaluated at day 1 or after 7 days of storage at 4 °C by DLS. Data are presented as mean ± SD (n=3, technical replicates).

			day 0				day 7			
ID	code	batch	Z-average [nm]		PDI		Z-average [nm]		PDI	
			mean	SD	mean	SD	mean	SD	mean	SD
1611	12Oc-K-U1-1:2	1	90	3.6	0.205	0.001	87.7	1.6	0.207	0.007
		2	86.6	0.8	0.197	0.005	97	4.9	0.277	0.047
1827	12Oc-O-U1-1:2	1	107.7	2.7	0.158	0.019	106.7	2.8	0.159	0.014
		2	111.5	1.9	0.163	0.013	113.8	2.5	0.186	0.023
1621	8Oc-K-B2-1:4	1	105.8	1.1	0.208	0.019	103.3	2	0.222	0.003
		2	107.8	1.9	0.155	0.013	106.2	2.5	0.222	0.024
1813	8Oc-O-B2-1:4	1	95.7	2.6	0.193	0.023	107.3	3.8	0.241	0.013
		2	94.5	2.5	0.193	0.036	96.2	2.7	0.207	0.006

Table S4. Statistical analysis of luciferase expression levels between fresh (day 1) and two independently formed 7-day-old polyplex formulations. Analysis was performed in GraphPad Prism 10 via one-way ANOVA with Brown-Forsythe/Welch correction. Significance levels: ns $p > 0.05$; * $p \leq 0.05$; ** $p \leq 0.01$; *** $p \leq 0.001$; **** $p \leq 0.0001$.

		fresh batch vs. batch 1 day 7		fresh batch vs. batch 2 day 7		batch 1 day 7 vs. batch 2 day 7	
ID	code	adjusted p-value	significance level	adjusted p-value	significance level	adjusted p-value	significance level
1611	12Oc-K-U1-1:2	0.3303	ns	0.3583	ns	0.8034	ns
1827	12Oc-O-U1-1:2	0.1457	ns	0.9955	ns	0.1757	ns
1621	8Oc-K-B2-1:4	<0.0001	****	0.0022	**	0.0712	ns
1813	8Oc-O-B2-1:4	0.1002	ns	0.0774	ns	0.2108	ns

Table S5. LogD evaluation of distinct C-terminal tyrosine-modified LAF-Stp XPs at physiological, early and late endosomal pH determined via UV/Vis photometric determination.

ID	code	logD [pH 7.4]	logD [pH 6.5]	logD [pH 5.5]
**1895**	1813-Y-(8Oc-O-B2-1:4)	0.78	-0.29	-0.75
**1896**	1814-Y-(12Bu-O-B2-1:4)	0,84	-0,17	-0,7
**1897**	1827-Y-(12Oc-O-U1-1:2)	0.56	-0.45	-0.82

Y, tyrosine; Stp, succinoyl tetraethylene pentamine

Table S6. Determination of the size distribution and polydispersity of large unilamellar vesicles formed by LAF-Stp XPs at distinct pH's via DLS. Determination of size distribution (Z-average) and polydispersity (PDI) was carried out in three technical replicates. Data are presented as mean ± SD.

			**Z-average [nm]**		**PDI**	
**ID**	**Code**	**pH**	**mean**	**SD**	**mean**	**SD**
**U-shapes**						
**1611**	12Oc-K-U1-1:2	7.4	78.8	4.9	0.541	0.021
**1611**	12Oc-K-U1-1:2	5.5	165.5	7.9	0.578	0.029
**1827**	12Oc-O-U1-1:2	7.4	92.5	3.1	0.529	0.014
**1827**	12Oc-O-U1-1:2	5.5	144.7	63	0.449	0.129
**Bundles**						
**1621**	8OC-K-B2-1:4	7.4	136.6	3.7	0.261	0.016
**1621**	8OC-K-B2-1:4	5.5	142.5	3.1	0.682	0.005
**1813**	8OC-O-B2-1:4	7.4	137.9	3.6	0.401	0.029
**1813**	8OC-O-B2-1:4	5.5	70.7	0.4	0.397	0.006
**1752**	12Bu-K-B2-1:4	7.4	176.7	4.4	0.488	0.025
**1752**	12Bu-K-B2-1:4	5.5	42.1	1.2	0.551	0.127
**1814**	12Bu-O-B2-1:4	7.4	284.7	10.8	0.318	0.008
**1814**	12Bu-O-B2-1:4	5.5	31.7	2	0.506	0.035

Table S7A Box composition for water-octanol interface simulations.

Simulation	Carrier	Ions	Octanol	Water
1621 zw	1	0	454	3473
1621 all2	1	3	453	3540
1621 all4	1	5	453	3508
1611 zw	1	0	454	3538
1611 all1	1	2	454	3554
1611 all2	1	3	453	3538

Table S7B Box composition for Self-assembly simulations

Simulation	Carrier	Na	Cl	Water
1621 zw	150	742	742	240583
1621 all2	150	517	967	240525
1621 all4	150	367	1117	240739
1611 zw	150	742	742	246738
1611 all1	150	592	892	246661
1611 all2	150	517	967	245882

Table S8. Summary of the fit results for SAXS of the LAF-Stp XPs in aqueous buffer. The model of unilamellar vesicles, including thickness fluctuations, was used. As we are not sensitive to LUV size in the fitted Q range, in order to avoid overfitting, it was fixed to *R* = 35 nm with polydispersity *dR* = 8 nm for all the samples.

	ID	pH	χ^2	a, nm	a_2, nm	b, nm	SLDa, 10^{-4} nm^{-1}
U-shapes	1611	5.5	3.23	2.38±0.02	-	1.57±0.03	10.16±0.02
		6.5				1.61±0.03	10.13±0.02
		7.4				1.72±0.03	9.91±0.01
	1827	5.5	1.25	2.70±0.04	-	1.24±0.06	9.76±0.02
		6.5				1.16±0.06	9.73±0.02
	1718	5.5	5.42	1.42±0.01	2.32±0.01<br>$SLDa_2$= $(10.08±0.01)·10^{-4}$	1.27±0.01	9.62±0.01
		6.5				1.34±0.01	9.61±0.01
		7.4				1.92±0.01	9.59±0.01
	1960	5.5	2.98	2.13±0.01	-	1.35±0.01	9.62±0.01
		6.5				1.55±0.01	9.61±0.01
		7.4				1.61±0.01	9.59±0.01
Bundles	1621	5.5	1.52	2.15±0.05	-	1.05±0.08	9.79±0.03
		6.5				0.98±0.08	9.73±0.03
		7.4				1.06±0.08	9.73±0.03
	1813	5.5	1.41	2.18±0.13	-	0.76±0.24	9.69±0.10
		6.5				0.76±0.24	9.67±0.09
		7.4				0.78±0.24	9.65±0.08
	1752	5.5	1.51	2.45±0.01	-	0.79±0.02	9.61±0.01
		6.5				0.63±0.02	9.57±0.01
		7.4				0.77±0.02	9.58±0.01
	1814	5.5	1.65	2.55±0.01	-	0.63±0.01	9.57±0.01
		6.5				0.51±0.01	9.55±0.01
		7.4				0.61±0.01	9.55±0.01

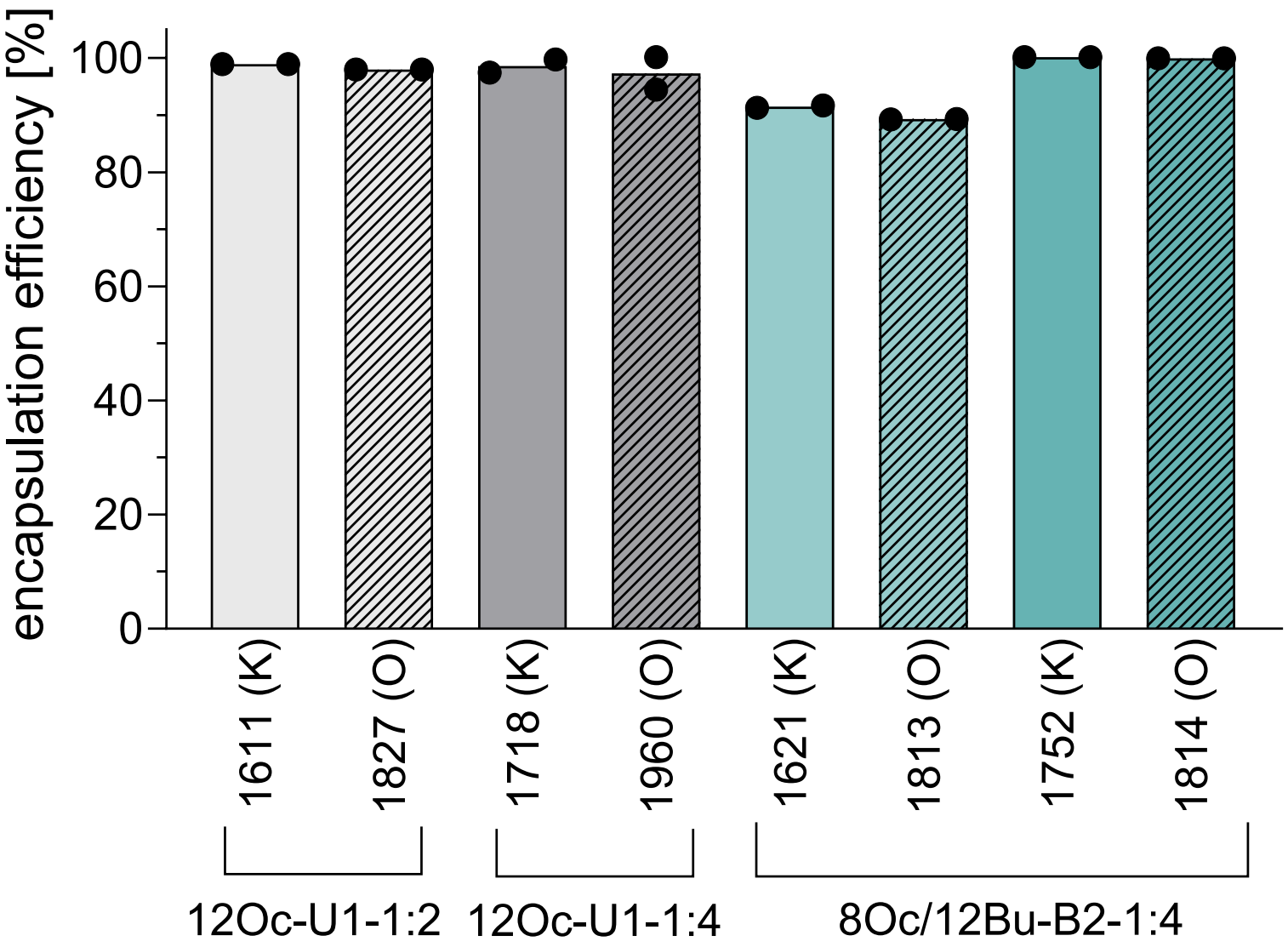

Figure S1. Encapsulation efficiency of mRNA LAF-Stp polyplexes determined with Quant-iT™ RiboGreen RNA Assay-Kit. Polyplexes were formed at N/P 18 (U1-1:2 and U1-1:4) and N/P 24 (B2-1:4), containing 12.5 µg mL^{-1} of mRNA. Fluorescence of free mRNA interacting with RiboGreen reagent in polyplexes in comparison to polyplexes treated with 2% Triton and 250 I.U. mL^{-1} heparin was measured. All samples and control measurements were performed as technical duplicates. Data are presented as individual data points (n=2).

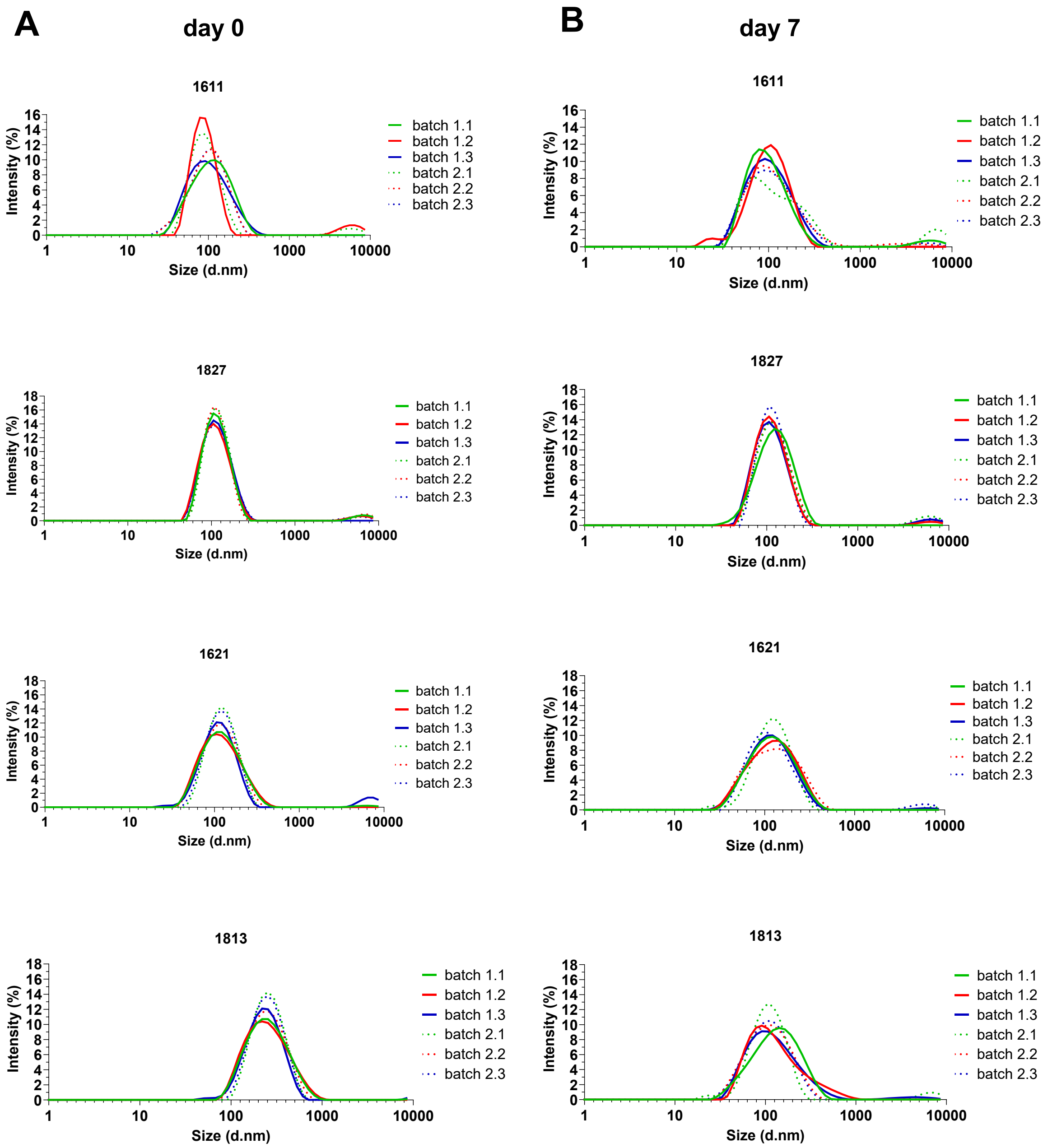

Figure S2. Size distribution of mRNA polyplexes containing distinct U-shape (12Oc-K-U1-1:2, *1611* and 12Oc-O-U1-1:2, *1827*) and Bundle XPs (8Oc-K-B2-1:4, *1621* and 8Oc-O-B2-1:4, *1813*). Comparison of two independently formed LAF-Stp XP polyplex batches at **A** day 0 and **B** 7 days after formulation and storage at 4 °C.

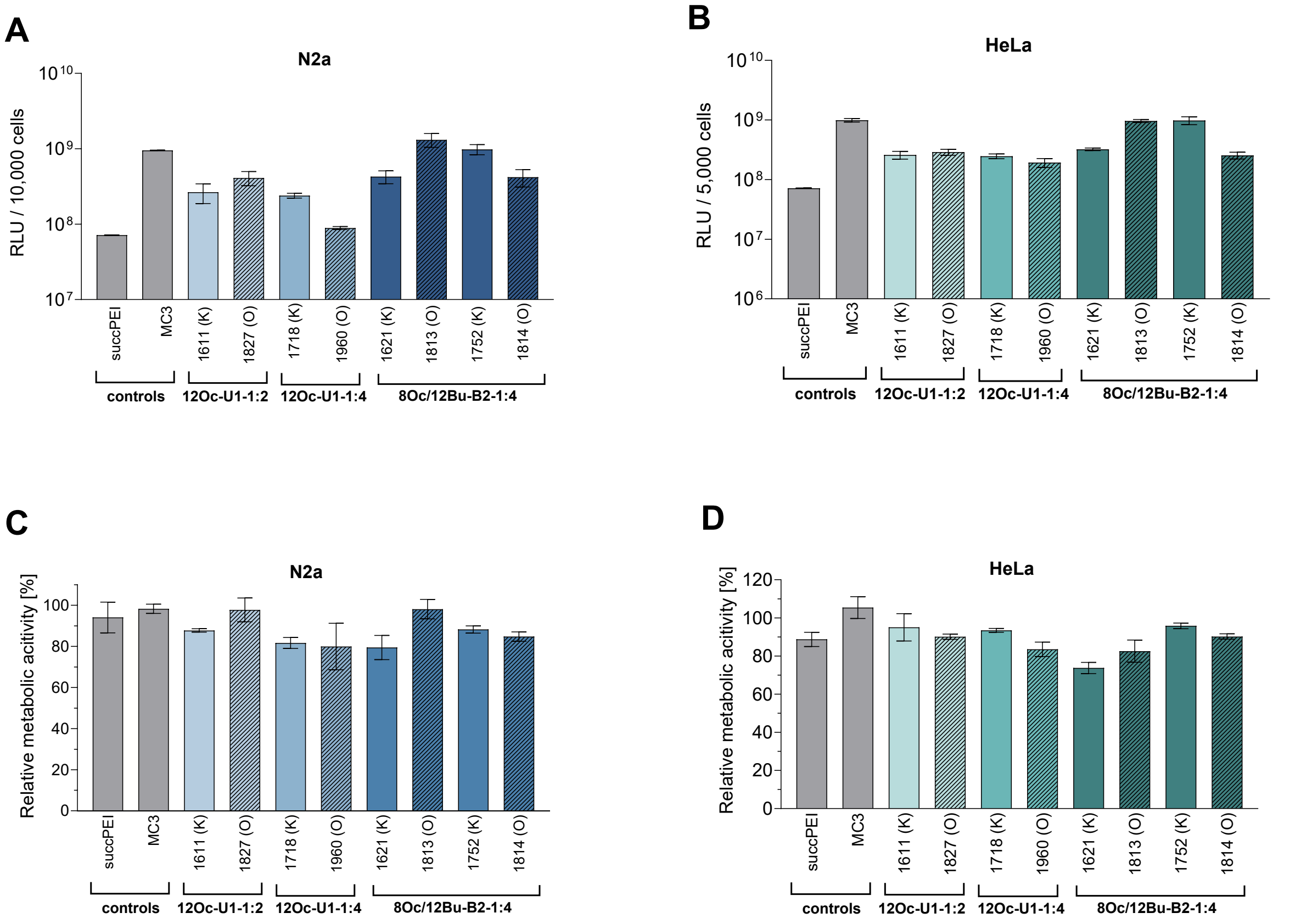

Figure S3. *In vitro* evaluation of mRNA polyplexes assembled with LAF-Stp XPs at N/P 18 (U1-1:2 and U1-1:4) or N/P 24 (B2-1:4). Transfections were conducted at a dose of 15 ng mRNA per well in biological triplicates. Luciferase expression in **A** N2A (10,000 cells per well) and **B** HeLa (5,000 cells per well) cells 24 h after transfection. Relative metabolic activity of **C** N2A (10,000 cells per well) and **D** HeLa (5,000 cells per well) evaluated via MTT assay 24 h after transfection. Cells treated with HBG were set to 100%. For both experiments data are presented as mean (n= 3) ± SD.

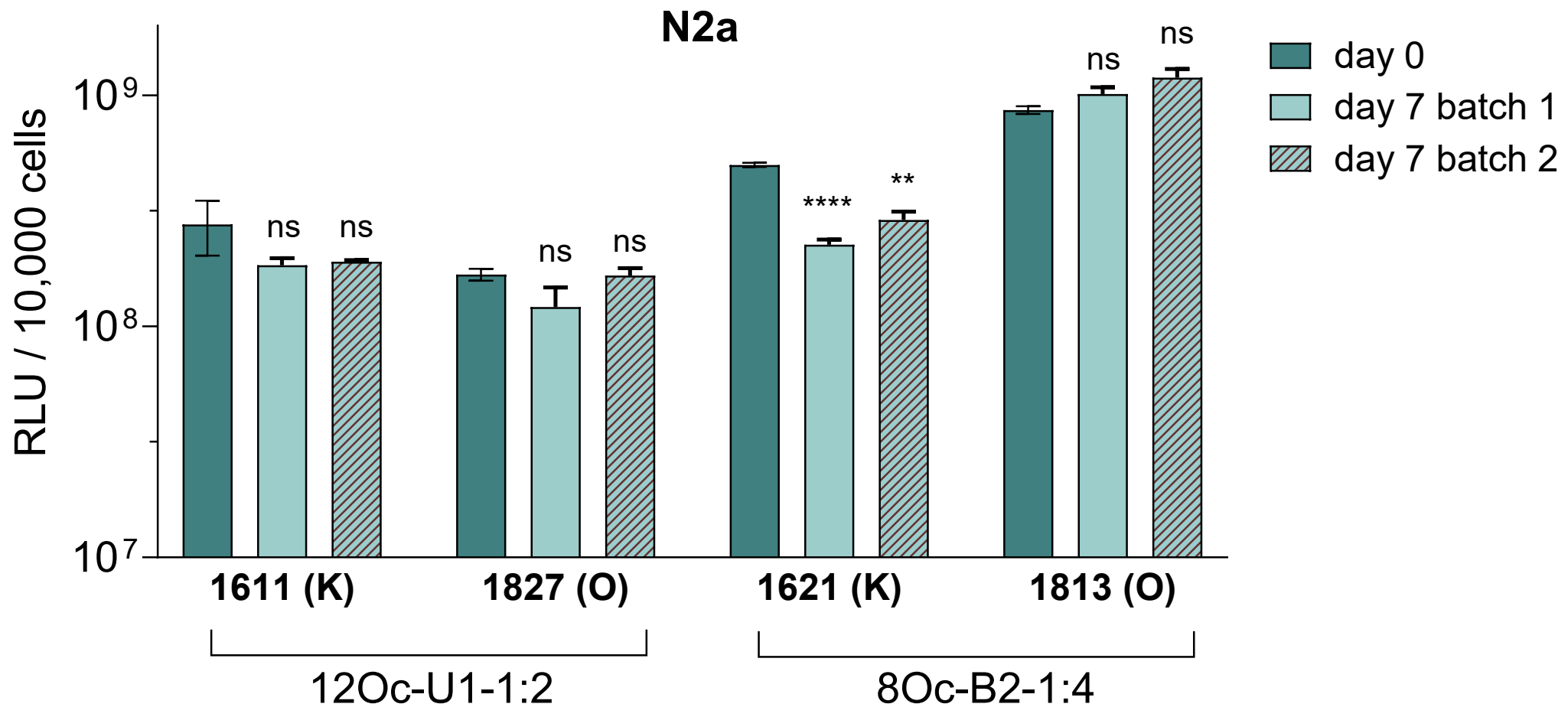

Figure S4. Transfection efficacy of freshly prepared mRNA polyplexes containing distinct LAF-Stp XPs compared to polyplexes stored at 4 °C for 7 days. Polyplexes were formed at an mRNA concentration of 12.5 µg mL^{-1}. Transfection was carried out in N2a cells, followed by read-out via luciferase expression assay 24 h after transfection. Data are presented as mean ± SD (n=3, biological triplicates). Statistical analysis was performed using GraphPad Prism 10. Differences between fresh (day 0) and two independently assembled 7-day-old polyplex batches were analyzed by Brown-Forsythe and Welch ANOVA test. Significance levels in the figure relate to the analysis between fresh and 7-day-old mRNA polyplexes, respectively. Significance levels: ns p > 0.05; * p ≤ 0.05; ** p ≤ 0.01; *** p ≤ 0.001; **** p ≤ 0.0001.

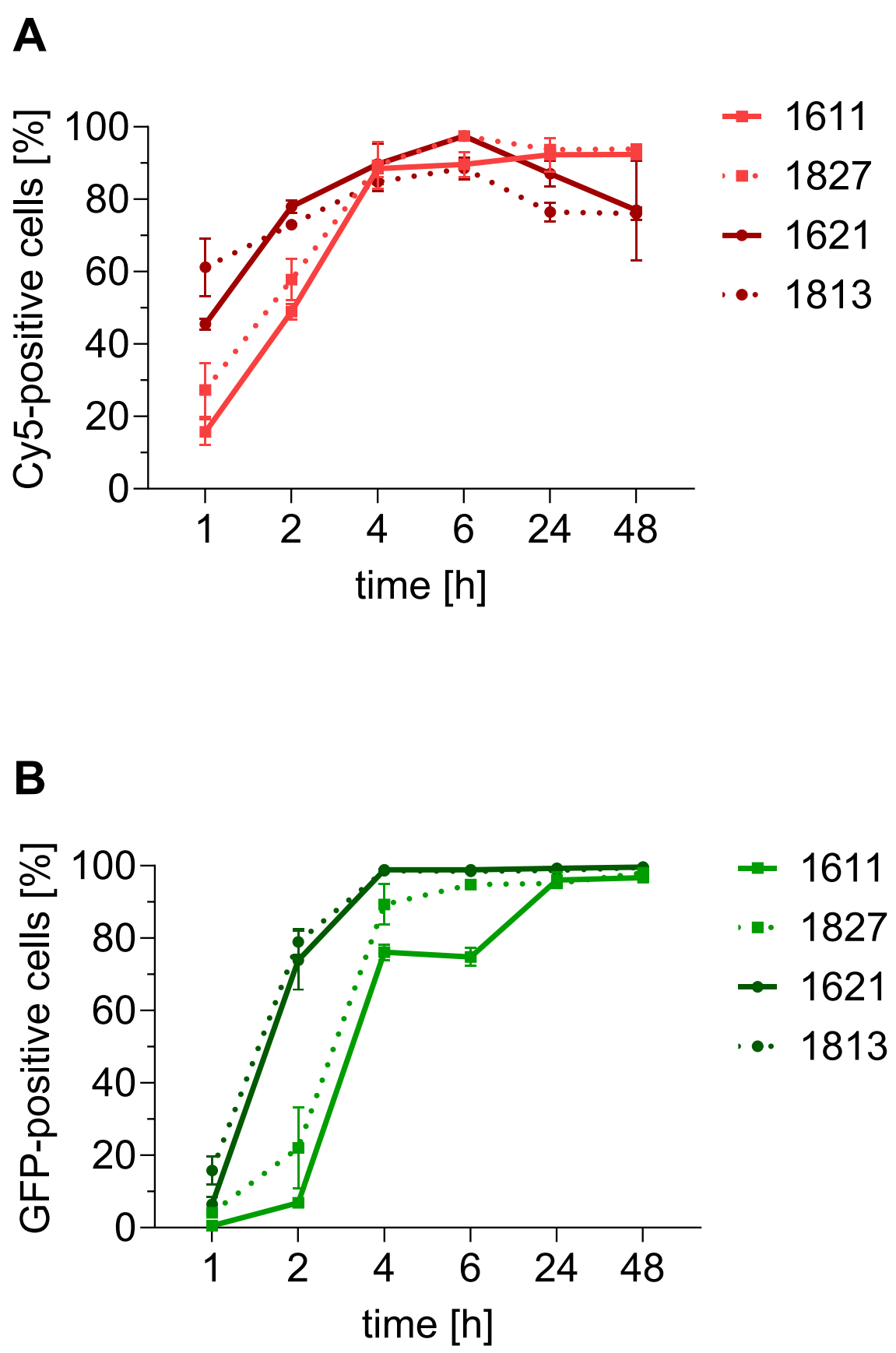

Figure S5. Kinetic study of cellular association and expression. HeLa cells (18,000 cells per well) were transfected with polyplexes, formed with respective U-shape and Bundle XPs at N/P 18 (U1-1:2) or N/P 24 (B2-1:4) to reach a mRNA concentration of 12.5 ng μL^{-1} containing GFP (80 wt%) and Cy5-mRNA (20 wt%). Transfections were conducted at a dose of 100 ng mRNA per well in biological duplicates. Cells were analyzed by flow cytometry at the indicated time points. **A** Cellular association and uptake of mRNA polyplexes represented by Cy5-positive cells. **B** Transfection efficacy demonstrated by GFP-positive cells. Data are presented as mean ± SD (n=2, biological replicates).

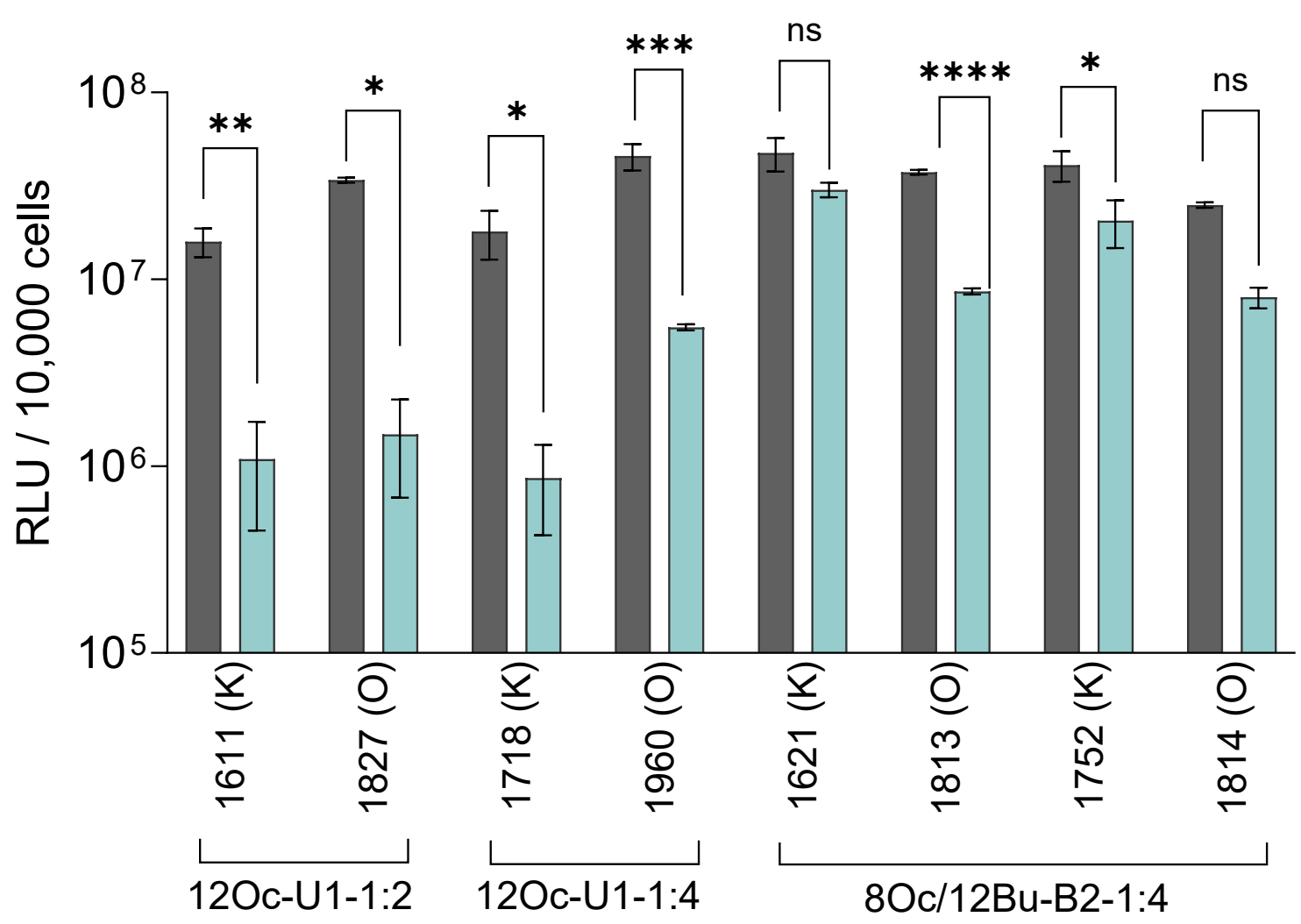

Figure S6. Impact of bafilomycin A1 on luciferase expression in N2a cells (10,000 cells per well). Transfection efficacy was determined 4 h post transfection. Data are presented as mean ± SD. Statistical analysis was conducted via unpaired Student's two-tailed t-test with Welch's correction. Significance levels: ns p > 0.05; * p ≤ 0.05; ** p ≤ 0.01; *** p ≤ 0.001; **** p ≤ 0.0001.

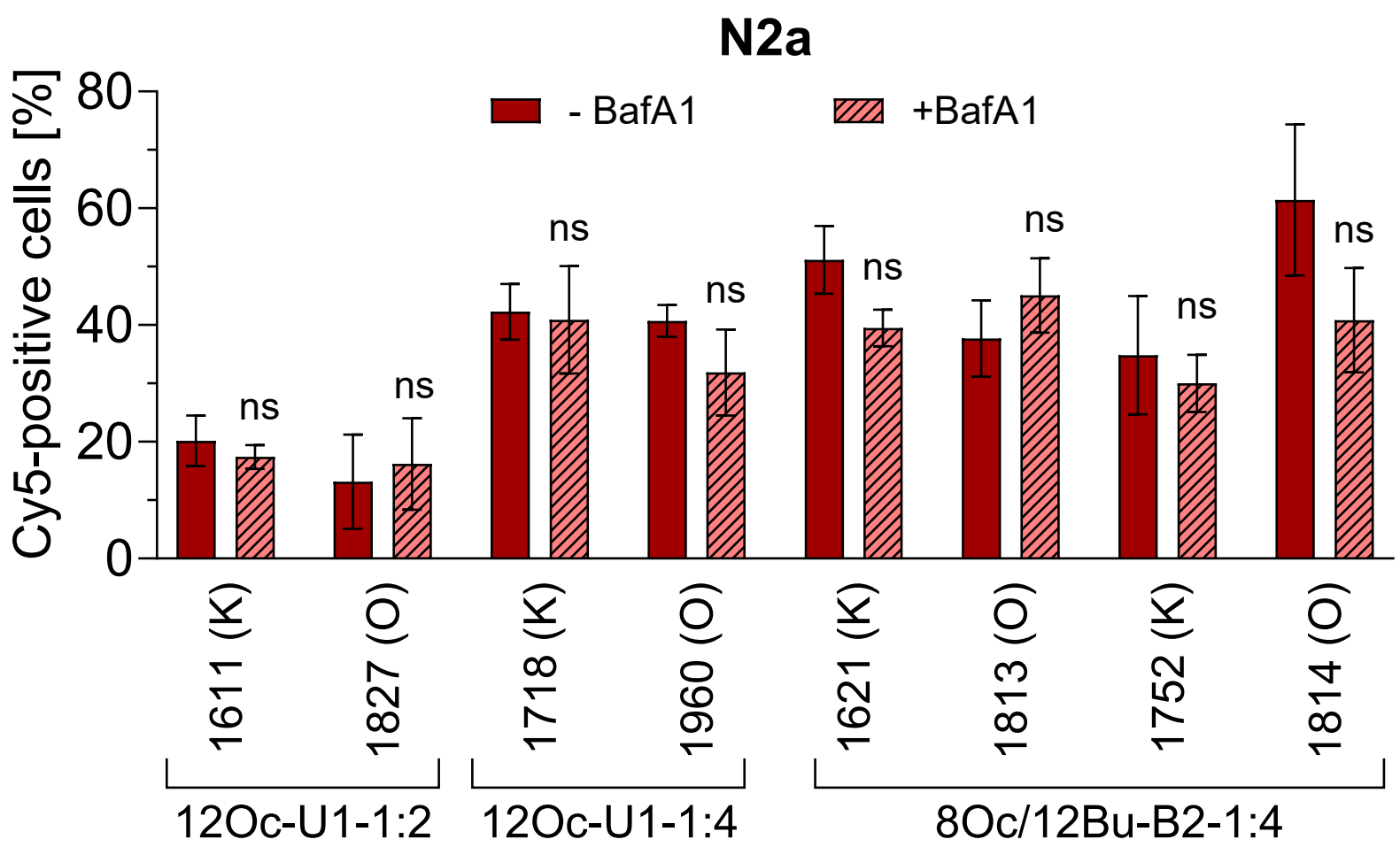

Figure S7. Cellular uptake of LAF-Stp XP polyplexes evaluated in the presence or absence of Bafilomycin A1. N2a cells (10,000 cells per well) were transfected with polyplexes containing U-shape (N/P 18) or Bundle XPs (N/P 24) and 12.5 µg mL $^{-1}$ mRNA (80 wt % Luc-mRNA and 20 wt% Cy5-mRNA). Transfections were performed at an mRNA dose of 15 ng per well. Cells were analyzed via flow cytometry after 2 h. Cellular uptake of mRNA polyplexes represented as Cy5-positive cells. Data are provided as mean ± SD (n=3, biological triplicates). Statistical analysis was conducted via unpaired Student's two-tailed t-test with Welch's correction in GraphPad Prism 10. Significance levels: ns p > 0.05; * p ≤ 0.05; ** p ≤ 0.01; *** p ≤ 0.001; **** p ≤ 0.0001.

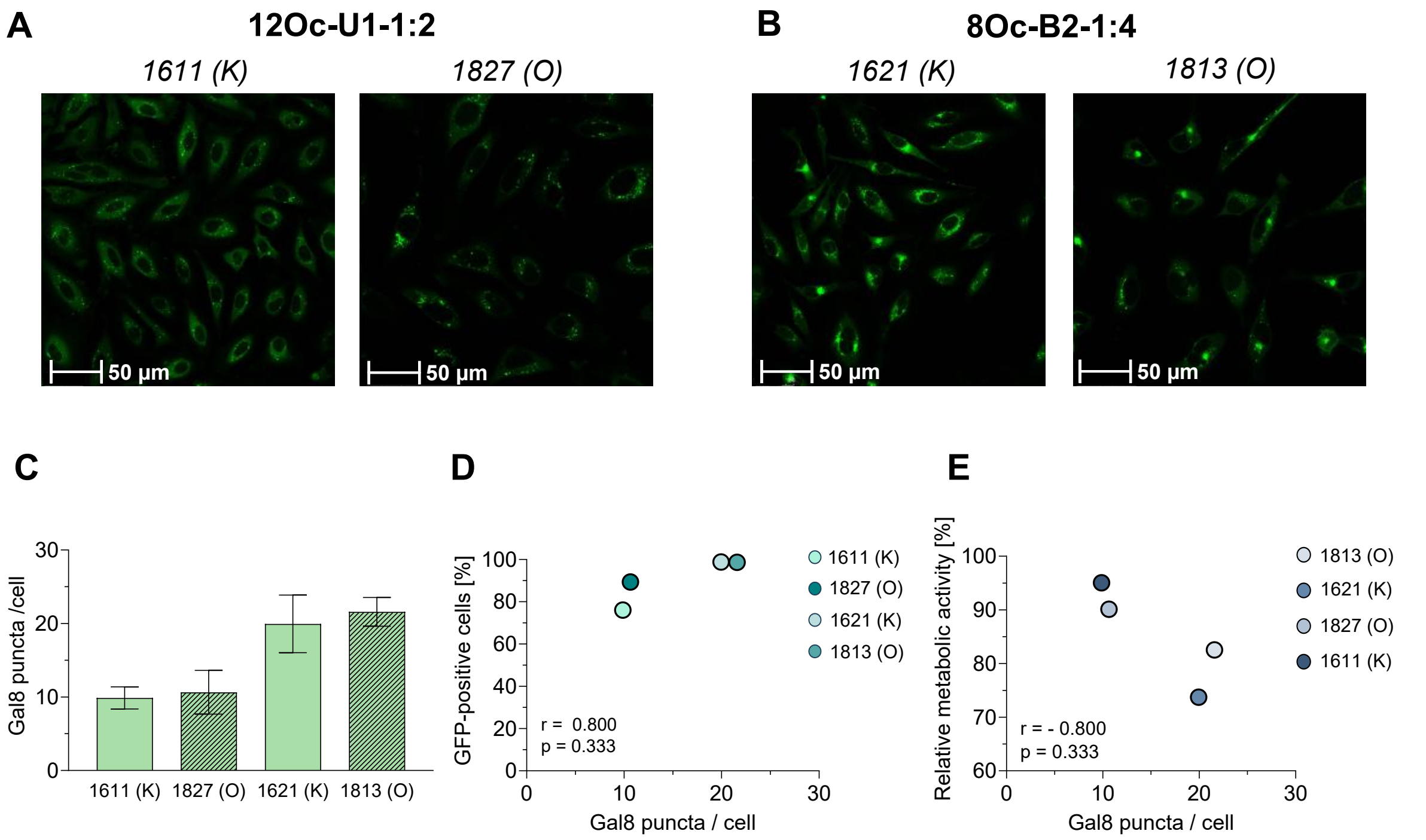

Figure S8. Endosomal disruption of mRNA polyplexes evaluated in HeLa-Gal8-mRuby3 cells at an mRNA dose of 45 ng per well 4 h post transfection via confocal laser scan microscopy. Representative CLSM images of **A** U-shape or **B** Bundle LAF-Stp XP polyplexes. **C** Quantification of Gal8 recruitment via puncta counting from 4 images per sample in ImageJ 1.54g (Java 1.8.0_345) [13] by smoothing and subsequent counting of maxima in the DAPI channel to count nuclei (20 to 44 cells per image, prominence >30.0) and mRuby3 channel to count Gal8 recruitment sites (prominence >10). Results are indicated as mean Gal8 puncta per cell ± SD. Spearman correlation between Gal8 puncta and **D** transfection efficacy presented as GFP-positive cells at 100 ng mRNA per well in HeLa cells (18,000 cells per well) 4 h post transfection and **E** relative metabolic activity evaluated in HeLa cells (5,000 cells per well) 24 h after transfection.

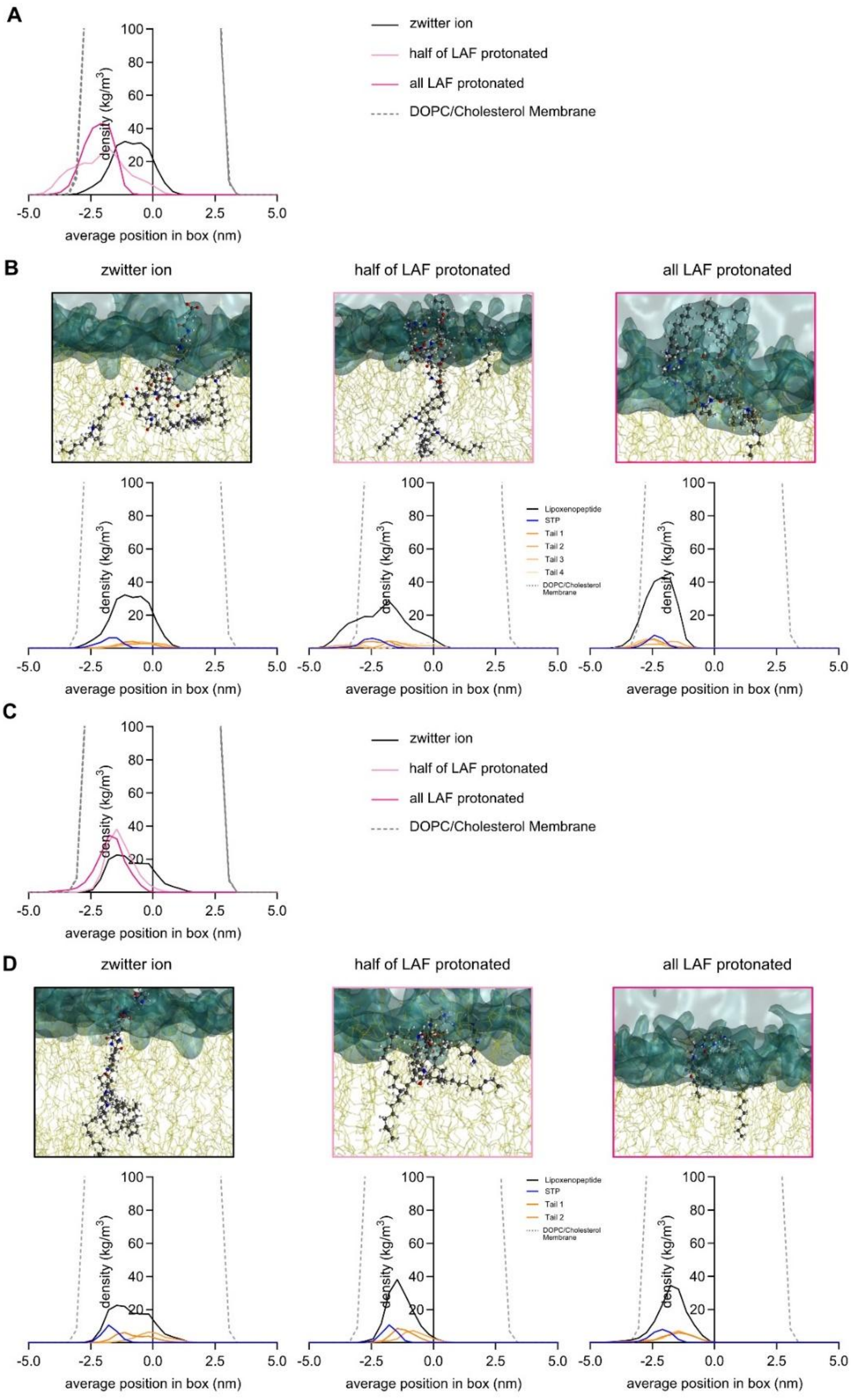

Figure S9. MD simulations of differently charged carriers in a DOPC/cholesterol membrane. **A** Density distributions of Bundle-shaped XP 8Oc-K-B2-1:4 (1621) at different charge states within the membrane system. **B** Carrier density profiles along the centered membrane, with representative final-frame simulation snapshots shown above (yellow: membrane; blue: water) for the Bundle-shaped carrier. Insets show zoomed-in views of the interfacial

organization.

(C,D) Corresponding density distribution and membrane analysis for the U-shaped XP, analogous to panels (A) and (B), respectively. Data are presented as mean (n=3).

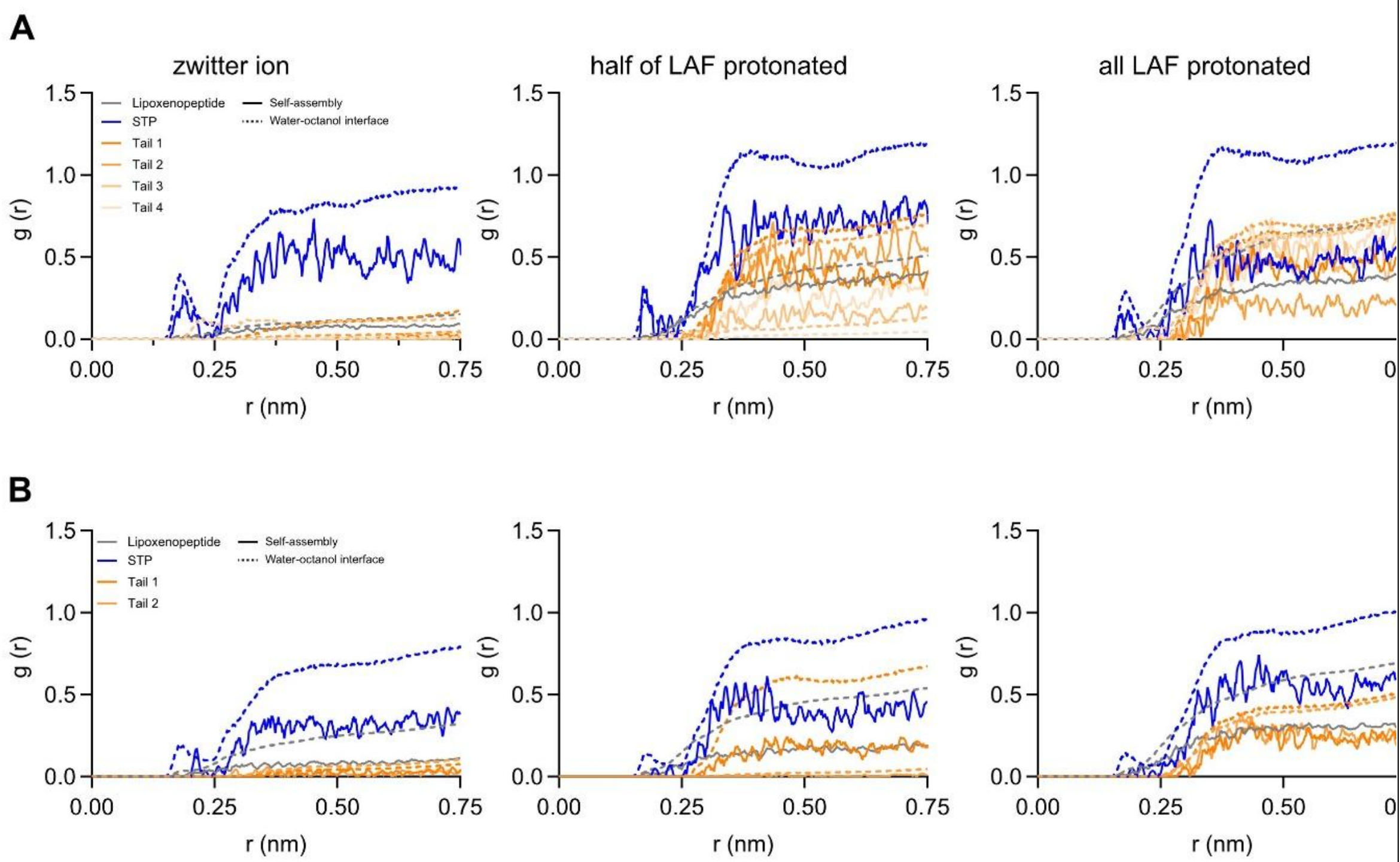

Figure S10. Comparison of radial distribution functions (RDFs) between W/O interface and self-assembly simulations. **A** Radial distribution functions of Bundle-shaped carriers obtained after 100 ns of simulation in a DOPC/cholesterol membrane and during 10 ns of W/O interface simulations. **B** Corresponding RDF analysis for U-shaped carriers. (Data are presented as mean, W/O interface: n = 3; membrane simulations: n = 3).

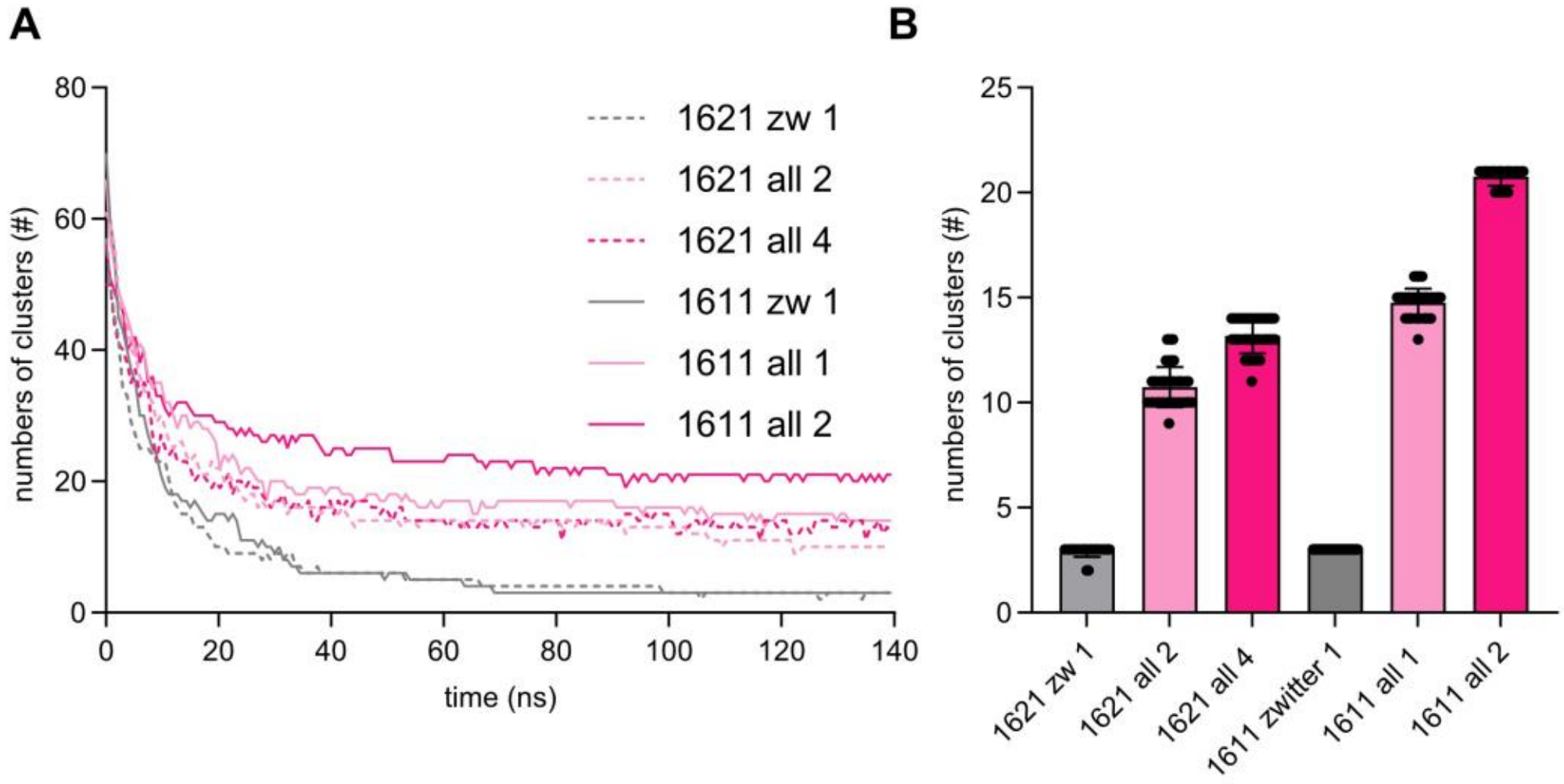

Figure S11: Self-assembly simulations of carriers in 0.9% NaCl. (**A**) Number of clusters formed over the course of a 140 ns simulation, comparing both topologies and protonation states. (**B**) Mean and individual values (dots) of the number of clusters during the last 40 ns for both topologies (n = 1).

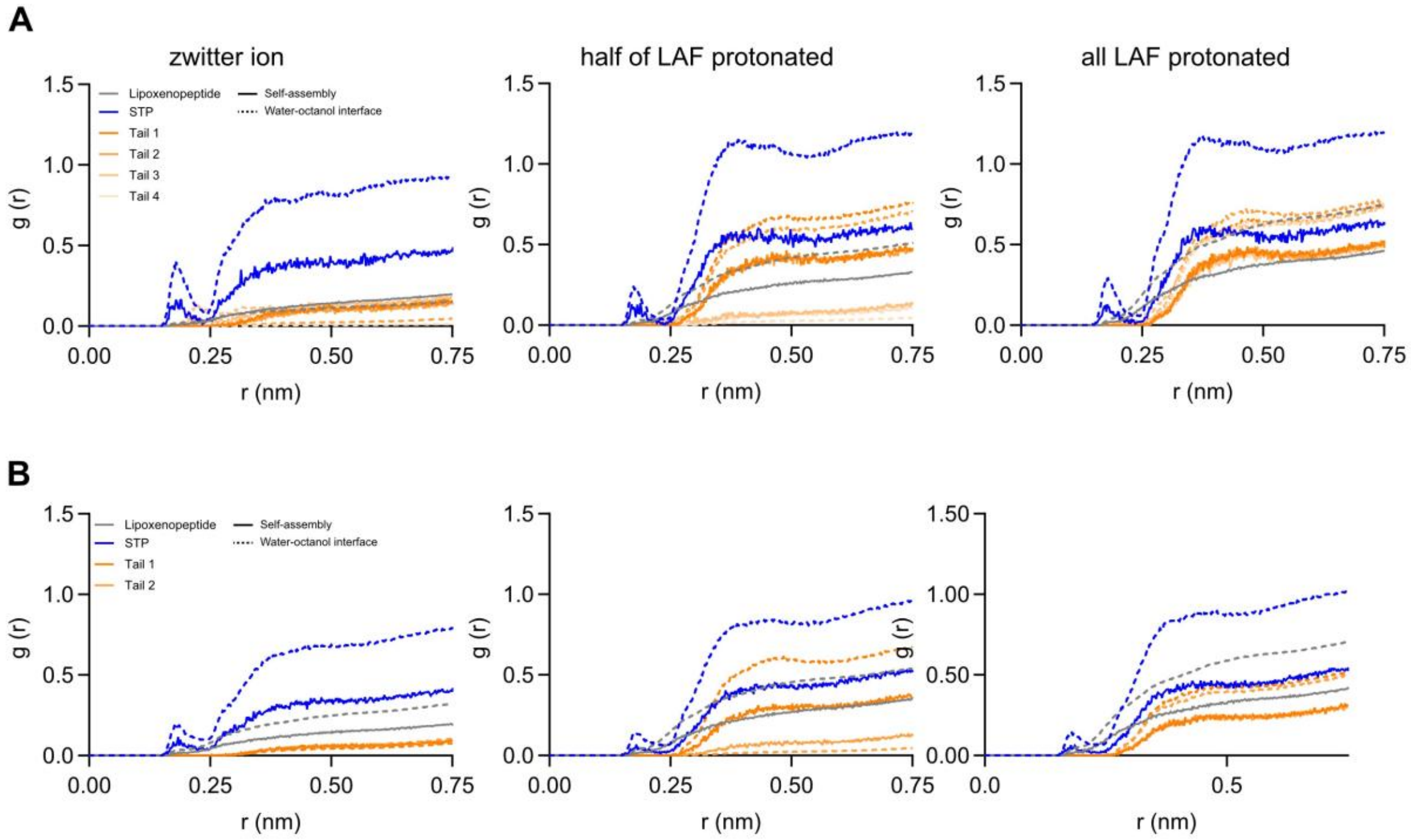

Figure S12: RDF comparison between WO interface and self-assembly simulations. (**A**) Radial distribution functions (RDFs) of Bundle XP carriers after 140 ns of self-assembly and during 10 ns of WO interface simulation. (**B**) Corresponding analysis for U-shaped XP carriers (WO interface: *n* = 3, mean shown; self-assembly: *n* = 1).

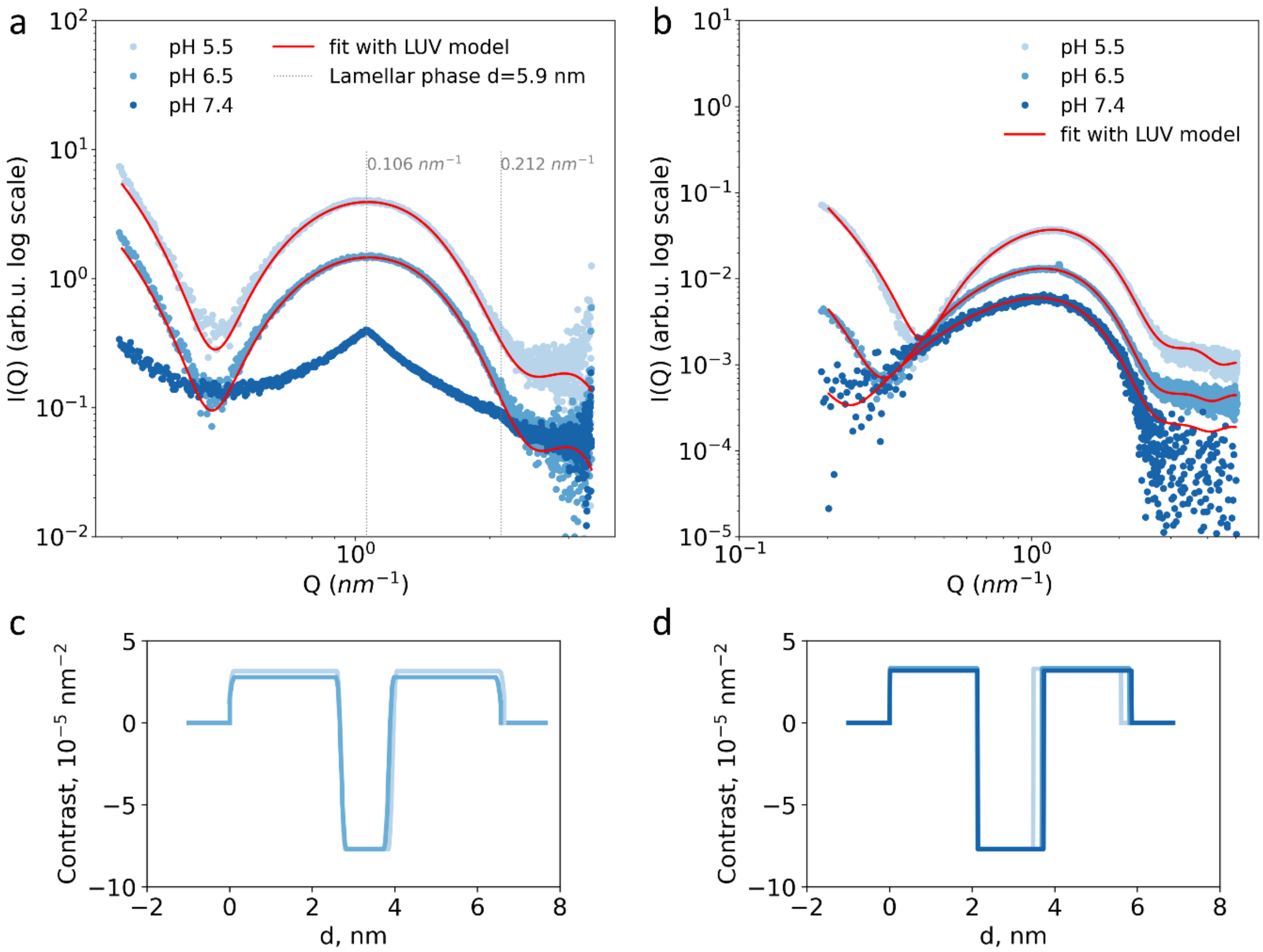

Figure S13. Self-assembly studies **a**: pH-dependent SAXS data for 12Oc-O-U1-1:2 (1827) showing fit with LUV model for pH 5.5 and 6.5, and lamellar peak positions at pH 7.4. **b**: pH-dependent SAXS data for 12Oc-O-U1-1:4 (1960) and the LUV model fits. **c**: contrast profiles extracted from the fit results for carrier 1827, **d**: contrast profiles extracted from the fit results for carrier 1960. The color-code is the same in all the plot.

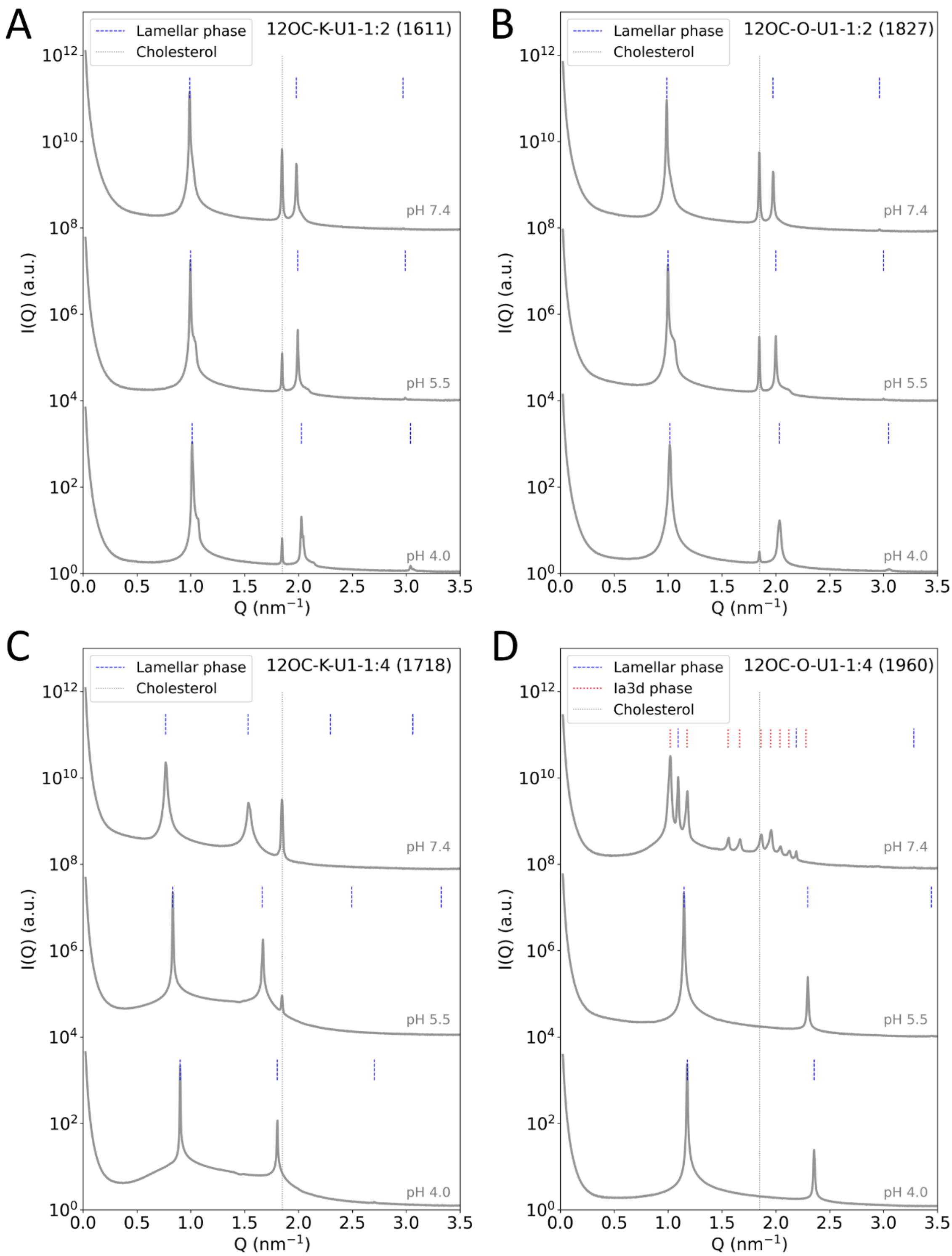

Figure S14. pH-dependent SAXS data for LAF-XP/cholesterol bulk phases for 12OC-K-U1-1:2 (1611) (A), 12OC-O-U1-1:2 (1827) (B), 12OC-K-U1-1:4 (1718) (C) and 12OC-O-U1-1:4 (1960) (D) with peak indexing. The vertical grey cholesterol line indicates the presence of the excess cholesterol crystals meaning that the LAF-XP mesophases are saturated with cholesterol.

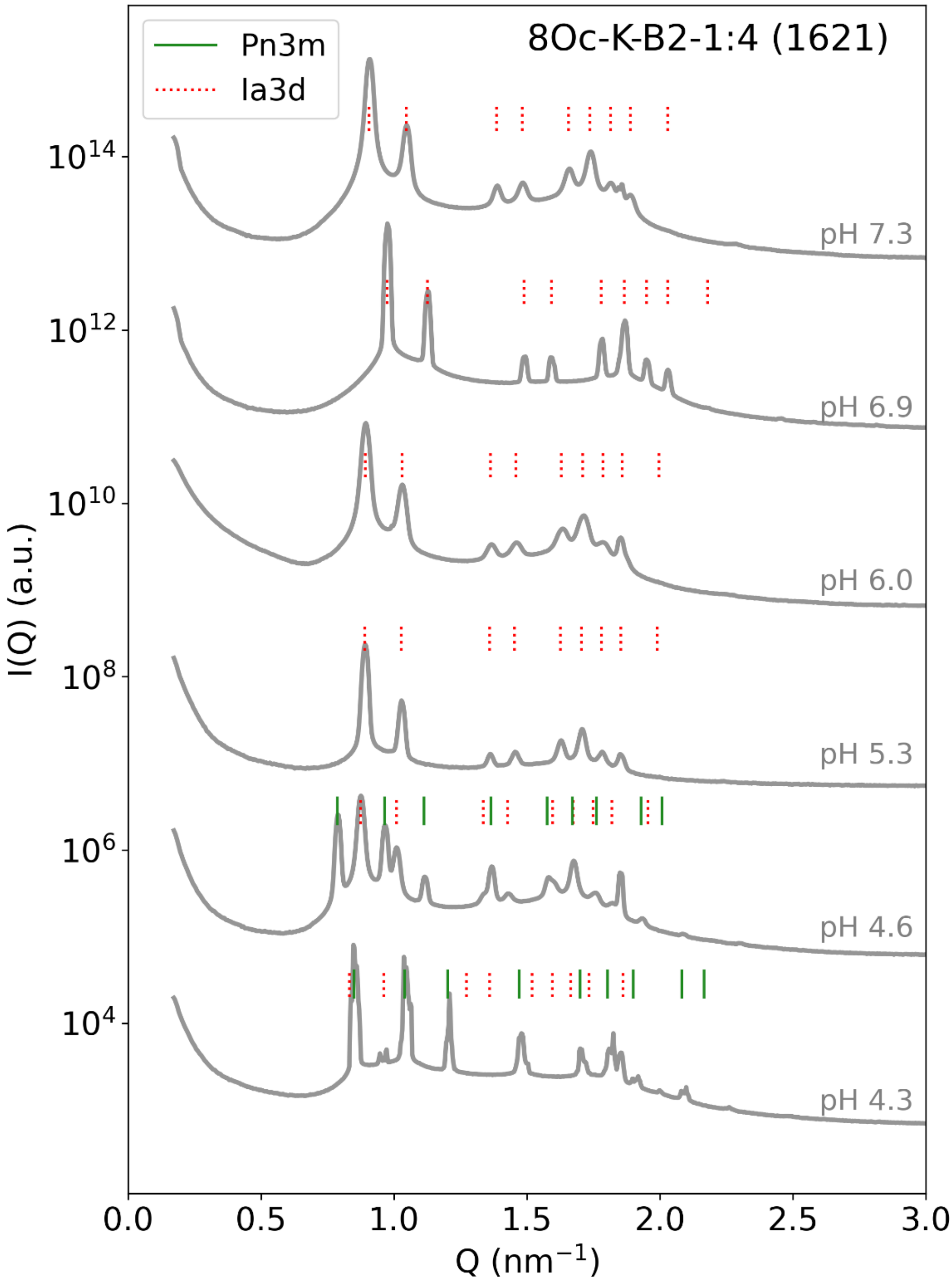

Figure S15. pH-dependent SAXS data for LAF-XP/cholesterol bulk phases for 8Oc-K-B2-1:4 (1621) with peak indexing.

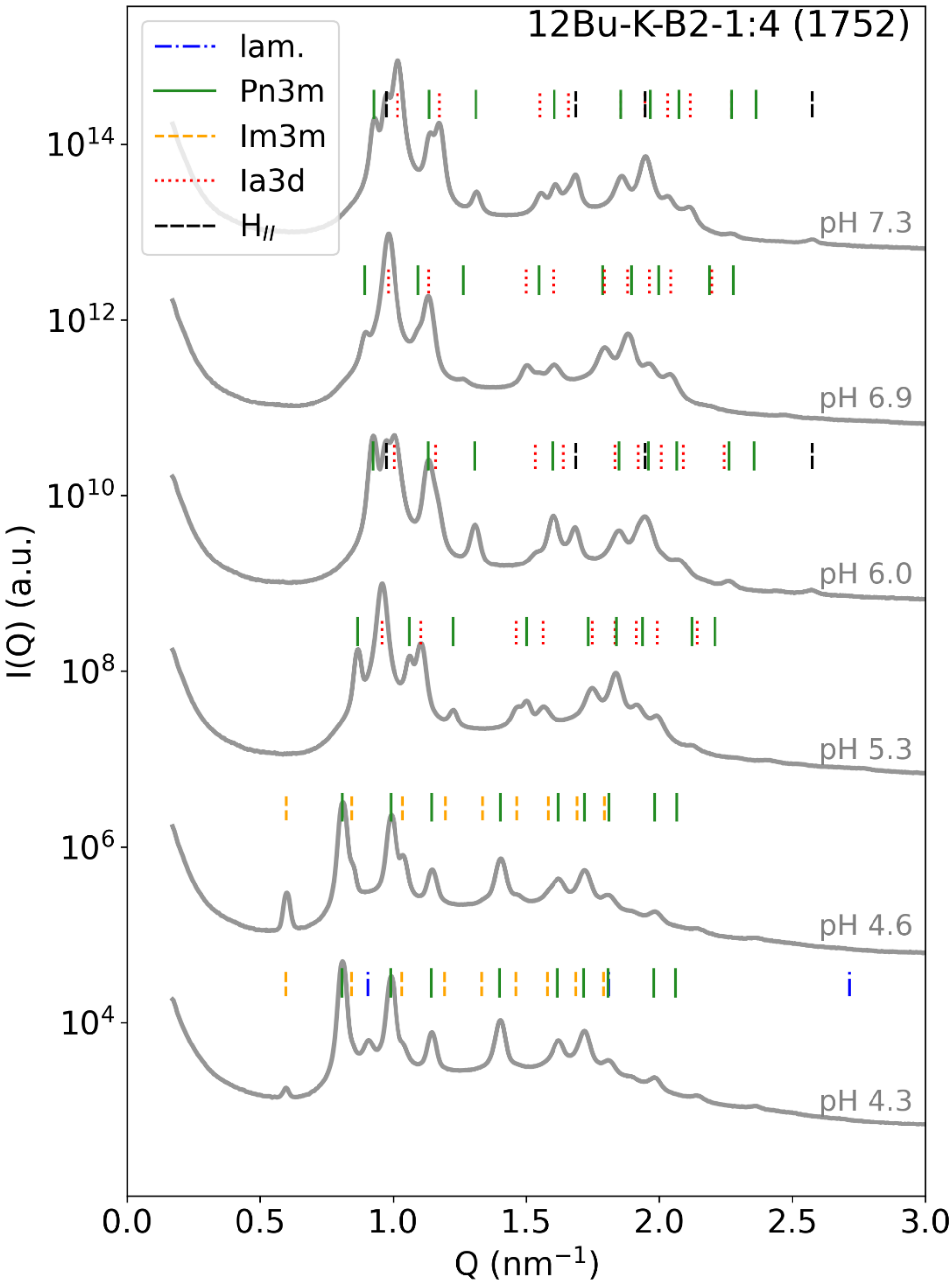

Figure S16. pH-dependent SAXS data for LAF-XP/cholesterol bulk phases for 12Bu-K-B2-1:4 (1752) with peak indexing.

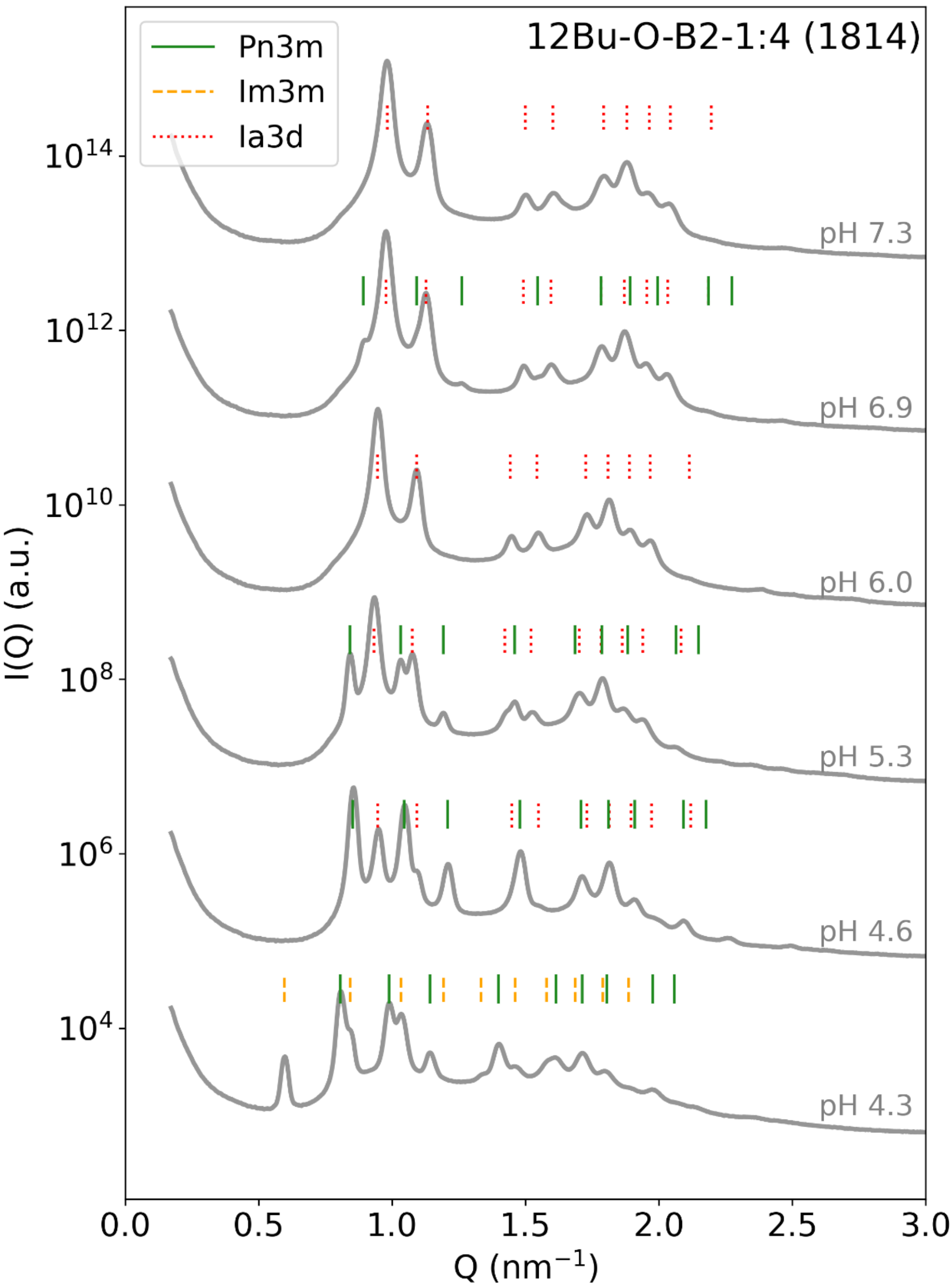

Figure S17. pH-dependent SAXS data for LAF-XP/cholesterol bulk phases for 12Bu-O-B2-1:4 (1814) with peak indexing.

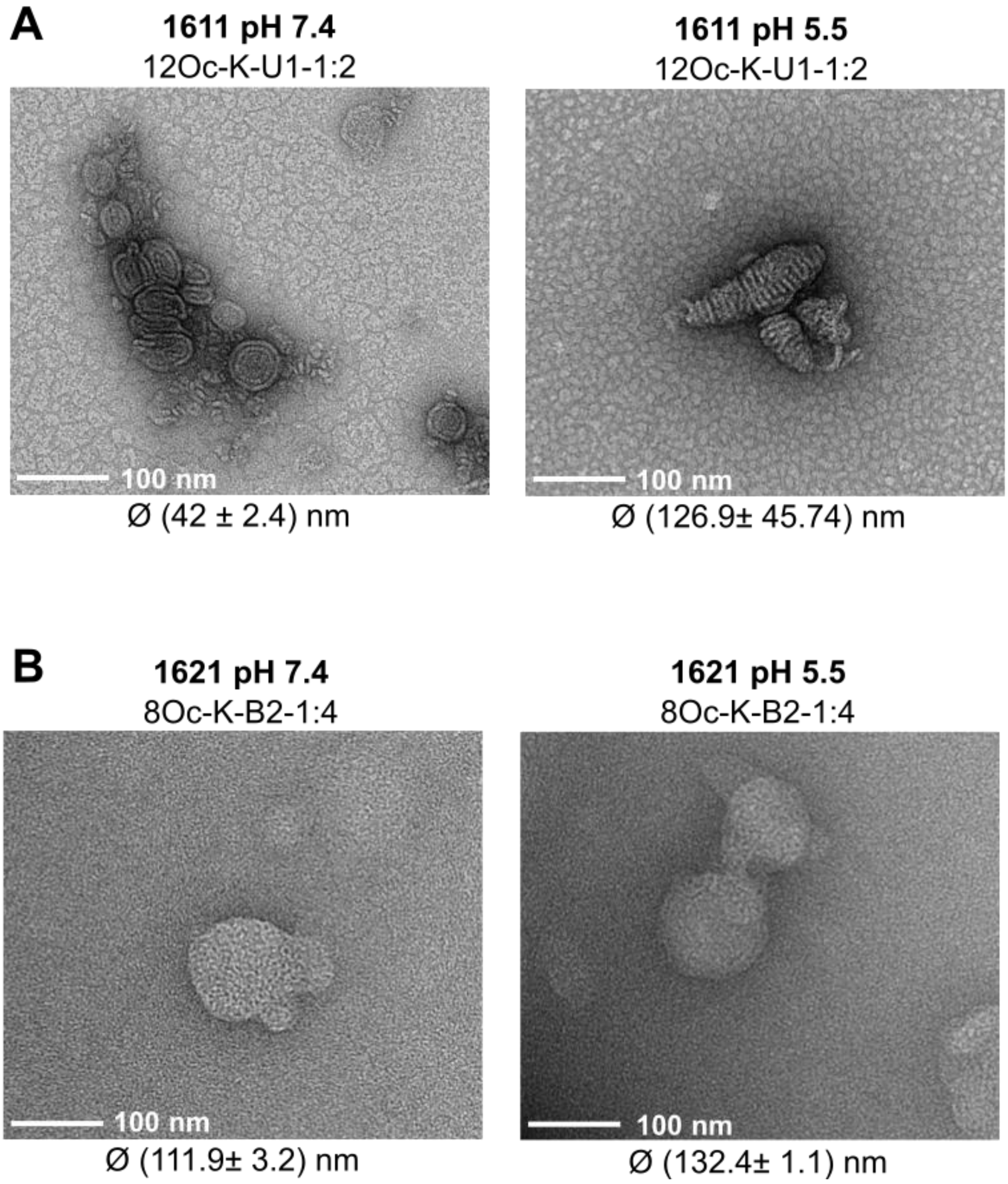

Figure S18. Representative TEM images (60k magnification) of mRNA polyplexes assembled with selected **A** U-shape and **B** Bundle XPs at pH 7.4 and pH 5.5. Average diameters are presented as diameters in nm (mean ± SD; *n*= 15 for 1611 pH 7.4, *n*= 11 for 1611 pH 5.5, *n*= 9 for 1621 pH 7.4 and *n*= 6 for 1621 pH 5.5). Images were analyzed with ImageJ 1.54g (Java 1.8.0_345).

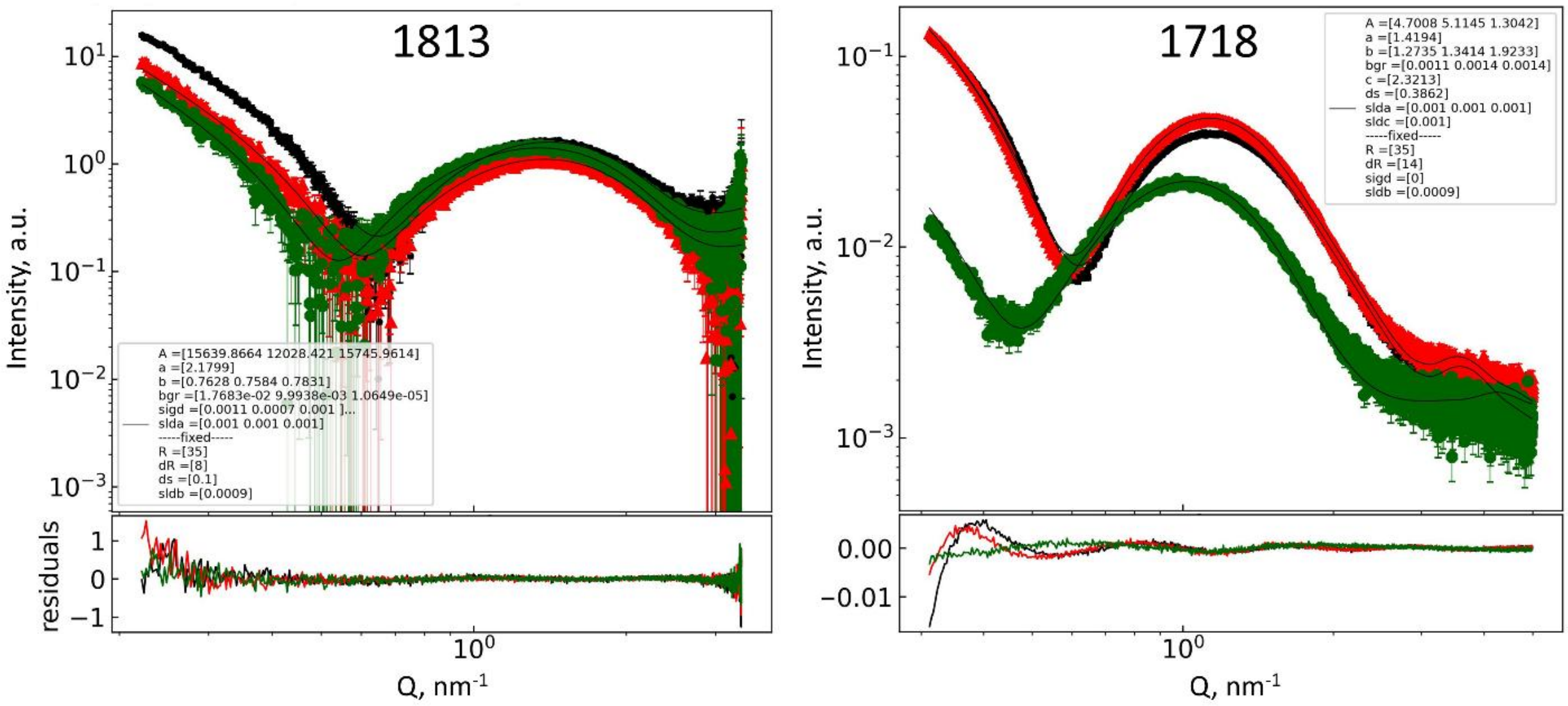

Figure S19. Representative SAXS fits for 8Oc-O-B2-1:4 (1813) and 12Oc-K-U1-1:4 (1718) using the LUV model, including absolute fit residuals. For each LAF-Stp XP, the samples measured at different pH values were fitted simultaneously (black: pH 5.5, red: pH 6.5, green: pH 7.5). The LAF-Stp XP 1718 shows the largest χ^2 value ($\chi^2 = 5.4$). This relatively high χ^2 may partially reflect systematic measurement errors that are not accounted for during data processing. Such errors can arise from variations in capillary size or sample concentration, leading to imperfect background subtraction and, consequently, underestimated intensity errors.

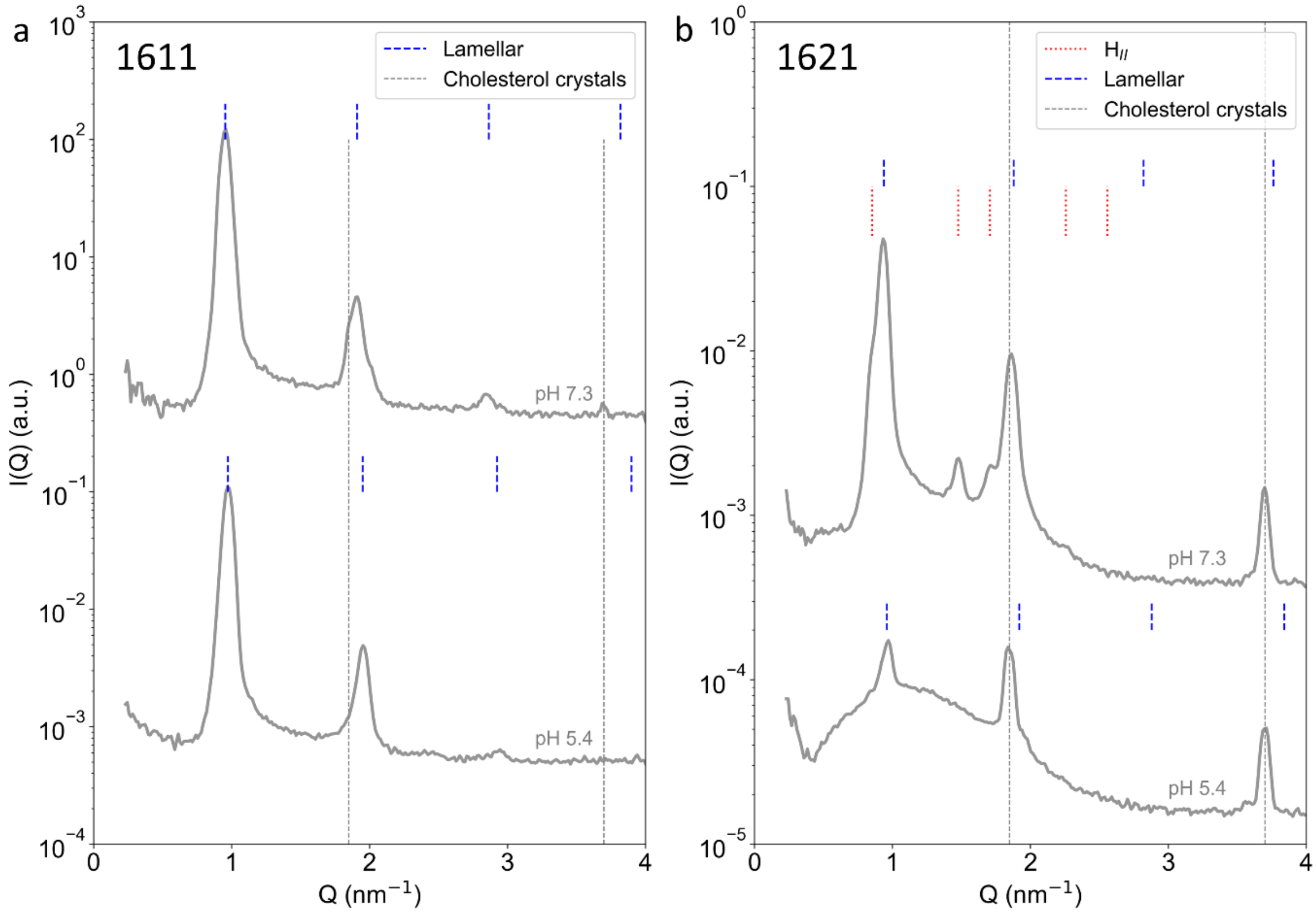

Figure S20. SAXS of the LAF-Stp XP/DOPC/cholesterol (1:1:1 molar ratio) bulk phases for the U-shape XP 1611 (12Oc-K-U1-1:2) (a) and the Bundle XP 1621 (8Oc-K-B2-1:4) (b) in HBG buffer at pH 5.4 and 7.3.

3. References

[1] Zintchenko, A., Philipp, A., *et al.*, Bioconjugate Chem. 19 (7) (2008) 1448
[2] Thalmayr, S., Grau, M., *et al.*, Adv. Mater. 35 (25) (2023) 2211105
[3] Philipp, J., Dabkowska, A., *et al.*, Proc. Natl. Acad. Sci. U.S.A. 120 (50) (2023) e2310491120
[4] Lin, Y., Wilk, U., *et al.*, Small 19 (2) (2023) 2205318
[5] Rui, Y., Wilson, D. R., *et al.*, Sci Adv 8 (1) (2022) eabk2855
[6] Blanchet, C. E., Spilotros, A., *et al.*, J. Appl. Crystallogr. 48 (Pt 2) (2015) 431
[7] Haas, S., Sun, X., *et al.*, J. Synchrotron Radiat. 30 (6) (2023) 1156
[8] Frielinghaus, H., Phys. Rev. E 76 (5) (2007) 051603
[9] Biehl, R., PLoS One 14 (6) (2019) e0218789
[10] Pabst, G., Rappolt, M., *et al.*, Phys. Rev. E 62 (3) (2000) 4000
[11] Förster, S., Timmann, A., *et al.*, J. Phys. Chem. B 109 (4) (2005) 1347
